# High-throughput sequencing identification and characterization of potentially adhesion-related small RNAs in *Streptococcus mutans*

**DOI:** 10.1099/jmm.0.000718

**Published:** 2018-03-29

**Authors:** Wenhui Zhu, Shanshan Liu, Jia Liu, Yan Zhou, Huancai Lin

**Affiliations:** Guanghua School of Stomatology, Hospital of Stomatology, Sun Yat-Sen University, Guangdong Provincial Key Laboratory of Stomatology, Guangzhou 510055, PR China

**Keywords:** *Streptococcus mutans*, small RNAs, adherence, clinical strains, dental caries

## Abstract

**Purpose:**

Adherence capacity is one of the principal virulence factors of *Streptococcus mutans*, and adhesion virulence factors are controlled by small RNAs (sRNAs) at the post-transcriptional level in various bacteria. Here, we aimed to identify and decipher putative adhesion-related sRNAs in clinical strains of *S. mutans.*

**Methodology:**

RNA deep-sequencing was performed to identify potential sRNAs under different adhesion conditions. The expression of sRNAs was analysed by quantitative real-time PCR (qRT-PCR), and bioinformatic methods were used to predict the functional characteristics of sRNAs.

**Results:**

A total of 736 differentially expressed candidate sRNAs were predicted, and these included 352 sRNAs located on the antisense to mRNA (AM) and 384 sRNAs in intergenic regions (IGRs). The top 7 differentially expressed sRNAs were successfully validated by qRT-PCR in UA159, and 2 of these were further confirmed in 100 clinical isolates. Moreover, the sequences of two sRNAs were conserved in other *Streptococcus* species, indicating a conserved role in such closely related species. A good correlation between the expression of sRNAs and the adhesion of 100 clinical strains was observed, which, combined with GO and KEGG, provides a perspective for the comprehension of sRNA function annotation.

**Conclusion:**

This study revealed a multitude of novel putative adhesion-related sRNAs in *S. mutans* and contributed to a better understanding of information concerning the transcriptional regulation of adhesion in *S. mutans*.

## Introduction

Dental caries is a dental biofilm-related infectious disease that affects 95 % of the global population at some point over the course of their lives [1, 2]. Despite significant advances in dentistry, caries remains a serious public health problem that impacts on the daily life of affected individuals, particularly in developing countries [3], and more efficacious prevention strategies are required to reduce its incidence.

*Streptococcus mutans* is the predominant pathogen implicated in dental caries. This bacterium depends on a ‘biofilm lifestyle’ for survival and persistence in its natural ecosystem (dental plaques) [4]. The adherence of *S. mutans* to dental surfaces constitutes the initial step of biofilm formation, which is mediated by both sucrose-independent and sucrose-dependent mechanisms [5, 6]. Although biofilm research of *S. mutans* has been performed in some detail, the transcriptional regulatory response of *S. mutans* adherence is not yet completely understood.

Small RNAs (sRNAs) are emerging as an important transcriptional regulators for bacteria because they facilitate rapid adaptation to changing environmental conditions [7]. This adaptation is particularly crucial in the setting of oral dental biofilms, which are exposed daily to regular changes in nutrient availability [8]. Over the past few decades, studies of RNA-mediated regulation of small RNA molecules have revealed the paramount roles played by these molecules, both directly and indirectly, in bacterial biofilm formation. For example, in *Escherichia coli*, sRNAs facilitate biofilm formation by modulating the expression of mRNA targets [9–12]. The sRNA FasX regulates the expression of adhesive pili in group A streptococcus (GAS) [13].

Recently, a variety of sRNAs have been identified in *S. mutans*. Lee and Hong reported more than 900 potential miRNA-size small RNAs (msRNAs) in *S. mutans*. [14]. Additionally, specific sRNAs of a small size (18–50 nucleotides) are specifically induced under acid stress conditions, as revealed in our previous studies [15]. However, all previous studies were performed with planktonic cells, and very little is known regarding the sRNAs obtained from attached cells, which exhibit different physiological properties from their planktonic counterparts [16, 17]. Furthermore, the characteristics of adhesion-related sRNAs have not been extensively investigated in *S. mutans.* Therefore, it is essential to investigate sRNAs in *S. mutans* that are related to the attachment of cells to a surface in order to better understand the mechanisms underlying *S. mutans* biofilm formation.

In the present study, strand-specific sequencing was employed to gain insights into the landscape of sRNAs ranging from 18 to 150 nt in length in adherent cells of *S. mutans*. Differentially expressed sRNAs associated with adhesion were identified in UA159 and clinical strains. Moreover, GO and KEGG functional annotation was performed to broaden our understanding of the potential functional characteristics of sRNAs in *S. mutans*. The data obtained indicate that specific adherence-related sRNAs may play regulatory roles in the adherence process of *S. mutans*.

## Methods

### Sample collection and isolation of *S. mutans*

We obtained clinical strains from an epidemiological survey that was conducted in Guangdong Province, People's Republic of China, in 2015. Children with no systematic illness or antibiotic intake for at least 1 month prior to the study were included. The study protocol was approved by the Ethics Committee of the Guanghua School of Stomatology, Sun Yat-sen University (ERC-[2015]-09). All parents of the participating children were informed of the study and provided written consent.

The clinical strains of *S. mutans* were isolated according to the method described by Yu *et al.* [18]. A total of 215 clinical strains were isolated from 215 children with different caries status. We randomly selected 100 clinical strains for further study.

### Bacterial strains and culturing conditions

*S. mutans* UA159 (ATCC 700610) and the 100 purified clinical strains were grown in brain heart infusion (BHI) broth (Oxoid, Basingstoke, UK) overnight under anaerobic conditions (80 % N_2_ and 20 % CO_2_) at 37 °C. The optical density (OD) at 600 nm of overnight cultures of the strains was determined by an ultraviolet spectrophotometer system (VAISALA, Helsinki, Finland). To screen out sucrose, the UA159 suspensions (OD_600_=0.7) were inoculated at 1 : 20 into 96-well plates containing fresh BHI with 0, 0.5, 1 and 2 % sucrose and grown anaerobically for 4 h at 37 °C. For sequencing, overnight cultures of the UA159 suspension were inoculated into a Petri dish containing fresh BHI with 1 or 0 % sucrose and grown anaerobically for 4 h at 37 °C. For a correlation analysis between sRNA and mRNA, 100 clinical strain suspensions (OD_600_=0.7) were sub-cultured at 1 : 20 in fresh BHI with 1 % sucrose for 4 h.

### RNA extraction

For RNA extraction, suspensions of 100 *S. mutans* clinical strains and UA159 were inoculated into Petri dishes containing fresh BHI with 1 % sucrose and grown anaerobically for 4 h at 37 °C. RNAprotect Bacteria Reagent (Qiagen, Valencia, CA, USA) was used to stabilize the total RNA before extraction. Two volumes of RNAprotect Bacteria Reagent were added directly to one volume of bacterial culture. Then, adherent UA159 cells and clinical strains were harvested and washed twice with PBS. The cells were firstly incubated into the lysis buffer [30 mM Tris-HCl, 1 mM EDTA and 20 mg ml^−1^ lysozyme (pH 8.0)] with gentle agitation for 45 min at 37 °C. The total RNA of the adherent cells was purified using a miRNeasy Mini kit (Qiagen, Valencia, CA, USA) according to the manufacturer’s recommended protocol. A Thermo Scientific Nanodrop 2000 (NanoDrop Technologies, NC, USA) and an Agilent 2100 system (Agilent Technologies, Santa Clara, CA, USA) were used for the assessment of RNA quality and quantity.

### Small RNA library construction and deep sequencing

Total RNA was isolated as described above. cDNA libraries were constructed according to the Illumina TruSeq small RNA sample preparation protocol. Gel extraction and ethanol precipitation were performed to isolate RNA fragments ranging from 18 to 150 nt in size. Small RNAs were ligated to a pair of adaptors (5′: 5′-GUUCAGAGUUCUACAGU CCGACGAUC-3′ and 3′: 5′-UGGAAUUUCUCGGGUG CCAAGG-3′), subjected to reverse transcription-PCR to obtain single-stranded cDNAs and then amplified by PCR. The amplified cDNA fragments were sequenced using Illumina technology on a HiSeq 4000 (Illumina, San Diego, CA, USA) by BGI (Shenzhen, People's Republic of China).

### Bioinformatics analysis of candidate sRNAs

Raw sequence reads were processed into clean reads (BioProject ID PRJNA408134) using Illumina Pipeline software as previously reported [19]. In brief, all low-quality reads, reads from which adapter sequences had been trimmed and reads shorter than 18 nucleotides were removed from the raw sequence reads. Sequences that mapped to the nr database or that were predicted with potential coding capacities by the Coding Potential Calculator (http://cpc.cbi.pku.edu.cn/) were filtered out. The remaining 18 to 150 nt high-quality clean sequences that mapped to intergenic regions (IGRs) and to the antisense to mRNA (AM) sequences using the SOAP program were selected. To remove rRNA and tRNA, the annotated sequences were searched against the NCBI GenBank database (http://www.ncbi.nlm.nih.gov/nuccore/AE014133) and the Rfam database (http://www.sanger.ac.uk/software/Rfam).

We predicted the structures of candidate sRNAs using the RNAfold web server (http://rna.tbi.univie.ac.at/cgi-bin/RNAWebSuite/RNAfold.cgi). The promoters of sRNAs were predicted using BPROM (http://linux1.softberry.com/berry.phtml) [20]. The ENA browser was used to view the region surrounding sRNAs in *S. mutans* [21]. To compare the differential expression of sRNAs between the 1 and 0 % sucrose libraries, DEseq2 was implemented according to Love *et al*.’ s method [22]. Fold changes were calculated based on the following equation: log_2_ ratio=log_2_ (1 % sucrose reads/0 % sucrose reads). Significant differences in sRNA expression were assigned to sequences with *P*<0.05 and |log_2_ ratio|≥1. We used IntaRNA to predict sRNA targets; this predicts targets based on hybrid free energy and feasible binding free energy [23]. Functional annotation was performed with the GO database and Database for Annotation, Visualization and Integrated Discovery (DAVID) software.

### Quantitative real-time PCR (qRT-PCR) verification of sRNA expression

Reverse transcription quantitative PCR with SYBR Green was performed to verify the expression of seven significantly differentially expressed sRNAs and nine target mRNAs in UA159 and the clinical strains. The primers used in this study are listed in Table S1. cDNA was synthesized using a Mir-X miRNA First-Strand Synthesis kit (Takara and Clontech, Dalian, People's Republic of China) according to the manufacturer’s recommended protocol. Finally, qRT-PCR was performed on a LightCycler 480 Real-Time System with a SYBR Premix Ex *Taq* II kit (Takara and Clontech, Dalian, People's Republic of China). The reaction conditions were 95 °C for 30 s followed by 40 cycles of 95 °C for 15 s and 60–63 °C for 30 s. The PCR products were then analysed by 3 % agarose gel electrophoresis with the GeneRuler 100 bp DNA Ladder (Dongsheng Biotech, Guangzhou, Republic of China) and confirmed by Sanger sequencing (Sangon Biotech, Shanghai, People's Republic of China). The expression of sRNAs and mRNAs were normalized to the 16S rRNA levels to obtain ΔCt values. For analysis of the relative expression levels of sRNA0187 and sRNA0593 in high-adherence clinical strains and low-adherence strains, the mean expression of sRNA0187 or sRNA0593 in the LG group is defined as 1.0. The expression of each gene was determined in triplicate. The expression levels were calculated using the 2−^ΔΔCt^ method. A correlation analysis was performed using the Ct values of each sRNA and mRNA.

### Adherence analysis

An adherence assay was performed using crystal violet staining according to the Christensen *et al*.’s method [24]. The assay was performed in triplicate and repeated three times. The mean adherence values for each strain were calculated to represent their adhesion capacities. We first sorted the adherence value of 100 clinical strains. Then, the average of the two most intermediate values was set as the median adherence value. The 100 clinical strains were divided into a high-adherence group (HG) and a low-adherence group (LG) based on the median adherence value of the *S. mutans* clinical strains.

### Fluorescent staining

To ascertain changes in bacterial viability in the initial stage of biofilm formation over time, we used confocal laser scanning microscopy (CLSM; TSP SP2, Leica, Germany) based on *in situ* labelling of the bacteria using a LIVE/DEAD BacLight Bacterial Viability kit (Molecular Probes) according to the method developed by Meng-Ying Mao [25]. For the analysis of exopolysaccharides production, 1 µM Alexa Fluor 647 (Invitrogen, Eugene, OR, USA) and 2.5 µM SYTO 9 (Invitrogen, Carlsbad, CA, USA) [26] were used to label dextran and bacterial cells, respectively. After staining, the biofilm cells were washed three times with physiological saline and observed under a confocal microscope. Under CLSM, the live bacteria were stained green, and the dead bacteria or exopolysaccharides were stained red. COMSTAT was used to analyse the bio-volume (µm^3^/µm^2^), mean thickness (µm) and EPSs. The three-dimensional architecture of the biofilms was analysed using Imaris 8.0.2 (Bitplane, Zurich, Switzerland). Three independent biofilm experiments were performed, and images of three random fields were collected for each group.

### Statistical analyses

IBM SPSS 20.0 (IBM, Armonk, NY, USA) was used to analyse the data. The means and standard deviations of all continuous variables were computed. First, the data were assessed for normal distribution and sphericity. The Mann–Whitney test and repeated-measures analysis of variance were used (*P*<0.05). For correlation testing, the Spearman rank correlation coefficient was used with a *P*-value of <0.05.

## Results

### Overview of the process of adhesion in *S. mutans*

To identify the sRNAs involved in adhesion in *S. mutans*, UA159 was allowed to adhere to the surface of Petri dishes under four different adhesion conditions (0 % sucrose, 0.5 % sucrose, 1 % sucrose and 2 % sucrose) for 1, 2, 3 or 4 h, respectively.

**Fig. 1.**
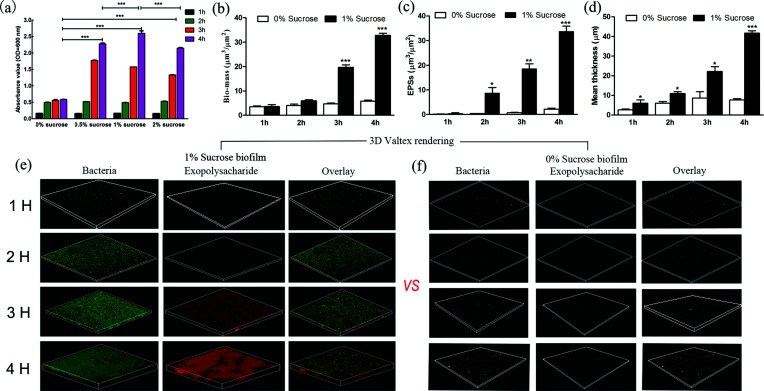
Effects of sucrose on *S. mutans* adhesion. (a) A crystalline violet staining experiment was performed to determine the *S. mutans* adhesion under different sucrose concentrations and at different time points. (b) The bio-volume and (c) mean thickness of biofilms were both calculated. (d) Representative three-dimensional images of bacteria in the biofilms. (e) Representative three-dimensional images of EPS production and distribution in the biofilm architecture. Images were magnified 20-fold. The error bars represent the standard deviations. *, significant difference at *P*<0.05; **, *P*<0.01; ***, *P*<0.001.

The adherence of *S. mutans* increased gradually over time, and the maximum adherence of *S. mutans* was recovered at 4 h under each sucrose concentration. We then compared the adherence values at different sucrose concentrations at 4 h. The maximum effective concentration of sucrose for adherence during 4 h biofilm formation was 1 % (*P*<0.001) (Fig. 1a). Moreover, the process of biofilm formation (1, 2, 3 and 4 h) under 1 and 0 % sucrose conditions was visualized using the LIVE/DEAD and EPS fluorescent staining, which are shown separately in Fig. 1(e, f). Consistent with the crystal violet staining results, the bacteria bio-volume, mean thickness and EPSs of 1 % sucrose increased gradually with time in the CLSM study, but this finding was not observed with the 0 % sucrose biofilm (Fig. 1b–d). The bacteria bio-volume, mean thickness and EPSs of the 1 % sucrose biofilm were at least 5.63-fold higher than those of the 0 % sucrose biofilm at 4 h (*P*<0.001) (Fig. 1b–d).

### sRNA sequencing reveals the putative sRNAs in *S. mutans*

We constructed six strand-specific cDNA libraries (18–150 nt), including three control libraries (control-1, control-2 and control-3) and three 1 % sucrose-treated libraries (scr-1, scr-2 and scr-3) at 4 h post-inoculation. A total of 35 153  906 average raw reads were obtained from the six libraries. For each sequencing library, the raw read count reached more than 3 million. In addition, at least 26 616 738 clean reads (84.27 % of raw reads) and 25 499 778 clean reads (83.7 % of raw reads) were generated from the 0 and 1 % sucrose libraries (Table 1), respectively, indicating the high quality of the sRNA.

**Table 1.** Mapping statistics of RNA sequencing reads

	Control-1	Control-2	Control-3	Scr-1	Scr-2	Scr-3
Raw reads	36 348 356	37 069 332	31 548 110	37 038 536	38 453 872	30 465 232
*N* reads	10 090	10 104	7 562	9 754	8 546	7 374
Low-quality reads	13 772	23 794	13 838	12 798	10 942	12 130
Adapter reads	5 033 030	4 324 484	4 945 972	5 745 304	11 082 770	4 945 950
Clean reads	31 291 464	32 710 950	26 616 738	31 270 680	27 351 614	25 499 778
Clean reads/raw reads	86.09 %	88.24 %	84.27 %	84.43 %	71.13 %	83.70 %

**Fig. 2.**
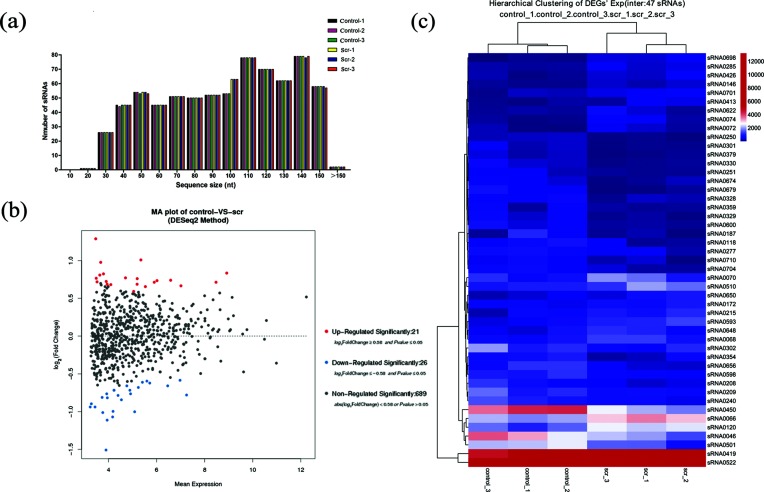
Overall distribution of the small RNA compositions of the generated libraries and differential expression analysis of candidate sRNAs. (a) Length distributions and abundances of small RNAs in the six samples. The sRNA lengths (nt) are shown on the *x*-axis and the numbers of sRNAs are presented on the *y*-axis.(b) An MA plot shows the distribution of fold changes in sRNA expression. Upregulated genes with a log_2_- fold change of ≥0.58 and *P*-value of ≤0.05 are indicated in red, and downregulated genes with a log_2_-fold change of ≤−0.58 and a *P*-value of ≤0.05 are indicated in blue. (c) Heat maps were produced from a hierarchical cluster analysis and showed 47 differentially expressed sRNAs. The colour scale on the right shows the relative expression levels of the sRNAs across all the samples: red represents expression >3000 and blue represents expression <3000. (Control indicates the 0 % sucrose condition and scr indicates the 1 % sucrose condition.)

A total of 736 potential candidate sRNAs were found during the initial stage of biofilm formation, and these included 352 sRNAs (47.83 % of the total sRNAs) located on the antisense to mRNA and 384 sRNAs (52.17 % of the total sRNAs) found in the IGR (Table S2). All sRNAs were shared between the 0 and 1 % sucrose libraries. The lengths of these candidate sRNAs, ranging from 18 to 150 nt, are shown in Fig. 2(a). The length distribution and abundance of the small RNA were roughly the same throughout the six libraries and were uniformly distributed, except at an RNA sequence size of 100 nt, where more sRNAs were identified in the sucrose-treated group compared with the control group.

### Confirmation of differentially expressed sRNAs during the initial stage of biofilm formation

A comparative analysis of sRNAs identified from the 0 % sucrose library and the 1 % sucrose library revealed that 6.4 % (47 of 736) of the sRNAs were differentially expressed after restricting our analysis to results with *P*≤0.05 and |log_2_ ratio|≥0.58 (Fig. 2b). Among these 47 sRNAs, 21 were upregulated and 26 were downregulated in the 1 % sucrose library compared with the 0 % sucrose library (Fig. 2c). We chose the seven most significantly differentially expressed sRNAs (DE sRNAs with a fold change (|log_2_ ratio|)>1) for further study. Of the seven DE sRNAs, two were upregulated and five were downregulated after 1 % sucrose treatment (Table 2).

**Table 2.** Seven most differentially expressed sRNAs in the 1 % sucrose (Scr) and 0 % sucrose (control) libraries The fold-change represents the relative expression level of normalized sRNAs between the libraries. *P* is the significance of the differential expression of the differentially expressed sRNAs (*P*<0.01) between libraries. The fold-change (log_2_ Scr/control)>1 or fold-change <−1.

sRNA ID	Length	Scr count	Control count	Fold-change (log_2_ scr/control)	*P*-value	Up/down
sRNA0593	103	57.643	28.705	1.006	<0.001	Up
sRNA0698	49	17.259	7.077	1.286	<0.001	Up
sRNA0330	25	9.518	19.221	−1.014	0.004	Down
sRNA0329	38	10.444	22.581	−1.113	0.001	Down
sRNA0187	118	12.403	26.028	−1.069	0.003	Down
sRNA0679	53	8.806	25.095	−1.511	<0.001	Down
sRNA0656	113	23.977	47.995	−1.001	<0.001	Down

**Fig. 3.**
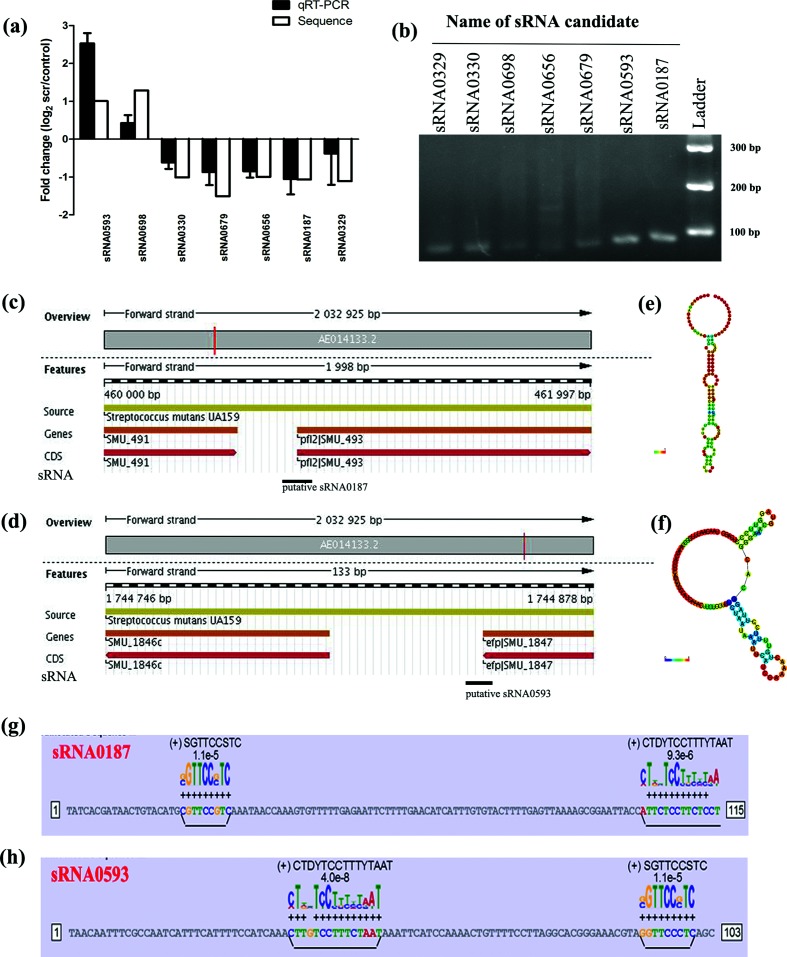
Validation of the top seven differentially expressed sRNAs by qRT-PCR. (a) Validation of the top seven differentially expressed sRNAs. The *x*-axis represents the sRNAs, and the *y*-axis indicates the fold change between the 1 % sucrose library and the 0 % sucrose library calculated as log_2_ (1 % sucrose libraries/0 % sucrose libraries). Three biological replicates were used. (b) Electrophoresis of the PCR products of seven sRNAs on a 3 % agarose gel. Lane 1, GeneRuler Ultra 100 bp DNA Ladder; lanes 2–8, confirmed sRNA candidates. (c, d) Genetic context of sRNA0187 and sRNA0593. ENA browser view of the region surrounding sRNA0187 and sRNA0593 in *S. mutans*. (e, f) sRNA0187 and sRNA0593 secondary structure predicted by RNAfold. (g, h) BPROM was used to predict the potential promoters for sRNA0187 and sRNA0593.

To validate the presence of the seven DE sRNAs predicted by RNA-Seq, we performed qRT-PCR separately to investigate the relative expression levels of the top seven DE sRNAs (sRNA0187, sRNA0329, sRNA0330, sRNA0593, sRNA0656, sRNA0679 and sRNA0698). The qRT-PCR data revealed a degree of agreement with the expression profiles obtained by deep sequencing (Fig. 3a). Some sRNAs, such as sRNA0329, sRNA0330, sRNA0656, sRNA0698 and sRNA0679, exhibited lower relative fold changes, as determined by qRT-PCR. However, qRT-PCR detected a higher relative fold change and a similar fold change compared with the deep sequencing results for sRNA0593 and sRNA0187, respectively. Thus, sRNA0187 and sRNA0593 were selected for further study. This observed variance likely resulted from biological differences between samples. To further confirm the accuracy of the qRT-PCR results, we subsequently verified these PCR products using Sanger sequencing (Fig. 3b). According to this Sanger sequencing, all of the clone sequence matched the target sRNA sequences, except for sRNA0656, which was not detected by cloning.

### Characterization of sRNAs in *S. mutans* and functional annotation

**Fig. 4.**
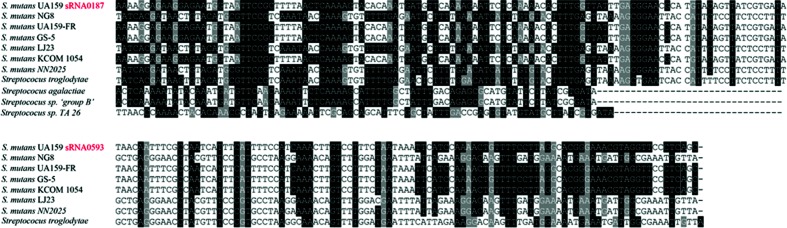
Multiple sequence alignment of putative homologues to confirmed sRNA0187 and sRNA0593 in *S. mutans.*

To determine whether similarly putative sRNAs were identified in other bacteria, we compared each of the seven DE sRNAs sequences using blastn. The results are shown in Figs 4 and S2. A sequence was only considered to be conserved when the coverage between the query and subject sequences was higher than 75 % and the nucleotide identity was higher than 65 % (E-value=10^−5^, word=11). Some confirmed sRNAs (sRNA0187, sRNA0593 and sRNA0656) might be conserved in other *Streptococcus* species, primarily in the *Streptococcus troglodytae* and *Streptococcus agalactiae*, which suggested that they might play conserved roles in these closely related species.

**Fig. 5.**
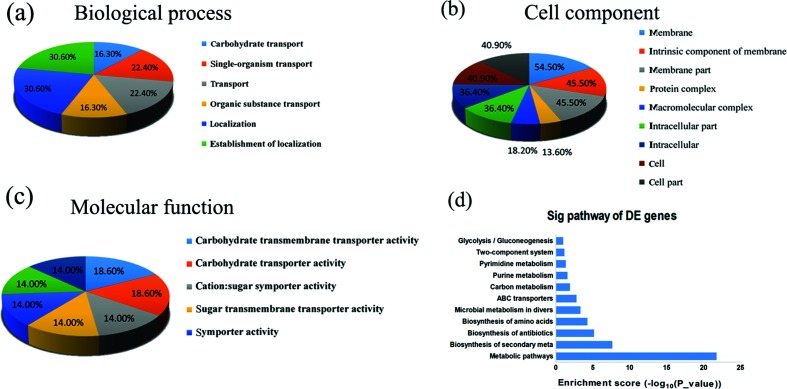
The GO and KEGG analysis for predicted target mRNAs of sRNA0187 and sRNA0593. (a–c) GO terms identified in the GO analysis for correlated target mRNAs in the biological process, cellular component and molecular function categories, with corrected *P*-value ≤1. (d) Biological pathways obtained from the KEGG analysis with *P*-value <0.05.

Secondary structures are important for the stability of sRNAs, and the secondary structures of all 736 candidate sRNAs were predicted by RNAfold. Fig. 3(e, f) focused solely on the secondary structures of sRNA0187 and sRNA00593. The location information for the 736 candidate sRNAs is shown in Table S2. Both sRNA0187 and sRNA0593 were mapped to the AM region. sRNA0187 is located between SMU.491 and SMU.493 (Fig. 3c), whereas sRNA0593 is found located upstream of the SMU.1847 and downstream of the SMU.1846c (Fig. 3d). We predicted their putative promoters for sRNA0187 and sRNA0593 using BPROM, as shown in Fig. 3(g, h).

or a preliminary exploration of the potential functional characterizations of putative adhesion-related sRNAs in the process of adhesion, we performed GO term and KEGG pathway annotation of the target mRNAs of sRNA0187 and sRNA0593 using the GO and KEGG databases (Fig. 5). We found that 16.3 % of the differentially expressed genes targeted by sRNA0187 and sRNA0593 correspond to carbohydrate transporter activity. KEGG analysis identified 11 pathways that were significantly regulated (*P*≤0.05) and that were potentially associated with functions of *S. mutans*. Some of these pathways, especially the metabolic pathways, carbon metabolism and two-component system are vital to biofilm formation.

### Differential expression of sRNAs in clinical strains with different adherence values

**Fig. 6.**
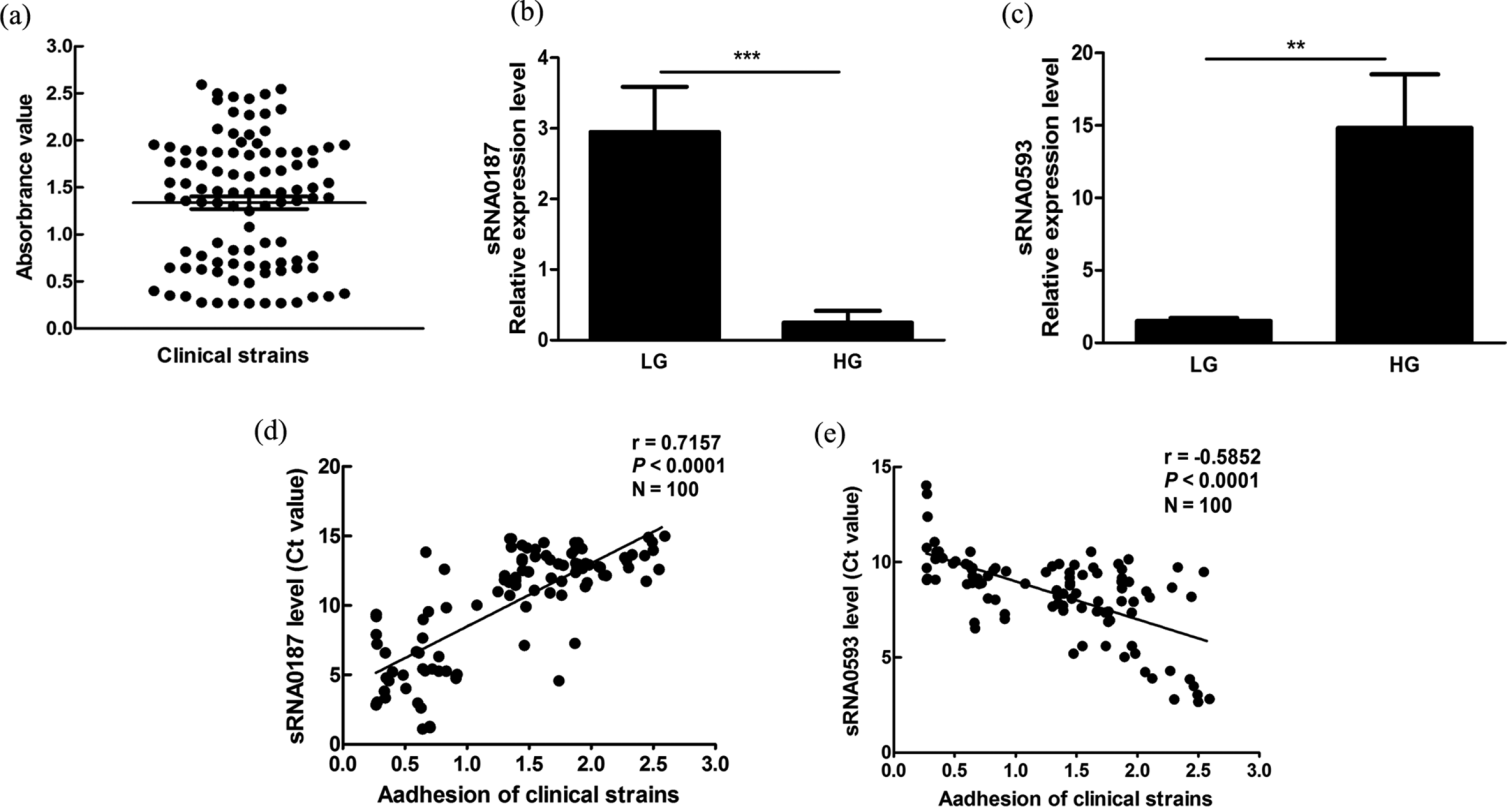
Differential expression analysis of sRNA0187 and sRNA0593 in 100 clinical strains with different adherence capacities. (a) Analysis of the adherence capacity of 100 clinical strains of *S. mutans.* An adherence assay was performed for each strain and repeated three times, and the mean value was used to represent the bacterial adherence capacity. (b, c) Relative expression levels of sRNA0187 and sRNA0593 in high-adherence strains and low-adherence strains. The error bars indicate the standard deviations of the strains. **P<0.01, ***P<0.001. (d, e) Spearman correlation analysis of sRNA087 and sRNA0593 expression (Ct value) with adherence among the 100 clinical isolates.

The ability of *S. mutans* clinical strains to bind to a smooth surface was evaluated using the crystal violet staining method. As shown in Fig. 6(a), the adherence of *S. mutans* clinical strains during biofilm formation for 4 h varied among the 100 strains. The median adherence value of the 100 *S. mutans* clinical strains was 1.443. The 100 clinical isolates were divided into two equal groups based on the median of adherence. The adherence values of the high-adherence group (HG) were higher than 1.443, and the low-adherence group (LG) adherence values were lower than 1.443.

We detected the expression of sRNA0187 and sRNA0593 in 50 HG strains and 50 LG strains by qRT-PCR. According to the data, sRNA0187 expression was higher in the LG than in the HG (*P*<0.05) (Fig. 6b), whereas the expression of sRNA0593 was lower in the LG than in the HG (*P*<0.05) (Fig. 6c). There was a fairly strong correlation between the Ct value of sRNA0187 and the adherence values of the *S. mutans* clinical strains (r=0.7157, *P<*0.0001) (Fig. 6d). Additionally, a moderate correlation was observed between the Ct value of sRNA0593 and the adherence values (r=0.5852, *P<*0.0001) (Fig. 6e).

## Discussion

A key element to the pathogenicity of *Streptococcus mutans* in the oral cavity is its capacity to bind rapidly to tooth surfaces to avoid being washed off by saliva [27]. Hence, studies on the adherence regulatory elements are especially necessary to obtain a more in-depth understanding of the cariogenic virulence factors in *S. mutans*. Although the adherence of *S. mutans* has been studied in some detail at the genetic level, a detailed understanding of the regulation of *S. mutans* adherence at the transcriptional level is lacking. The present study provides a significant advance in our understanding of adherence-related transcriptional factors by identifying the sRNA landscapes of *S. mutans* under different adhesion conditions during the initial stage of biofilm formation. Here, we first discovered many differentially expressed sRNAs, and then further analysis of the association between their expression profiles and adhesion in 100 clinical strains suggested a possible role for sRNAs in the process of adhesion.

Sucrose primarily serves as a substrate for the synthesis of EPSs to boost *S. mutans* adherence to the tooth surface [28]. Previous studies have demonstrated that sucrose provides the highest contribution to the highest biofilm mass of *S. mutans* compared with other carbohydrate [29]. To identify the sRNAs related to adherence, we constructed an adhesion model with sucrose. A previous study found that some bacteria adherence processes include an initial adherence (1 h) phase and a period resembling a lag phase (4 h) [30]. Therefore, we observed the dynamic changes of *S. mutans* during the initial adhesion (1–4 h) and found that 1 % sucrose concentrations and a 4 h incubation period were suitable for early biofilm formation, which included the adhesion process. We selected 0 and 1 % sucrose concentrations and a 4 h incubation period to identify adherence-related sRNAs in *S. mutans* UA159 and clinical strains. Biofilm cells demonstrate a different phenotype and gene expression pattern from planktonic cells [31, 32]. This study first focused on biofilm cells instead of planktonic cells to identify sRNAs and will be more meaningful for understanding the cariogenic activity of *S. mutans*.

This study revealed the expression of seven novel sRNAs in *S. mutans* and provided evidence for the relationship between sRNAs and adhesion in clinical strains, which is the first description, to the best of our knowledge, of adherence-related sRNA expression in *S. mutans* and clinical strains. We obtained information on all 763 identified sRNAs (provided in in Table S2), which should be a useful resource for studying *S. mutans* and its adhesion mechanism.

The expression levels of sRNAs are significantly influenced by environmental alterations, and some sRNAs are uniquely induced by specific conditions. Our 736 identified putative sRNAs did not match any previous sRNAs in any database, which indicated that these sRNAs might be specifically induced during the *S. mutans* adherence process, potentially acting to fine-tune adherence at the transcriptional level. These results are consistent with previous studies that showed that some sRNAs were uniquely stimulated under acidic conditions [15]. In *Ruegeria pomeroyi*, 14 sRNAs potentially involved in specific nutrient limitations were identified [33].

A large subset of sRNAs were only conserved among closely related species [34]. Some sRNAs show no sequence conservation to other related bacteria, which suggests that they may be species-specific, such as sRNA0329 and sRNA0330 in this study. Some sRNAs are not only conserved in strains of *S. mutans*, but also in other closely related bacteria, such as sRNA0187, sRNA0593 and sRNA0656 in this study, indicating potential roles in the genus *Streptococcus* [35]. sRNAs adjacent to coding regions are always conserved [36]. Our data also showed that sRNA0329 and sRNA0330 were adjacent to the coding regions of SMU_876, which suggested a potential relationship with the mRNA SMU_876. The expression of sRNA0187 and sRNA0593 were validated in all of the clinical strains, as well as in UA159, which indirectly suggested that these sRNAs were conserved [37] in *S. mutans*. However, although the sequences of some sRNAs are conserved in other bacteria, it is unclear whether these sRNAs are true sRNAs in other bacteria.

sRNAs modulate virulence and physiological functions, primarily by base-pairing with target RNAs or through protein binding. Direct RNA–RNA binding with a target mRNA is the typical mechanism [38, 39]. sRNAs simultaneously regulate multiple mRNA targets and change the polarity pattern within an operon [40], indicating that they may be involved in multiple functions. The sRNA RhyB regulates iron-associated functions by base-pairing with multiple targets in *Escherichia coli* [41, 42]. In the present study, the association between sRNA and the adhesion of clinical strains suggested a potential role of sRNAs in the process of adhesion, in which sRNA0187 probably regulates adherence negatively, while sRNA0593 might modulate it positively, and thus we wanted to determine whether their predicted target mRNAs were related to adhesion. Interestingly, multiple mRNAs involved in adhesion were the targets of sRNA0187 and sRNA0593, although the prediction scores of the target mRNAs were low. A previous review reported that a large number of true biological targets have relatively low prediction scores [43]. Nevertheless, the predicted potential targets of sRNAs are still useful for guiding future research and further enriching our knowledge of sRNAs.

The functions of sRNAs are more complex than those observed in coding regions. In our study, GO and KEGG pathway analysis also showed that the predicted targets of sRNAs were potentially involved in diverse biological processes in addition to adhesion. These results are consistent with a study by Stauffe that showed that sRNA GcvB was involved in multiple regulatory functions, including the transport of dipeptides, oligopeptides and amino acids in *E. coli* [44]. Because sRNAs have multiple regulatory functions, the complex regulatory roles of sRNA0187 and sRNA0593 in *S. mutans* functions other than adherence must be determined in future studies. In addition, we predicted the putative promoters of sRNA0187 and sRNA0593. Determining the promoters of sRNAs would help us understand the expression of sRNAs related to adhesion virulence in the *S. mutans* genome and would be beneficial for exploring the functions of sRNAs in subsequent studies.

### Conclusions

To conclude, we identified a total of 736 putative sRNAs through sequencing during the initial stage of biofilm formation. We performed validation experiments for 2 of the top 7 differentially expressed sRNAs in *S. mutans* UA159 and 100 clinical strains. Moreover, combined bioinformatics predictions and correlation analysis between the sRNAs and their adhesion capabilities led to a preliminary exploration of the potential functions of the sRNAs. The major aim of this study was to enrich the available information on the transcriptional regulation on adhesion in *S. mutans* as the principal cariogenic bacteria. A better understanding of sRNAs as transcriptional factors will help to provide new insights into development of strategies for the prevention and treatment of dental caries in the future.

## Supplementary Data

571 Supplementary File 1
