# Supplementary material for: High-throughput sequencing identification and characterization of potentially adhesion-related small RNAs in Streptococcus mutans
Source: J Med Microbiol. 2018 Mar 29;67(5):641–51. doi: 10.1099/jmm.0.000718 (PMC5994696; doi:10.1099/jmm.0.000718)
Supplement: Supplementary File 1 [file jmm-67-641-s001.pdf]

Supplementary Fig.S1: The secondary structure of sRNAs predicted by RNAfold. △G present their MFE of the thermodynamic ensemble.

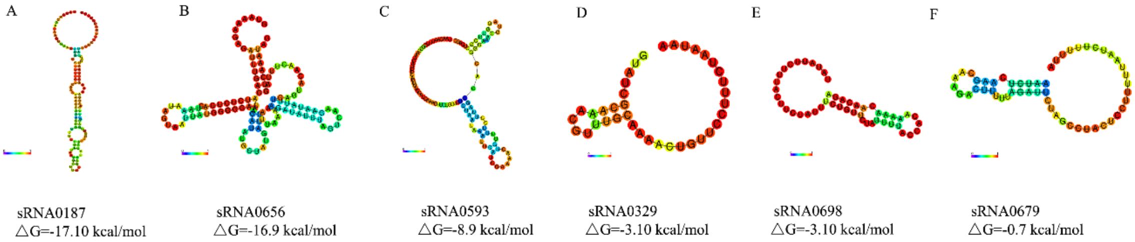

Supplementary Fig.S2: Multiple sequence alignment of putative homologues to confirmed sRNA0656, sRNA0679, sRNA0698, sRNA0329 and sRNA0330 in *S. mutans*.

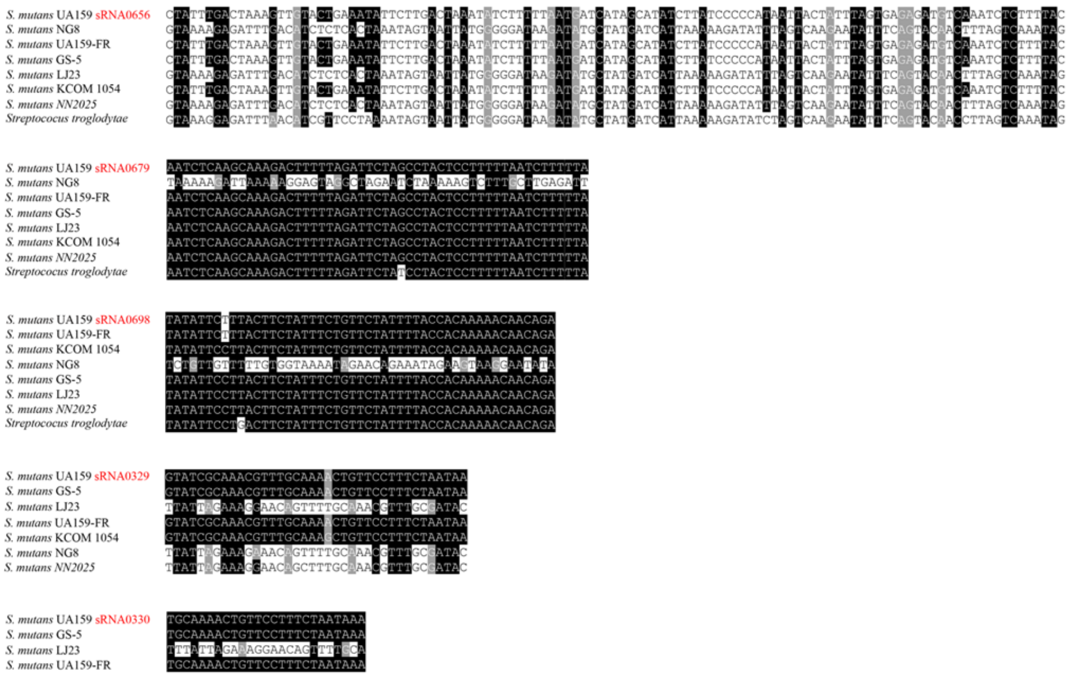

Supplement Table 1. sRNA primer sequences used in this study.

sRNA ID	Sequence	Primer
Srn0593	TAACAATTTCGCCAATCATTTCATTTTCCATCAAACTTGTCCTTTCTAATAAATTCATCCAAAACTGTTTTCCTTAGGCACGGGAAACGTAGGTTCCCTCAGC	**F: CGCCAATCATTTCATTTTCCA**<br>**R: CCTACGTTTCCCGTGCCTAA**
Srn0330	TTTATTAGAAAGGAACAGTTTTGCA	**F: TTTATTAGAAAGGAACAGTTTTG**<br>**R: universal primer**
Srn0329	GTATCGCAAACGTTTGCAAAACTGTTCCTTTCTAATAA	**F: GCAAAACTGTTCCTTTCTAATAA**<br>**R: universal primer**
Srn0187	TATCACGATAACTGTACATGCGTTCCGTCAAATAACCAAAGTGTTTTTGAGAATTCTTTTGAACATCATTTGTGTACTTTTGAGTTAAAAGCGGAATTACCATTCTCCTTCTCCTTTT	**F: CGTTCCGTCAAATAACCAAAGTG**<br>**R: AAGGAGAATGGTAATTCCGCTTT**
Srn0679	AATCTCAAGCAAAGACTTTTTAGATTCTAGCCTACTCCTTTTTAATCTTTTTA	**F: AATCTCAAGCAAAGACTTTTTAGA**<br>**R: universal primer**
Srn0698	TATATTCTTTACTTCTATTTCTGTTCTATTTTACCACAAAAACAACAGA	**F: CTATTTCTGTTCTATTTTACCACA**<br>**R: universal primer**
Srn0656	GTAAAAGAGATTTGACATCTCTCACTAAATAGTAATTATGGGGGATAAGATATGCTATGATCATTAAAAAGATATTTAGTCAAGAATATTTCAGTACAACTTTAGTCAAATAG	**F:TATGGGGGATAAGATATGCTATGAT**<br>**R: universal primer**

The universal primer was commercialized supplied with the Mir-X miRNA qRT-PCR SYBR kits (TaKaRa, Tokyo, Japan).

Supplement Table 2. The information of potential candidate sRNAs.

	All-unitags	Length	Expression (All)	Expression(control_2)	Expression(scr_2)	Expression(scr_1)	Expression (control_1)	Expression(scr_3)	Expression(control_3)	Chromosome	Begin	End	Strand	Pre-gene	Gap-1	Next-gene	Gap-2	Direction	Description	Sequence
>sRNA0001	s0000047	120	429	58	103	69	85	68	46	NC_004350.2	14807	14926	+	SMU_15	155	SMU_16	214	/+/+/+/	IGR	AAAACCAAATCCCCTTTAATTTTGTTGTAATTTATCTGATGTTATAGAAAGATAAAATTTTAAGATTCTAGGAGGGTTCCTTTTTTGACACAATCCACGCCCGTGTTTAATTAAGATTTG
>sRNA0002	s0000048	113	67	10	13	18	13	7	6	NC_004350.2	14918	15030	+	SMU_15	266	SMU_16	110	/+/+/+/	IGR	TAAGATTTGAAATTTTAAACTCAAATATTCCGAATGGTAGAAAGTTTCATTCAAGGTTTGAGTCGCTCGCCAATGCCAATAAAAGAAATAATCTTGAATTTGCAGGGTTTAAA
>sRNA0003	s0000057	48	105	22	14	26	21	2	20	NC_004350.2	16873	16920	-	SMU_18	112	SMU_r01	-43	/+/-/+/	AM	TTAGGCACGCCGCCAGCGTTCGTCCTGAGCCAGGATCAAACTCTCATT
>sRNA0004	s0000060	118	166	31	34	41	27	13	20	NC_004350.2	18559	18676	-	SMU_t01	1	SMU_r02	141	/+/-/+/	IGR	TAGACTTCCTTGTCTTAAACAAGATATGAAGTTGAACTCCAGACTGACTTCTTAGAAAAATAGATAATCTTCTACAGAAGTATTCGCAAGCGAACCGTCTGTTAGTATCCTGTTTTAA
>sRNA0005	s0000071	79	436	92	51	76	72	52	93	NC_004350.2	22051	22129	-	SMU_t02	-39	SMU_t03	-9	/+/-/+/	AM	CCACGGGACCTCTAACTAATCACTTTAGTTACTTAGTTATGGGAGTTAACGGGCTCGAACCGCTGACCCTCTGCTTGTA
>sRNA0006	s0000072	82	825	138	126	115	151	113	182	NC_004350.2	22176	22257	-	SMU_t03	-16	SMU_t04	-37	/+/-/+/	AM	TTAAAAGTCAAATGCTCTACCAACTGAGCTAACGAGTCGATGATCCCTCTCTTTTCAGAAAAAGACGGTCCCGACGGGAATC
>sRNA0007	s0000075	59	1581	278	302	218	350	183	250	NC_004350.2	22447	22505	-	SMU_t06	-23	SMU_t07	-32	/+/-/+/	AM	CAAGGTGATGTTCTACCACTGAACTACGTTCGCAATGCCGGCTACATGACTTGAACACG
>sRNA0008	s0000076	43	73	18	13	11	14	7	10	NC_004350.2	22527	22569	-	SMU_t07	-17	SMU_t08	-17	/+/-/+/	AM	CAGTTCCGCCACCCCGGCCTCCTAAAGCGAACGACGGGATTCG
>sRNA0009	s0000077	50	82	18	12	10	19	11	12	NC_004350.2	22610	22659	-	SMU_t08	-27	SMU_t09	-8	/+/-/+/	AM	CAAGGGTGCTCCTTCTTATTTATGCCGAGGACCGGAATCGAACCGGTACG
>sRNA0010	s0000078	52	169	23	42	39	28	10	27	NC_004350.2	22705	22756	-	SMU_t09	-19	SMU_t10	22	/+/-/+/	AM	AAAGTGTGCTAGTCCTTTACTAACCCCACTCATGCACCCTGCAGGATTCGAA
>sRNA0011	s0000113	114	232	44	37	41	40	23	47	NC_004350.2	30613	30726	-	SMU_26	-72	SMU_27	-39	/+/-/+/	AM	TGAGATTAGAAATCCTGTCAAAAATCTCTTCATTAGTCATCTTAGTCCCCCCTGTTTGAAAATTCTTCTACTAATTGACCAACAACATCAGTTTCAAGCATGATTCGGATTTGC
>sRNA0012	s0000135	47	85	15	10	19	13	10	18	NC_004350.2	38582	38628	-	SMU_32	166	SMU_33	5	/+/-/+/	IGR	TGAGCACCTGACATTCCAGATAACCAAGTAAAAATTGTGGAGATACC
>sRNA0013	s0000147	77	306	60	56	89	38	25	38	NC_004350.2	40551	40627	-	SMU_35	15	SMU_36	16	/+/-/+/	IGR	TAACGCCCCTACTCATGATTTACCAACCCTGATAGAAATAGTTTATGAACAGGCCTTTACCTTTACCATAAAATCAT
>sRNA0014	s0000154	86	303	55	53	64	36	39	56	NC_004350.2	42944	43029	+	SMU_37	5	SMU_38c	93	/+/+/-/	IGR	TATTTAAAAATTAGCTCATTATTAACTGTAATGAATGACTTATCGCTAACAACGAGAGGAGATGCAAGTCGTCTTCTCTTTTTGTA
>sRNA0015	s0000163	132	198	29	32	48	22	43	24	NC_004350.2	44370	44501	+	SMU_39	29	SMU_40	17	/+/+/+/	IGR	CTTTCTGAAAGAAAAGTTTAAGGAGTGATTAAGAAAGATCTTATATACTATAGTCATCCTAGCAATAGGAATCCTAAAACTTTTCTTTTTCATAAATCTCCTAAAAAGCCCGCTTCAAGCGGGTTTTTCATT
>sRNA0016	s0000178	133	196	17	61	46	38	15	19	NC_004350.2	47848	47980	-	SMU_44	66	SMU_45	-11	/+/-/+/	AM	TAAGACTAACATATCACTGTCGTGTGAAAAAGCAAAAGAAATTTCTGCATCTGTTGAAACATCAGCTAAAGACGGCTCTATTTCTTCTTCTTCTTCAAAATAGTCTAATATAATTTTAAATTCGCCTGATTTT
>sRNA0017	s0000197	28	171	29	38	17	28	26	33	NC_004350.2	53531	53558	-	SMU_49	-8	SMU_50	304	/+/-/+/	AM	AATAAACGGAATCTTTTGCTCAAATATT
>sRNA0018	s0000198	98	78	6	12	11	16	16	17	NC_004350.2	53594	53691	-	SMU_49	55	SMU_50	171	/+/-/+/	IGR	TCAGACTCATCTTTTTGCCCAGTGTTGTTGTCGTAGGCGTGTTTAATTCCTTTCGTTTTTTATTTTATAGCGACTTTTTGTCGCTTGCAAAATTTTCA
>sRNA0019	s0000202	93	199	25	54	34	38	18	30	NC_004350.2	53747	53839	-	SMU_49	208	SMU_50	23	/+/-/+/	IGR	GCGACCTTCAACAAAATGGGCGGTAAATCAGAAAACTTTGATTTCGCTGACGCACCCCATCAGGACGACTAGGACTTTCTTAGCCTACTGGCG
>sRNA0020	s0000220	86	128	8	26	31	24	15	24	NC_004350.2	57513	57598	-	SMU_56	-23	SMU_58	41	/+/-/+/	AM	GATAATGATCTAGTGTGTTCAAATCTGTATCCAGCGGGACATCATAGATAACTAGATAGTTTTCATGATATTGCAACTAGCTCTTC
>sRNA0021	s0000235	93	145	26	27	29	20	15	28	NC_004350.2	59165	59257	-	SMU_58	231	SMU_59	-13	/+/-/+/	AM	TAACGGTTGATCATTGTTAAGATATTAAGGGCGTTAAGCGGACACAGTCAAAATAGGAGTTTCGACGAAGAAGCGACTGCTTCTAGGAAAAAC
>sRNA0022	s0000241	134	289	45	45	48	64	47	40	NC_004350.2	61247	61380	-	SMU_60	-1	SMU_61	251	/+/-/+/	AM	AAGCGACCGCAAGGCCGCAATAAAAGAATTTATGAATACAGGCTCTTAAAAACGACTGGCTAGAAAATTTCAGCTTATCCTACTTGCCCAGCGGTTGTTTTTCGTGTGATATAAAATATGATAAGCGGCTTATC
>sRNA0023	s0000243	110	69	12	4	18	15	8	12	NC_004350.2	61420	61529	+	SMU_60	172	SMU_61	102	/+/+/+/	IGR	TAAGTATAGGTTCTAAGTATAAAGAATAAGAAAACAAAGATTAATCGTTTGCTGCAAGACTACGCTTATGTCTTATGCTTCCTTTTTGAAACTCGCTGAATGTGTTCCCA
>sRNA0024	s0000244	80	73	19	9	10	15	11	9	NC_004350.2	61505	61584	-	SMU_60	257	SMU_61	47	/+/-/+/	IGR	TAACTCTATAATATAGTAAATTTACTAGACTTCTATATATTTTTCTGACATCTTTTGGGAACACATTCAGCGAGTTTCAA
>sRNA0025	s0000246	124	1746	320	304	356	268	261	237	NC_004350.2	61549	61672	-	SMU_60	301	SMU_61	-41	/+/-/+/	AM	TAACCTCAGGCTCTTAATTTTCTTCCCAAAATCTTTTAACATAAAACCTTTTCCTATAATCTCTGTCTAAACTTTTACTATTTTATCATAACTCTATAATATAGTAAATTTACTAGACTTCTAT
>sRNA0026	s0000259	126	67	9	16	21	8	5	8	NC_004350.2	64838	64963	+	SMU_63c	-82	SMU_64	254	/-/+/+/	AM	AGACCTTTGGAGGATTTACGTTTGACATCATGTCCCTTAGCATTTGAGAAAACAGAGCGATTAAAAAGAAATTTTTTACTCATTATAGTTTCTAACCCTTCTAAAAAGTATGAATAGCAGTTTTCT
>sRNA0027	s0000274	131	301	60	44	70	42	45	40	NC_004350.2	68961	69091	-	SMU_67	12	SMU_68	124	/+/-/+/	IGR	TAACTTAAAAGCACCTATAAATCTAAAATCAATTTCGTCGATCCAATCAGCACAAGACACTCAAAACAACGAAAAAAATCTCAACTTTCTTCTTTATCTCGCGCTTCGATTTAAGTCAAGACCTCCCCTCT
>sRNA0028	s0000281	60	115	22	13	32	20	10	18	NC_004350.2	69432	69491	-	SMU_68	140	SMU_70	140	/+/-/+/	IGR	TGTCTCGCCTTTCGCAAATTGGATTAGGTATCAACATGATCGGACGTTCATGGGATTAGA
>sRNA0029	s0000282	37	65	6	24	8	12	10	5	NC_004350.2	69434	69470	+	SMU_68	142	SMU_70	161	/+/+/+/	IGR	TAATCCCATGAACGTCCGATCATGTTGATACCTAATC
>sRNA0030	s0000287	28	105	9	20	29	25	13	9	NC_004350.2	69612	69639	-	SMU_68	320	SMU_70	-8	/+/-/+/	AM	TAATGTCATGATTCATCCTCTTTTTATA
>sRNA0031	s0000294	70	422	53	89	88	91	59	42	NC_004350.2	71368	71437	-	SMU_70	253	SMU_71	86	/+/-/+/	IGR	CTTCCTTCCTATAAATGAGAGGATAGGACAGACTTCTATGATTGCTGTGTAACTATCCATAAAATAGTGT
>sRNA0032	s0000309	147	64	15	12	14	13	6	4	NC_004350.2	73409	73555	-	SMU_72	-88	SMU_73	-38	/+/-/+/	AM	AATCATTGCAATCGTTTCGGTAACCTGTTTAATATCCATCAGACTAGAACTCCTATTCTTACAAGTTATGCATAGCGTCAAAAATAGCTGAACTTTGAATGTTAATTTTGACATTCAAGGTCTCACCGAAAGCTTCAAACTCTTTCC
>sRNA0033	s0000312	39	170	24	29	36	21	25	35	NC_004350.2	74837	74875	-	SMU_73	-17	SMU_74	52	/+/-/+/	AM	AAAGACTTGCAAGTGCTCTACTTAATTTTTAAAGCTATG
>sRNA0034	s0000357	145	81	14	14	12	13	9	19	NC_004350.2	87513	87657	-	SMU_82	33	SMU_83	351	/+/-/+/	IGR	TCATGACGTGAGATTTTCGAAATGATACTTTTCGAAAATCAGGCTAGTAAGACGTTTGGGACATTCGCCAAATAACAATTGCTGTAGAGAAAGACAATCACTTTAGTAATTAGTCATTCTTAACTACCTCACTCTCCTGATTTTC
>sRNA0035	s0000359	110	94	14	28	16	19	7	10	NC_004350.2	87623	87732	-	SMU_82	143	SMU_83	276	/+/-/+/	IGR	AATTCCTTTTTACATCAGCCATATTCCAAAAACAGAGGCTGGTTTGCAACCTCTGAATTTAATTTGGGCTTATAGTCATGACGTGAGATTTTCGAAATGATACTTTTCGA
>sRNA0036	s0000361	90	74	13	22	8	16	9	6	NC_004350.2	87733	87822	-	SMU_82	253	SMU_83	186	/+/-/+/	IGR	TATTTCTAGGAATTGAAGATCGGGTTCAGTTCCTTTCTTTGAAACTTAGAAGGTTGTAATATTTTGAGGACTTGTGCTGATCAACTCAGC
>sRNA0037	s0000384	40	89	14	11	10	24	13	17	NC_004350.2	92033	92072	-	SMU_87	25	SMU_88c	107	/+/-/-/	IGR	CATTAGAATGATTTCTAATGAGGGCTTTATGCTTATTCTT
>sRNA0038	s0000386	101	104	13	15	36	16	14	10	NC_004350.2	92069	92169	+	SMU_87	61	SMU_88c	10	/+/+/-/	IGR	AATGAGGGCTTTTAGCGTTTGTGGTCTGTTTTGGAAATTATTATCAACGGTAGTGGTAGGCGTTTGACCTACATTTATTTTCTAATTGTTGATTTGTGATA
>sRNA0039	s0000393	116	187	32	21	51	20	33	30	NC_004350.2	93175	93290	+	SMU_88c	139	SMU_89c	-36	/-/+/-/	AM	TCAAAGCAGATTAATAAAAACAATAACACCTTTGAAAGCCATGAATCAAGTTATTATGCCTACTGGCTTATTTAATAGGTTATTGTTCTTTATGCGGCGTTTTAGTCTGATTAAGC
>sRNA0040	s0000414	92	91	10	18	26	11	17	9	NC_004350.2	96105	96196	+	SMU_93c	15	SMU_94c	95	/-/+/-/	IGR	ATTGCGAGCTGGCGAACAAACGGCTAGCGGAAAGCTATCCATGATGACGATATCGTCCTAATCAAATGGCCCCGTCATGCCTTGATAAATTC
>sRNA0041	s0000423	51	599	103	116	89	115	85	91	NC_004350.2	96537	96587	-	SMU_94c	76	SMU_96	595	/-/-/+/	IGR	GCTACCGTCCGTATCACCTAAGGAAAGTCTCAAAAGAAGACTTTTTCTACA
>sRNA0042	s0000425	22	404	66	56	84	87	67	44	NC_004350.2	96592	96613	-	SMU_94c	131	SMU_96	569	/-/-/+/	IGR	TTAGGTATGGAATTCCGAAGGA
>sRNA0043	s0000428	79	860	127	178	130	161	137	127	NC_004350.2	96697	96775	+	SMU_94c	236	SMU_96	407	/-/+/+/	IGR	TAAAGCTCGCTTCGCTCGCAAATAAGAAAAATTTAAATTGAAATTTGCAGAGCTCAAAATTTTTTTACGTTGGAAAATA
>sRNA0044	s0000435	102	218	29	25	56	34	28	46	NC_004350.2	97012	97113	-	SMU_94c	551	SMU_96	69	/-/-/+/	IGR	CTTGTCCAGCAAATACTTCTAATTCCAAAGTGGTTCTCCTTATCATAATCGGTCAAGGCTAAATCCCCACAATAATCCGATTATCTATAATTATCGTTCCTT
>sRNA0045	s0000437	46	557	94	66	105	63	120	109	NC_004350.2	97112	97157	-	SMU_94c	651	SMU_96	25	/-/-/+/	IGR	TAAAATGGCACGAGCAACTTCAATCATTGACAATTCACTTTTTTCT
>sRNA0046	s0000438	43	774	133	75	125	164	102	175	NC_004350.2	97148	97190	-	SMU_94c	687	SMU_96	-8	/-/-/+/	AM	AAAATACATTTCTTTATCTCGGCCACGTTCTTCTAAAATGGCA
>sRNA0047	s0000441	127	153	14	36	29	33	13	28	NC_004350.2	97599	97725	-	SMU_96	-71	SMU_97	79	/+/-/+/	AM	AGAGGTGCCCAATAAATAATATAAACTATCTTATGAATTTTGTCAATGAAGAAATTTATTCTCCTTCTTCATCTTCCTCATCATCAGAATAATCGTCATCTTCTTCATCTAAATCAACATTTTCGTC
>sRNA0048	s0000442	85	408	75	68	93	49	55	68	NC_004350.2	97699	97783	-	SMU_96	29	SMU_97	21	/+/-/+/	IGR	ATTGTGAGGAAAATGAAAAAATGCCCCCTACAAGTAGAGAGCTTATTCTGACCTCAAAAGAGGTGCCCAATAAATAATATAAACT
>sRNA0049	s0000448	145	99	13	13	22	18	10	23	NC_004350.2	99534	99678	+	SMU_97	120	SMU_t19	654	/+/+/+/	IGR	AAGGTGTTTCTTTTTTTGGATCATTATTACAGCTAATCGTTCAATTCAAAAATGAATAAATCATATTAGCTATTACAAAAATACATTCCAGAATAAAAATTCCAAAACAGAGAAGCTTGACAGTACCGCTTCGGCTATACTTAGA
>sRNA0050	s0000463	148	271	56	60	40	35	32	48	NC_004350.2	101351	1E+05	-	SMU_99	-104	SMU_100	282	/+/-/+/	AM	TCAGTCTAGGGAAAGAGAATCTTTCAATTTGTTAACCAAGTAATTAAGCTTTACCTTCTGAACCAAAGACATCAATACGTTCTTCAACCGCTTCTGTAATAGCTTCAAAACCTGGTTTCAAGAATTTACGTGGGTCAAAGAGTTTTTT
>sRNA0051	s0000470	63	72	11	13	13	17	11	7	NC_004350.2	101765	1E+05	-	SMU_99	310	SMU_100	-47	/+/-/+/	AM	AATTCCATGGATTAATCTTTGATCTACTCTAGCCTCAACAACTGTCATAATAAATTCCTCCTT
>sRNA0052	s0000480	142	423	82	80	87	49	67	58	NC_004350.2	106450	1E+05	-	SMU_104	-50	SMU_105	-90	/+/-/+/	AM	GAATAGTAATACCATTGATATAGTTTGATACAGTTCCTCGACTGACATTAGCCAGTTGAGCTACATCATTGATTGTAGGATTTCTTTTCATTTATTTATTATAAGATTCTCTAATATAAGCTATCTCACTTATACTATTGCG
>sRNA0053	s0000484	136	315	52	42	76	56	50	39	NC_004350.2	107476	1E+05	-	SMU_105	-23	SMU_106c	123	/+/-/-/	AM	TAGGATTTTTTTACTAGCACCACGGGTATTACCTATATAAAATAAGCATAAATCCTCTGTAAACTATTTCGTTCACTTGACTATTTTATTCCCGGTTTATCTATTACCAGATTTACACTAACCTTTCAGGCTCAAC
>sRNA0054	s0000491	97	325	51	66	74	49	39	46	NC_004350.2	108470	1E+05	-	SMU_106c	500	SMU_t20	-8	/-/-/+/	AM	TAAATCGGCTAACATTAGTATTTTACTATTTCTAAAAATTTTGTCAACAAAAATAAACAACAAAGATTTCAGAATATTCTGGTCTCGATCAGCATCG
>sRNA0055	s0000492	123	723	122	146	118	145	93	99	NC_004350.2	108553	1E+05	-	SMU_106c	583	SMU_t20	-117	/-/-/+/	AM	TTAGCGAAAAAGCCCAATTCAAGGGCTTTCTATCTTGTATTTTTATGCCGATTGCAGGACTTGAACCTGTGACCTACGCGTTACGAGTGCGTTGCTCTACCAGCTGAGCTAAATCGGCTAACA
>sRNA0056	s0000494	91	172	17	23	39	35	32	26	NC_004350.2	108689	1E+05	-	SMU_t20	59	SMU_107	329	/+/-/+/	IGR	TCTATGTTGGTAGTTGTTTATGGGTATACCCTATAAGAAAAAATCGAGCAAGAATTTAAAGTTGTTAGAACTTTACTATGATTTCCCAACA
>sRNA0057	s0000502	135	133	31	4	19	22	21	36	NC_004350.2	109406	1E+05	-	SMU_108	-11	SMU_109	42	/+/-/+/	AM	ATTATGCAATGATAATAAGACCTTGTTCTGCTGCCATAGTTCTTCACCTCCTTCAAATAGACTTTTCCTAATGTTACGTAATAATGATGAGACCTTGTTCCGTAGTCATATGCTCATTCCCTTTCATTTATTGTT
>sRNA0058	s0000531	117	352	60	74	70	47	51	50	NC_004350.2	118423	1E+05	-	SMU_117c	333	SMU_118c	166	/-/-/-/	IGR	AAACTGAAGATTTTGAAAATCCTCTCAATATCATTTCTAACAATTAAACTTCTTTTTATTACAGCGGAAACGGTTCTTTTATTGAGGCTGGAACAAAAATGTTCCAACCTTTTTATT
>sRNA0059	s0000542	130	182	17	34	38	40	27	26	NC_004350.2	120785	1E+05	-	SMU_119	109	SMU_120	189	/-/-/+/	IGR	AAAGGTCAGTTTATGGAACTTTGAATGATTTCTTATGCCATAACATGGCCTGATTTTTAATAGACTTGTTGGGAAACTAACTATTTTCTTTAAGCAATGGGGGCGGGCTTTTTCAAATCGTTACTTGAAA
>sRNA0060	s0000545	65	560	92	110	108	99	71	80	NC_004350.2	121046	1E+05	-	SMU_119	370	SMU_120	-7	/-/-/+/	AM	TTAGCCATTTAACTGTTTTCCTCCTACTTAGTTTAAGGTGAATTCACCACATACTTTAGCATTAT
>sRNA0061	s0000607	62	82	24	12	14	8	15	9	NC_004350.2	140498	1E+05	+	SMU_135	134	SMU_136c	-5	/-/+/-/	AM	AAAGTCAACTTCCAAGTGGTTTAAAAGTAACAAGATAGTGGTTTTTTCTTTTGCTCTTAATC
>sRNA0062	s0000635	42	264	41	54	48	48	26	47	NC_004350.2	147174	1E+05	+	SMU_141	43	SMU_143c	80	/+/+/-/	IGR	TAATGTAATCATATGTGGATGAGCTGGCTTTGTTGACAGTCA
>sRNA0063	s0000653	135	202	36	19	32	36	29	50	NC_004350.2	152696	2E+05	-	SMU_148	-93	SMU_149	169	/+/-/+/	AM	AAAGCCCGCATAAGCGGGCCTGACTGCTAAACGATATCAACTTAGAACTGATCAATCAAGAGCTGCTTCATCTCTTCAATGTAAGGCTGACGTGGATTAGCTGGTGTACATTGATCATCATAAGCCAAGCTTGCC

>sRNA0064	s0000663	101	440	81	59	73	69	68	90	NC_004350.2	153473	2E+05	+	SMU_149	172	SMU_150	240	/+/+/+/	IGR	GAAGAGCTATTTCGGTGGGCCTTTGGCCCTGACCGGTAGAAGTGGCTTACAGTCGCAGAACAAACCACCAGTTCACACTGGTGGTTTTGATTGTTAGCCTA
>sRNA0065	s0000665	26	615	83	124	117	103	107	81	NC_004350.2	153564	2E+05	-	SMU_149	263	SMU_150	224	/+/-/+/	IGR	CTTTGGGAAAATAAGATAGGCTAACA
>sRNA0066	s0000666	48	813	109	161	215	94	135	99	NC_004350.2	153565	2E+05	+	SMU_149	264	SMU_150	201	/+/+/+/	IGR	GTTAGCCTATCTTATTTTCCCAAAGCTTGTGTAAAACTTCTATTAAAC
>sRNA0067	s0000674	118	129	26	19	40	10	22	12	NC_004350.2	154239	2E+05	-	SMU_151	-22	SMU_152	100	/+/-/+/	AM	TAAATAAAAAATTCTATTGGACGAAAAATGGACAATGCCACTGTACCCATTCAGAAATCTCGCCCTGAATTGACATTGCCCACCATTATATCAATTTAGTGTGGAAAAACTACAGATC
>sRNA0068	s0000683	82	290	46	43	82	26	60	33	NC_004350.2	155105	2E+05	-	SMU_152	230	SMU_153	182	/+/-/+/	IGR	TATATAAGTCTTGAGACGTACCACCACTGCTACTAACAACAAAGCTGCACTGAAAATGATAATAATGAGTTGTAACGATTCA
>sRNA0069	s0000687	54	244	22	36	78	35	38	35	NC_004350.2	155615	2E+05	-	SMU_153	146	SMU_154	544	/+/-/+/	IGR	TAATCAAACCATCCCTCTCTAATTCACTTAACTTCCTGTCTACTTTTTCTTGAC
>sRNA0070	s0000688	146	371	43	49	105	31	88	55	NC_004350.2	155649	2E+05	-	SMU_153	180	SMU_154	418	/+/-/+/	IGR	TTTGACGCCGCCTGATATTGTCCTCTCTGCAATGGCACACGACATCAATAATCGTATTAGCAGTACGGCCATAGTCTGTTATAGAATAGACGACCTTAGGAGGCATCTGCTGATAATCTCTACGACTAATCAAACCATCCCTCTCT
>sRNA0071	s0000689	103	162	20	19	44	19	40	20	NC_004350.2	155751	2E+05	-	SMU_153	282	SMU_154	359	/+/-/+/	IGR	TAAATGAACAATCACTTCTTAAAAATCGTACGAAGTTAAGTCAATTTCTTCGCCTAATATTTGACGCCGCCTGATATTGTCCTCTCTGCAATGGCACACGACA
>sRNA0072	s0000693	33	70	3	11	21	3	19	13	NC_004350.2	155891	2E+05	-	SMU_153	422	SMU_154	289	/+/-/+/	IGR	GTTACATATGAACATAGACCTTGATATAAATCG
>sRNA0073	s0000694	35	88	11	14	30	7	14	12	NC_004350.2	155893	2E+05	+	SMU_153	424	SMU_154	285	/+/+/+/	IGR	ATTTATATCAAGGTCTATGTTCATATGTAACGCAG
>sRNA0074	s0000699	32	81	6	12	27	4	23	9	NC_004350.2	155985	2E+05	-	SMU_153	516	SMU_154	196	/+/-/+/	IGR	AAACGGCTACTGGAATAATGTTTAATTTTACT
>sRNA0075	s0000708	53	111	27	17	15	20	18	14	NC_004350.2	156521	2E+05	-	SMU_154	40	SMU_155	655	/+/-/+/	IGR	TGACTTGCTACAGCTAAAAAAGCCATCCTAATGGACAGCTTTTTTATTTAATC
>sRNA0076	s0000718	91	166	34	28	35	28	24	17	NC_004350.2	157036	2E+05	-	SMU_154	555	SMU_155	102	/+/-/+/	IGR	ATTGCTTTTCTCGTCAACGATATTGCTAGGCAAAAGTTCAACAATCCCAGCGATAACTGACAACAGTACAGCTCTATCTTACTCCCTTAAC
>sRNA0077	s0000719	78	83	18	13	12	14	11	15	NC_004350.2	157137	2E+05	-	SMU_154	656	SMU_155	14	/+/-/+/	IGR	AAAATTAGTCTACGTGTTTTCTACAAAGTTTAAGAAATAAGCCTTATCACTCTAACGTCAATGTATCAAAAATACAGC
>sRNA0078	s0000725	136	1281	247	223	218	237	159	197	NC_004350.2	160693	2E+05	-	SMU_157	-85	SMU_158	24	/+/-/+/	AM	TTAGGAGTCTTTTTTCTTTGGTAAAAAGTACAACCAGCCAAGTAAGACTATCACAGCCCGGACGAGTGAAAACTAGCTTCCTTAGCTTCTTCTAGTTTTTTGAGGTAATATTCACGGTTCTCTTCGATTTCATGGA
>sRNA0079	s0000733	24	136	16	22	37	25	18	18	NC_004350.2	162747	2E+05	-	SMU_159	140	SMU_160	16	/+/-/+/	IGR	TACTGACAGTATAAAGGATAACGT
>sRNA0080	s0000734	75	164	36	18	23	34	38	15	NC_004350.2	162758	2E+05	-	SMU_159	151	SMU_160	-46	/+/-/+/	AM	AGCAACTCACGGTAAATTTTATCTGAGTGAATGATACGAATGGTCATTGTGTTTTCCTCCTATACTGACAGTATA
>sRNA0081	s0000735	148	312	66	57	53	54	45	37	NC_004350.2	163595	2E+05	-	SMU_160	-96	SMU_161	-72	/+/-/+/	AM	CGTAATAATGCAATGTTCGCTTGGTTAGACCAGTAATGGTCATGATTTCTTTTAGTGTGTAGTCTCTGTCCAAAATTCATCCGCCTCCGCTAAGATTTCTGAAGCTGGCAAAAACGTTGCTTCTTCAATTGTTTGACCCGTTTTTTCT
>sRNA0082	s0000759	98	99	24	2	23	21	17	12	NC_004350.2	169595	2E+05	+	SMU_168	190	SMU_169	106	/+/+/+/	IGR	AAAGTGAAGCAGAACTTACTTTAATAATGACAAGAGAGAAAAATTTATAATTATCAGCCTGTAAGTCTTGACTTTGGGGCTGATTTTTTGATATTATA
>sRNA0083	s0000760	39	76	12	8	17	14	16	9	NC_004350.2	169632	2E+05	-	SMU_168	227	SMU_169	128	/+/-/+/	IGR	CAAAGTCAAGACTTACAGGCTGATAATTATAAATTTTTC
>sRNA0084	s0000768	46	214	41	23	52	44	28	26	NC_004350.2	170649	2E+05	-	SMU_170	-12	SMU_172	289	/+/-/+/	AM	GAAGTGTTTGAAAATGCTGTAATGTAGCTGTTCTTAACGTTTTGAG
>sRNA0085	s0000782	67	161	26	34	38	25	10	28	NC_004350.2	171611	2E+05	+	SMU_173	57	SMU_174c	75	/+/+/-/	IGR	TAAATGCATTATTAAGACTTGCTATAACACTAAACAGTTATACCTCGCTTTGAATTATCAGTCTTCT
>sRNA0086	s0000784	145	249	46	53	43	45	29	33	NC_004350.2	171649	2E+05	+	SMU_173	95	SMU_174c	-41	/+/+/-/	AM	TATACCTCGCTTTGAATTATCAGTCTTCTTTAGGGTCTGAAAAGATTACTAATTCATTCTCTTAACTTTCTGTCTAACCTTCGGAAGCGAAAGAACAGTAGAGTCATCCTATGGAATCATGTAGCAATGGTGTTAATTCATTGAG
>sRNA0087	s0000810	129	79	13	9	13	26	5	13	NC_004350.2	180491	2E+05	-	SMU_183	-105	SMU_183	-26	/+/-/+/	AM	TAAGCCTTGTATAAATTCTGTAATCATGAAGCATCACCTGCCATTAAAAATTGTGATTGATAGGTTTTAGTCAGATTTTTCTGTGTAAAAACAGTTTCTGTCTGACCGAAGGCAAGGACTTTTTTGTTC
>sRNA0088	s0000057	48	105	22	14	26	21	2	20	NC_004350.2	16873	16920	-	SMU_18	112	SMU_r01	-43	/+/-/+/	AM	TTAGGCACGCCGCCAGCGTTCGTCCTGAGCCAGGATCAAACTCTCATT
>sRNA0089	s0000060	118	166	31	34	41	27	13	20	NC_004350.2	18559	18676	-	SMU_t01	1	SMU_r02	141	/+/-/+/	IGR	TAGACTTCCTTGTCTTAAACAAGATATGAAGTTGAACTCCAGACTGACTTCTTAGAAAAATAGATAATCTTCTACAGAAGTATTCGCAAGCGAACCGTCTGTTAGTATCCTGTTTTAA
>sRNA0090	s0000842	19	146	26	26	20	38	15	21	NC_004350.2	192009	2E+05	-	SMU_t33	63	SMU_191c	2	/+/-/-/	IGR	TTTGGGAGAAATTTTGGGA
>sRNA0091	s0000879	140	91	22	17	16	9	17	10	NC_004350.2	210075	2E+05	-	SMU_217c	162	SMU_218	446	/-/-/+/	IGR	TGGATTAGGCGGTAAGCTAGTCTATAACGAGTGAAAGATACGTAAAAACAGGGAATGTATAGTGTAGTTATAGAAGAGTAATATGAATGTGCAAGGATATGAGCGCATGCTAGACAGATTCATCTGTTTAGTAAAGTAAG
>sRNA0092	s0000899	38	290	52	61	43	66	30	38	NC_004350.2	213475	2E+05	+	SMU_221c	362	SMU_222c	-2	/-/+/-/	AM	CTTAACGGTTTAATTTGAAGAGTCATCCACTTTTCCTA
>sRNA0093	s0000912	96	527	82	108	114	67	67	89	NC_004350.2	215554	2E+05	+	SMU_226c	462	SMU_227c	192	/-/+/-/	IGR	TAGTTCAGTTAATGCTTATTTTTGTTTTATGTGACTTTCAGTTCTTCAGTCCATCTCGCTCCGTTAGGAGCGAGACAAATAAATCAACCGCTATGC
>sRNA0094	s0000913	105	974	172	231	153	135	130	153	NC_004350.2	215649	2E+05	+	SMU_226c	557	SMU_227c	88	/-/+/-/	IGR	CAAGTGCTCAATCTGCGGTTGTATCCAAAAATTTCATAAGTATATACGAAAGGTCATTTATAATCAATATTGGAGCAGTTTAGGTGAGATATTCTGCATATATTA
>sRNA0095	s0000916	43	84	32	13	11	10	12	6	NC_004350.2	216364	2E+05	+	SMU_227c	-40	SMU_228	383	/-/+/+/	AM	ACTAATAAACAGTAGATGATGGTGCTTTGATGATGGATCATTC
>sRNA0096	s0000926	34	73	9	17	13	10	13	11	NC_004350.2	216775	2E+05	-	SMU_227c	371	SMU_228	-19	/-/-/+/	AM	GTATTAATTTTTACCGTCATTTTGCTACCTCAAA
>sRNA0097	s0000931	31	87	15	24	19	12	8	9	NC_004350.2	218806	2E+05	-	SMU_229	-15	SMU_231	151	/+/-/+/	AM	AAACGACTTGATTTCTTATTCTACACTCATC
>sRNA0098	s0000954	94	1592	263	396	314	195	244	180	NC_004350.2	224768	2E+05	+	SMU_235	81	SMU_236c	-51	/+/+/-/	AM	TCTAGTCTGGTTTCAGGACTAAATAGAAGACTCATAGATTTTTTAATGTGTATCAAGTATCAGCAAAGTTAAAATTTGTTTTTCAATTAGAAGT
>sRNA0099	s0001013	108	115	38	5	23	23	13	13	NC_004350.2	237790	2E+05	-	SMU_248	-86	SMU_249	-16	/+/-/+/	AM	TGAGTATCTAATGTCATGATTTTATCTCTTATCCAATTTTTCATCCAAAACAGCAATCATTTCGTCACGAACAGCTTTAACAGGAATTTCAGTAATGACTGTTCCAAG
>sRNA0100	s0001015	101	102	16	13	24	22	10	17	NC_004350.2	239075	2E+05	-	SMU_249	-35	SMU_250	-78	/+/-/+/	AM	AAAAACCATGATGATGAGGATTTTTCGAATGATCCCCGACAACTGCCATATAAAGACTATCTAATCTAGAGAGTGCCATTAAAGAACTCCTTTGTTTTAAG
>sRNA0101	s0001018	142	1395	298	173	202	254	194	274	NC_004350.2	239503	2E+05	-	SMU_250	-28	SMU_251	-92	/+/-/+/	AM	AGTTGATGCAATCGGTTTAACATCATCATGAAAGCCAAATTGATAGTCTCCTATGTCAATTGGCTTTGGTTCTACTCTTTCGTTTTCTGGCATAATACCCTTTCCTTAATTCTTTAATTTTCTTGTCGCTCAATAGCTTTCT
>sRNA0102	s0001020	128	481	71	83	75	89	82	81	NC_004350.2	240878	2E+05	-	SMU_251	-89	SMU_252	254	/+/-/+/	AM	TAGCTCATTGTTTTTAAAACTTAATCATAACGTTTTAATTAACCAACTGAACCCTCCATCTCATAGGAAATCAAACGATTGAGTTCAATCGCATACTCCATCGGAAGTTCCTTGGTAAAAGGCTCAAC
>sRNA0103	s0001059	94	286	47	64	53	37	49	36	NC_004350.2	250156	3E+05	-	SMU_259	642	SMU_260	-79	/+/-/+/	AM	TCAGCCTTTGATAAATCAACAGTTTTGCCTAAGGCATAGATACTGCGACGTTGTTTTTGTAAATCTAAAAAATTTGACATAAATAATTACCTCC
>sRNA0104	s0001060	109	80	22	4	14	21	10	9	NC_004350.2	250692	3E+05	-	SMU_260	-80	SMU_261c	189	/+/-/-/	AM	TTACTTGTTAAAATAAAATTTTCAATGACTAGTCACCAAATACTTTGAAACGTTCTTGGTCTGCCATGAATTCTTTCTCACCAGCAGGTGCTTCAATTGAACCAAAAGG
>sRNA0105	s0001086	79	128	24	31	26	22	19	6	NC_004350.2	256989	3E+05	-	SMU_265	229	SMU_267c	205	/+/-/-/	IGR	ATTTTTTTGATAATACCAAATGTAATATTCAAAGATTTCCCTGATTCCATTAATTCTGAGACTAGTATATTAAAGAGTG
>sRNA0106	s0001087	118	129	22	25	24	36	10	12	NC_004350.2	257011	3E+05	+	SMU_265	251	SMU_267c	144	/+/+/-/	IGR	AGAATTAATGGAATCAGGGAAATCTTTGAATATTACATTTGGTATTATCAAAAAAATCAGCTAAATGTAAGTTTACATTTGACCGTTATTAAAAAATTTCGCCTAAATGTAAGTTTAC
>sRNA0107	s0001088	57	356	55	71	61	46	47	76	NC_004350.2	257061	3E+05	-	SMU_265	301	SMU_267c	155	/+/-/-/	IGR	TTTAGGCGAAATTTTTTAATAACGGTCAAATGTAAACTTACATTTAGCTGATTTTTT
>sRNA0108	s0001090	36	458	71	112	82	85	59	49	NC_004350.2	257115	3E+05	-	SMU_265	355	SMU_267c	122	/+/-/-/	IGR	ATTTTTTAAATGATGGTCAAATGTAAACTTACATTT
>sRNA0109	s0001093	68	131	17	27	28	33	14	12	NC_004350.2	257158	3E+05	+	SMU_265	398	SMU_267c	47	/+/+/-/	IGR	AATGTAAGTTTACATTTGGATGAAAAATAAAAAAATGAGGCTGGGACAGCAATCATGCGTATCTGTTC
>sRNA0110	s0001094	127	91	17	10	14	14	15	21	NC_004350.2	257203	3E+05	+	SMU_265	443	SMU_267c	-57	/+/+/-/	AM	ACAGCAATCATGCGTATCTGTTCCAGCCTCTTACAGTGTAAAAATGAAAGGAAGCCTATGCAATAATAATTATATCTTGGTGCTTATTTCAGGAAAGAGCTTGGCTAGAATTTTGCCTGTGATAACT
>sRNA0111	s0001115	38	121	25	25	12	19	22	18	NC_004350.2	261260	3E+05	+	SMU_268	237	SMU_270	58	/+/+/+/	IGR	TGTATGTGTGATTATTGGTCTTGTTGTTAGCATTTTCA
>sRNA0112	s0001118	138	109	29	12	21	15	21	11	NC_004350.2	262820	3E+05	-	SMU_270	8	SMU_271	-77	/+/-/+/	AM	CAAAGCATTTTCAACCTTCATTTTAATAACCATTGATGAGCCCATGCCATTTCCGCATGCTGTAAGAACTTTAACCATAATTTTCCCTCCTATAAATGATCGTGCCTATTATAACACAATGATAGGCTAAATTCATAT
>sRNA0113	s0001124	114	179	28	33	48	35	20	15	NC_004350.2	263686	3E+05	-	SMU_272	-26	SMU_273	-48	/+/-/+/	AM	AGTTAGAATGGTCCAGTGCAACCTGTAAATTTGGCAATTGTTTTGTCATTTATATCTCCTTCTTTCTACGTACTAACTCATTTCTTCTCATGAATCAAGGTCAAGCCCCTCAAG
>sRNA0114	s0001126	57	61	11	2	20	13	4	11	NC_004350.2	264388	3E+05	-	SMU_273	-28	SMU_274	-22	/+/-/+/	AM	TAAATACCAATTGGACGTGCCATAAGCTCTACCCCCAGATCCGTTTGATTTCGTCTT
>sRNA0115	s0001165	131	476	71	97	83	68	64	93	NC_004350.2	268250	3E+05	+	SMU_281	2	SMU_283	943	/+/+/+/	IGR	TAACAGCTTCTAGTTATCTATTTGAATCATTTCTATATATTGGAAATCAGTTGTATGACTTCAATTGGTACTATACTTATTAAAGTAGGTGTAGAGTTAACTTTAATAATTTCAATAGTATTTTCTTTTGG
>sRNA0116	s0001175	61	79	10	17	18	12	9	13	NC_004350.2	268466	3E+05	-	SMU_281	218	SMU_283	797	/+/-/+/	IGR	AATAAGTGCTAATACACAAAGAGCAAGTAACACTAAAATTGACTGAATTATGTGCATAAGC
>sRNA0117	s0001176	78	184	20	39	46	26	24	29	NC_004350.2	268473	3E+05	+	SMU_281	225	SMU_283	773	/+/+/+/	IGR	CACATAATTCAGTCAATTTTAGTGTTACTTGCTCTTTGTGTATTAGCACTTATTTCTTACCGTAGTAAAAACATTATT
>sRNA0118	s0001233	100	146	46	10	10	36	22	22	NC_004350.2	279927	3E+05	+	SMU_291	57	SMU_292	371	/+/+/+/	IGR	TAATGTTAAAAGCTTTTAAAAACAGCTTCTTAGAAATATTGATTTTGACCTGCATCTCAAAAGTTAGCCTAAAATCTAACTTTTGGGGTGTTTTTCTATG
>sRNA0119	s0001242	43	86	6	11	18	25	6	20	NC_004350.2	281447	3E+05	-	SMU_292	49	SMU_293	40	/+/-/+/	IGR	AATCCGTATAAAAATAGGAAACTGACGCCGTGTCGCCAAGACA
>sRNA0120	s0001265	111	602	84	131	141	54	115	77	NC_004350.2	287424	3E+05	+	SMU_298	165	SMU_299c	-82	/+/+/-/	AM	TAACAATATGAACGATTATCTTAATGACTTAAGCGTAAGCGGCAAAACTTGCTGCACCTGAAGCCCAGCTATTTACTACAACGTCTGTTAAAGCTTGTGCTGGAGTTTTTG
>sRNA0121	s0001268	139	72	15	17	12	12	8	8	NC_004350.2	287690	3E+05	+	SMU_299c	20	SMU_300	254	/-/+/+/	IGR	TATTGTTAGTATATCTACTAACATCGTCATTATAACTGTTTTTAGTAGAGATGTGAACCTGAAACCGTTTCAAAAATCGGAACTTAACTGTTGTGTTAATCGAGTGCAAAAGACTTAATTGGATATTAATTTTCAATTG
>sRNA0122	s0001299	48	81	11	14	17	16	8	15	NC_004350.2	292533	3E+05	-	SMU_304	-17	SMU_305	-33	/+/-/+/	AM	ATAGCCATTCCTGTGGCTTGGTGATTGTATTCATTTTGTTCTCCTGTA
>sRNA0123	s0001302	80	1047	162	231	178	157	160	159	NC_004350.2	293097	3E+05	-	SMU_305	-13	SMU_307	491	/+/-/+/	AM	TGACCCGCTTCACGCAAAGCTGTTGCTCCGATATCTATTATAGCATAAAGTAAAAAAACTGCCAACTTAAGACTTTTTCT
>sRNA0124	s0001303	43	117	16	17	6	27	25	26	NC_004350.2	293161	3E+05	-	SMU_305	51	SMU_307	464	/+/-/+/	IGR	TCGCTTAGGGCTGCTGGATTCCTCCCCTGACCCGCTTCACGCA
>sRNA0125	s0001306	102	201	41	27	33	47	27	26	NC_004350.2	293337	3E+05	-	SMU_305	227	SMU_307	229	/+/-/+/	IGR	CAAGATTTTGTCCTCTAATTGATTTACTCTGATCACTTAATAAAAATCGGATGGAATAACTAACATCTTCTAAATCAATAAATCTTCGATAAGGAATTTTAG
>sRNA0126	s0001348	120	8997	1470	960	2904	1360	712	1591	NC_004350.2	303739	3E+05	-	SMU_318	32	SMU_320	183	/+/-/+/	IGR	CATCCTAAAACAACCACTTCCCCAAGTGGTTGGGGAGATAAACTTTCCTATTTTATTTCCCCTTTTTCATAAATCTCCTGGGCATTCAATGAAATTGGATGCCCTTCCCTTTTTCTTAAA
>sRNA0127	s0001349	48	939	149	171	156	153	151	159	NC_004350.2	303747	3E+05	+	SMU_318	40	SMU_320	247	/+/+/+/	IGR	AAAGGGAAGGGCATCCAATTTCATTGAATGCCCAGGAGATTTATGAAA
>sRNA0128	s0001350	40	107	17	19	13	20	30	8	NC_004350.2	303779	3E+05	+	SMU_318	72	SMU_320	223	/+/+/+/	IGR	CAGGAGATTTATGAAAAAGGGGAAATAAAATAGGAAAGTT

>sRNA0129	s0001351	41	313	69	50	52	58	52	32	NC_004350.2	303806	3E+05	+	SMU_318	99	SMU_320	195	/+/+/+/	IGR	AAATAGGAAAGTTTATCTCCCCAACCACTTGGGGAAGTGGT
>sRNA0130	s0001352	135	5243	966	995	765	955	911	651	NC_004350.2	303832	3E+05	+	SMU_318	125	SMU_320	75	/+/+/+/	IGR	ACTTGGGGAAGTGGTTGTTTTAGGATGATTAAAGTATATATGAGAAACCTTAAAGAAACCTTAAAGTAAAATATTTATTTTATTAGAATAAGAACCCTTTTGTTACAAGCTTTTTTAATGTTTTATGACCTTTTG
>sRNA0131	s0001360	146	190	40	39	38	33	27	13	NC_004350.2	304505	3E+05	-	SMU_320	-72	SMU_321	-86	/+/-/+/	AM	TAAATAAACAACTTGCATCGTCAAAAAAACGAGAGTCGTTAGCAGGACTAAAAAAGAAGTTGCAGGATATTTTTTAAAGGTCTCCACTGACCAACACCTCCTTTACTGGAATGTCAAAAAAACTAGGCTCAAAAGTGAACTTTTGA
>sRNA0132	s0001425	112	663	126	149	103	102	80	103	NC_004350.2	321659	3E+05	-	SMU_339	-71	SMU_340	183	/+/-/+/	AM	TTATTGAATAACATCAAGTAAATCTGTAAAATTGAACTATTCAATATCTAATCATCATAACACAAAGCAACAAGATATAAAGGTTACTTGAATAGAGGAAAAGACGTTCAAA
>sRNA0133	s0001431	109	544	131	50	104	63	100	96	NC_004350.2	323328	3E+05	-	SMU_341	164	SMU_342	-104	/+/-/+/	AM	TTCGACGACGAGGACTTCTTGTATTTTGATTTTTTCTGTCATAAATTCTATTTTTTTGACAGCTTGCCTAAGGTAGCATGGACGCAAGCAAACCTTTGGTTTCATTGCT
>sRNA0134	s0001447	130	691	128	106	142	115	108	92	NC_004350.2	326233	3E+05	-	SMU_346	76	SMU_348	-105	/+/-/+/	AM	AATGCTGATGATCGCCAACAACAACATAACCAGTTTCTAATTCTTTAACAAAATAAGGATTGGTTCTTGATTTAATCCTATCAATCCGCTCACAAATGAGGCACATGTTGTTCTTCTCCTGAGTATATAA
>sRNA0135	s0001474	100	553	78	113	117	62	79	104	NC_004350.2	333976	3E+05	-	SMU_356	141	SMU_357	20	/+/-/+/	IGR	TTTATTTTTGGGTGATTAGGCACTTGGTGACAGTCCTAATCTGTGTGTACTTTGCAACATTTGTCAGCACGTCTCTGTACACTTTTGAGAGACCAAAAGT
>sRNA0136	s0001478	124	189	32	36	45	22	28	26	NC_004350.2	334493	3E+05	-	SMU_357	-15	SMU_358	-90	/+/-/+/	AM	TAATACGGTTGATAAGACGTGTTACAAGTTTTGAATTGTATAATGGATCTGGCAATACTTCGCGCTTAGGCGCACGGTTTTTACGACTCATTTCTTAATCTCCCTTCCTTATGCTTTTGGTTTC
>sRNA0137	s0001484	98	525	102	89	99	84	78	73	NC_004350.2	337185	3E+05	-	SMU_359	-61	SMU_360	259	/+/-/+/	AM	TTTATAAAGGATTAATTAGCATGTTCACTAAAGAAATTAAGCTTCTCCAGCGTTTTTCTTAATGATCTCTTCTTGAACAGATTTTGGAACATCTTCAT
>sRNA0138	s0001500	90	236	42	40	49	46	21	38	NC_004350.2	340126	3E+05	-	SMU_361	-30	SMU_362	328	/+/-/+/	AM	TATCATTACGATTTCGCACTTTTTTACAAAATCAGGATTGAGACTTACAGGCGTACATCTTATTTTTCCGTGAGTGCAGCGAGGCCTGGG
>sRNA0139	s0001518	25	100	23	13	19	11	14	20	NC_004350.2	342895	3E+05	-	SMU_364	-5	SMU_365	141	/+/-/+/	AM	TATCGGCTGTTTTCTAACCTTAATA
>sRNA0140	s0001525	117	327	48	36	76	67	52	48	NC_004350.2	348953	3E+05	-	SMU_366	-62	SMU_367	94	/+/-/+/	AM	AATTGATAACAAAGAAAAGCCTGAGACGAAAGTCCTGTCTCTTTTGTTCTACTCTCACTCTGTAACCATCATTCTGAGGTTTTGATCAATTTTCTCTGCTGTTTTGCGTCCTTCTCG
>sRNA0141	s0001530	69	2476	312	604	572	332	336	320	NC_004350.2	349790	3E+05	-	SMU_367	-8	SMU_368c	126	/+/-/-/	AM	CAAGCCTCAAAAAGCTTGTCGTCAATCTGATAAAAGAAAATCTTCTCTAATTTTTTTATTTTAGTTAGG
>sRNA0142	s0001531	86	914	176	166	208	134	110	120	NC_004350.2	349875	3E+05	+	SMU_367	77	SMU_368c	24	/+/+/-/	IGR	CAAAATAAAAAGCCAATGTCTGTAGACACTGGCTAAGGATGGGGCAAAGAATAGCGTTTTTGCTTAGGCTTAGACAAGCTCTTTAA
>sRNA0143	s0001535	130	233	37	41	42	46	37	30	NC_004350.2	351601	4E+05	+	SMU_368c	-65	SMU_369c	-62	/-/+/-/	AM	AATCTCACCTAGACCTCCAATAGCATAAACGCCAACTTCTTCAGGTTTTAAATTAATATTTGCCATTTTAGAACTCCGTTAATTGAAAGGCACCTGTTTCTTTTTCATACTCCAAGTGTTTATCTGAAAG
>sRNA0144	s0001536	131	89	12	32	11	10	12	12	NC_004350.2	351800	4E+05	+	SMU_369c	-98	SMU_370	430	/-/+/+/	AM	CAGTTCGGTTTGAGCATCAATATCGAGGTATAAAGTTTTGGTTTGTTCGCGGCGTGGGCTGCTGTCTTTAGTTTCTTGATAGAAAACTTTGTAAATCATAGTGTTCCTTTCTGTCATATCATGATTAATCC
>sRNA0145	s0001556	60	66	16	13	10	8	10	9	NC_004350.2	358794	4E+05	-	SMU_376	69	SMU_378	67	/+/-/+/	IGR	AAAGCATAAGACCCCCAACCATTTAATTCGTCAACTTCAATTTCTTGATAAAGATTCACA
>sRNA0146	s0001568	95	70	6	19	16	6	15	8	NC_004350.2	359440	4E+05	+	SMU_379	166	SMU_381c	247	/+/+/-/	IGR	AGAGCTAGTTGCTATAATTAATAATTTACTAGAGATTGTCTAACTGAAGAGAAGTAGTGTCTAATAGATGTTCATTATTAGCGCACGGCCATTAC
>sRNA0147	s0001621	139	1085	120	242	213	211	172	127	NC_004350.2	368938	4E+05	-	SMU_392c	110	SMU_393	-113	/-/-/+/	AM	TTTATCATTCTTTTCAACATACTCTTCAGGAAAAGATTTGACACGATTATTAACCGCTTGTCGGCTCACTCCTAAAATCTCAGCCAATTCGCTGACTGTCTTTTCAATTGCCATAATATCCTCATTTTTACAGAATTAA
>sRNA0148	s0001624	76	85	4	11	29	15	15	11	NC_004350.2	369440	4E+05	-	SMU_393	-38	SMU_394c	83	/+/-/-/	AM	TATAACTGCTTAAAAAGCCTCTACATACAGAAGTCCCTTATTTTCCACCAAATAAACGTGCAAAAAAGCCTTTTTT
>sRNA0149	s0001639	31	78	12	10	14	13	15	14	NC_004350.2	372523	4E+05	-	SMU_395	91	SMU_396	-6	/+/-/+/	AM	CATCCATTTGTCTGTTATTCCTCCGCATTTT
>sRNA0150	s0001658	78	583	108	82	85	98	65	145	NC_004350.2	375900	4E+05	-	SMU_401c	33	SMU_402	52	/-/-/-/	IGR	TTTGCTTTCGGGTACGATCAAAGCAGTGAGAGCTTTTTATATTCTAAAAACTCACAAATTCAGAAAAAACAGCTCTTG
>sRNA0151	s0001661	142	1588	282	347	316	226	205	212	NC_004350.2	375979	4E+05	+	SMU_401c	112	SMU_402	-91	/-/+/-/	AM	AAAGACCTCACTTTTATGAGTGAGGTCTTTTAAACTAAACTGTTTAACTCTTATTTGTTGTTAACCAAGTCTGTAGCTGCATCATCCATTGAAAGAACTTCATGGAAAACACGTTGTGTCAATTCAGTCTTTTGTTCTTTAG
>sRNA0152	s0001676	83	201	35	34	37	38	21	36	NC_004350.2	381362	4E+05	+	SMU_406c	-5	SMU_407	232	/-/+/+/	AM	AATCATAGCAAAATTATAGCAGAAAAGAAGAAAATTAAGCAAGTGAAGGGATGATTAGTGTGCTTTTTGAGCGAAATATTTCA
>sRNA0153	s0001681	47	83	13	7	37	6	15	5	NC_004350.2	381454	4E+05	+	SMU_406c	87	SMU_407	176	/-/+/+/	IGR	TGAGGATGCTCTTACTTAAACCAAACTGTTTTTACTACTATATAAGG
>sRNA0154	s0001700	114	1732	343	375	293	307	210	204	NC_004350.2	384465	4E+05	-	SMU_409	118	SMU_410	100	/+/-/+/	IGR	TATGTGCTAAAATCATCACAAGCTCTATCACTTGGCCGACTAGCTTACAATCACACAAATTTCATTGACTGATTGAGCTTTTTTCAATAAAAGCAGCCATTTCTTGAGCAGTTA
>sRNA0155	s0001702	105	136	29	32	22	24	11	18	NC_004350.2	384597	4E+05	-	SMU_409	250	SMU_410	-23	/+/-/+/	AM	TAGAATTTTTTTACCAATCTTCATTTGTTTTTCTAATCTATCAGTTTCCTCCTAAGGTAAACATCAAGAAATACTCCATCTTTTGTTTTTGAGCCGTTCATTCCA
>sRNA0156	s0001709	147	321	56	56	75	47	43	44	NC_004350.2	386684	4E+05	+	SMU_412c	-93	SMU_413	12	/-/+/+/	AM	AAATATCAAGAAAGGCTAGGACATCCTCGTCCTCATAAACTTTTGATGAGGGAATGTCACCAGCTACAATTTTACAAAAAAGACAATCGTTCATAAAAAATCACCTACTTAACTTGTTTTTGTTATAATATAGTATATCAAAGTTAG
>sRNA0157	s0001710	71	186	31	44	45	20	24	22	NC_004350.2	386803	4E+05	-	SMU_412c	26	SMU_413	-31	/-/-/+/	AM	TAACCACCGGTCAAATTTTCTATTTTTAACATGACGTTTCTTTCTAACTTTGATATACTATATTATAACAA
>sRNA0158	s0001737	68	336	75	53	59	59	35	55	NC_004350.2	395487	4E+05	-	SMU_421	25	SMU_422	142	/+/-/+/	IGR	AAAGAAATCATATGACGTTCATCTAAGGCAACACGACAACGGCTGTACTTGCGTATGCCTAGAAGTGT
>sRNA0159	s0001738	128	193	38	31	22	50	30	22	NC_004350.2	395551	4E+05	-	SMU_421	89	SMU_422	18	/+/-/+/	IGR	TAATTTTACAATTTTCTTTTTTCACCACGACAAAAAATATCTTGTAAAAGCTCACTTCATTCGCTCATCTTCTGAAAAAAGCTAGGCCAGATGACCTAGCTTTATTGTTTATCTTTGAATTTCAAAAG
>sRNA0160	s0001742	25	83	14	17	14	23	4	11	NC_004350.2	396052	4E+05	-	SMU_422	6	SMU_423	455	/+/-/+/	IGR	CAAGCAGACAGAAGAAACTTTTTTG
>sRNA0161	s0001755	101	349	72	33	78	47	72	47	NC_004350.2	396437	4E+05	-	SMU_422	391	SMU_423	-6	/+/-/+/	AM	TATTCATATGATAGATACCTCTTTTCCATTTTTAATTTTTTGTCTGGTTAGCCAACCGACATCTCTAATATAGCAAATTATTTTTCAAAATGGAGTAAAAT
>sRNA0162	s0001757	46	128	24	33	20	22	12	17	NC_004350.2	396525	4E+05	-	SMU_422	479	SMU_423	-39	/+/-/+/	AM	TATCCATTACGTTAAATTGTTCAAATGCTTGTGTATTCATATGATA
>sRNA0163	s0001761	65	136	28	8	38	13	20	29	NC_004350.2	396905	4E+05	-	SMU_423	144	SMU_424	183	/+/-/+/	IGR	AATAATAATTTTTCAAAATGATACAGTCCTCATTAGCTCTTGTGATTCTTGTTAATTTTAAACCA
>sRNA0164	s0001762	47	96	22	12	31	10	12	9	NC_004350.2	396959	4E+05	-	SMU_423	198	SMU_424	147	/+/-/+/	IGR	TTTCTGCCTCTTCACTCAAACAATGAACAGTTCTTCAATAATAATTT
>sRNA0165	s0001768	110	442	94	46	102	66	69	65	NC_004350.2	397128	4E+05	-	SMU_423	367	SMU_424	-85	/+/-/+/	AM	ATAGCAATAATTTCACTGCTGCTAGTCATCTGCTTAGCCCAGACAACACGCATTACTTCCCATTCCGCATTTGAAATAGATGTCATTTGAGCTCCTTTCATCTACATTTG
>sRNA0166	s0001777	36	168	25	12	40	22	30	39	NC_004350.2	400090	4E+05	-	SMU_427	54	SMU_428	152	/+/-/+/	IGR	TTTCCCAGTCTTTTCCCTCTTTGTGACCATCTGATA
>sRNA0167	s0001792	96	912	146	172	198	139	117	140	NC_004350.2	401764	4E+05	+	SMU_429c	62	SMU_431	195	/-/+/+/	IGR	AAATCAAAATGATAGGTTGTTAACTTCGTTTTGCTGTACGTTAGTATGGCCTGCAACTCGTCGCCTAGTTTAATTTTAATTTTTTGAGTATTAAAT
>sRNA0168	s0001824	42	103	15	12	12	18	13	33	NC_004350.2	407614	4E+05	+	SMU_437c	15	SMU_438c	84	/-/+/-/	IGR	AAAAGTTAGATTTTTTCTGTCTAACTTTTGGGGTGCAGTTCA
>sRNA0169	s0001828	90	69	9	13	15	2	12	18	NC_004350.2	412023	4E+05	+	SMU_438c	-17	SMU_439	209	/-/+/+/	AM	AATACCAGCTTTATACATATAAAATTACCTAGTTTGACTGTAAATTGTAGAATTTTAAACTGATTTGTAAAGAAAGGAGCTTCTTAATTC
>sRNA0170	s0001829	119	140	29	11	43	15	28	14	NC_004350.2	412094	4E+05	-	SMU_438c	54	SMU_439	109	/-/-/+/	IGR	TATAACAAACGGACTGTTTGATAGGCAACAATCTGTTGTATTGTCACTGTTATTCCGTTGATAAAAATGTGACTAGAAGGGAAAATTGCAGGCTGAATTAGAATTAAGAAGCTCCTTTC
>sRNA0171	s0001842	142	364	65	42	86	68	48	55	NC_004350.2	414290	4E+05	-	SMU_442	-33	SMU_444	91	/+/-/+/	AM	TTTATTTTGTTTCACCTGACTTACACTCTCTCAGGTTCGCTGCAGAAACGCCACAAAACTCTCTTTCTTAACAAAGATTATAGCAAAAAAGGTCAAGCTTGTCCAGACTTACTCCAACTTCATGGCGTTAAAAATATACATA
>sRNA0172	s0001845	40	205	22	51	43	30	35	24	NC_004350.2	414450	4E+05	-	SMU_442	127	SMU_444	33	/+/-/+/	IGR	TATTACTTCATTTATCTGATTGTTAGCTTGGCTATTAGCC
>sRNA0173	s0001865	27	96	13	23	11	21	13	15	NC_004350.2	418116	4E+05	+	SMU_447	23	SMU_448	75	/+/+/+/	IGR	TTTTATAATCTGGAACTTATCTTTTCA
>sRNA0174	s0001869	130	71	16	15	10	14	14	2	NC_004350.2	418542	4E+05	-	SMU_448	-55	SMU_449	415	/+/-/+/	AM	TTAGCCTAAAACTACTGCACAATAAAAGGGCCGAAACATTGTTCCGCCCCTTTTATTAGTGTCAAAATGACTGTCTAATCATTTACATCTTCTGTCTCCTTGGAAATAATTTTTCCCGTATCAGCATTGA
>sRNA0175	s0001875	126	60	6	8	13	14	12	7	NC_004350.2	418806	4E+05	-	SMU_448	209	SMU_449	155	/+/-/+/	IGR	TAAGCAAACGATTAATTTTTTAAAAAGGAGTAGGACAAAATTGATAAACCGCAATGAATGTGATGTCAATTTTATAATCCAGCTTTTGCAAAGTGGACTTGGTATTGATATGAACTGAGGCTTATG
>sRNA0176	s0001884	101	109	15	21	23	28	14	8	NC_004350.2	421126	4E+05	-	SMU_450	-23	SMU_451	35	/+/-/+/	AM	AAGTGACGTTTTATTTACATTCTCTATTCAAAATTTTGTCTGGCATTCCTCTTGTGAACTTCACTTCTAATCATCATTTATTCTCTAATCTGTCCTTTACC
>sRNA0177	s0001903	92	97	22	23	30	10	7	5	NC_004350.2	426078	4E+05	-	SMU_456	69	SMU_457	-66	/+/-/+/	AM	AAAGCCCAAGAATAGAAAAAGCAGGGAGGATAAATCTTCCAATAAGAACTCCAATATGATAAGGCAAATCTCCTGACCTAACTAGTATTACT
>sRNA0178	s0001926	78	201	48	28	62	29	11	23	NC_004350.2	431659	4E+05	-	SMU_463	45	SMU_464	342	/+/-/+/	IGR	TTATTTTTCTATTTATAAGCAAAAAAGGTTAGGATAAAGTGGTATGTTAGAGTAGTTATCTAACTTTTGAGACACACT
>sRNA0179	s0001930	117	96	11	13	18	27	12	15	NC_004350.2	431837	4E+05	-	SMU_463	223	SMU_464	125	/+/-/+/	IGR	CAATTATTAAGATACAAAGAGCATTGCGAGCAAAGGCAAAATAGGAGGTTCGTGAAGAGTTTCAGGACTCTAGGAGAAGCTGTCTTTTTGACACAGATCGCAGCTCGTGTTCAATTA
>sRNA0180	s0001933	33	70	18	2	12	13	17	8	NC_004350.2	431965	4E+05	-	SMU_463	351	SMU_464	81	/+/-/+/	IGR	ACATAAGGCAGGCTATCTTTTTCTCGACGTTTT
>sRNA0181	s0001934	78	656	116	99	120	146	89	86	NC_004350.2	431984	4E+05	-	SMU_463	370	SMU_464	17	/+/-/+/	IGR	TATTAAGATACAAAGGGCGTTAAGAAAACGAGAAGAAAATAGGAGGCTGACACCGTGCGCTTGCACATAAGGCAGGCT
>sRNA0182	s0001948	108	208	42	26	41	48	23	28	NC_004350.2	438761	4E+05	-	SMU_469	22	SMU_470	-49	/-/-/+/	AM	AAAATTCCCAATTCAAAATTTTTATAACCCGTTACAAGAATTGTCGTCATAAACTGTTGTCCTTTTCAATTTTATGATATAATAGTAGTGTTTTATATTATGAATACT
>sRNA0183	s0001952	139	460	74	102	80	91	62	51	NC_004350.2	439380	4E+05	-	SMU_470	40	SMU_471	-72	/+/-/+/	AM	TATCATAGCCACGCATGCTAGATTTAAATTCTTGTTCAAAAATATCTTTAGGGGTGTACATAATACTTGCCATACTTTTATCTCTCCAAACATATCATTTCGACCAGCTTACAGCTGTTCCATCATTTTTAATCATACA
>sRNA0184	s0001955	45	117	25	22	13	24	23	10	NC_004350.2	440198	4E+05	-	SMU_471	414	SMU_472	-8	/+/-/+/	AM	TTTTTTCATGATTCCTCACTAAAGACAATAAAAGCTAGCCAAAGC
>sRNA0185	s0001969	107	539	88	70	112	88	88	93	NC_004350.2	445464	4E+05	-	SMU_475	25	SMU_478	316	/+/-/+/	IGR	CAAAGTTAATTGGGTATTGACACAAACTTTCCTTCATGGAATTAGACAGGGATTTCCGACCAACCGCTGTGTTAAACGGAAACCACTGCACAATAAAAGGGCGGAAC
>sRNA0186	s0001988	148	861	119	212	187	143	97	103	NC_004350.2	451532	5E+05	-	SMU_482	-51	SMU_483	-57	/+/-/+/	AM	TAAAATCTTGATTATTAGAACGCTTCTGTCCAATATCTGTAAAAAGGGAAATTTTCATATGCTCGTTGTCTGACAAGGTCAGCCTCCTCCTAAATCTTACGAAATACGCTTAACTTGGCTGATAAAAAAACCGTCTGTTAGATATTGT
>sRNA0187	s0002018	118	121	30	2	16	58	7	8	NC_004350.2	460778	5E+05	-	SMU_491	234	SMU_493	-102	/+/-/+/	AM	TATCACGATAACTGTACATGCGTTCCGTCAAATAACCAAAGTGTTTTTGAGAATTCTTTTGAACATCATTTGTGTACTTTTGAGTTAAAAGCGGAATTACCATTCTCCTTCTCCTTTT
>sRNA0188	s0002047	103	70	14	16	6	9	8	17	NC_004350.2	469726	5E+05	-	SMU_500	18	SMU_501	4	/+/-/+/	IGR	AATCTCCTTCTCTGAATATTAAAAATATTACAAAATAATTATAGCATGGGAAACTTACTCTGACAAGGCTATAAAAAAAGCCAACCCACTTAATGAATTGACT
>sRNA0189	s0002058	111	100	33	5	7	25	20	10	NC_004350.2	473254	5E+05	+	SMU_503c	-107	SMU_504	266	/-/+/+/	AM	TTGCTGACTGTTTTCTCTTACTGAATGGTGATTAAACAAGAATAAAAATAAGGCGAGCATCAAAGAGATAAGAGCTGTTAGAGCTATTTTGATTTTTTTAGGTGTCATAGT
>sRNA0190	s0002082	34	390	80	80	50	76	50	54	NC_004350.2	478302	5E+05	-	SMU_508	-12	SMU_509	48	/+/-/+/	AM	AAAAACCACTTGATCTCTGTTTTAAATTAAAACA
>sRNA0191	s0002084	55	61	8	13	14	10	6	10	NC_004350.2	478353	5E+05	-	SMU_508	39	SMU_509	-24	/+/-/+/	AM	TAAAAAAGTCAGATTCAAAAGACATGGCAATTCCTTTCTTATAGCCCCTATTATA
>sRNA0192	s0002089	128	285	30	86	43	53	40	33	NC_004350.2	480245	5E+05	+	SMU_510c	88	SMU_512c	-87	/-/+/-/	AM	ATTAAAGAACAAGTCTATAACGATCTTAAAAGTAGTCTGTTTATCGGGCTAGGAGATAGTGCTGGCTGGCATTTTTCTTAGAGGTTTCACTCGCCTTCACCTGCTCAGCTAGCCCATTGAGTTCAATG
>sRNA0193	s0002104	81	29604	4497	4637	6895	3370	6095	4110	NC_004350.2	481422	5E+05	-	SMU_513	53	SMU_514	88	/+/+/+/	IGR	TGGACTATGCTGTTAGTGATCAAACGAACAGGAGACATCCAAATGAACGACTTAAATGATACCGCTAAAAGTCATCATTTG

>sRNA0194	s0002136	73	93	17	32	18	11	10	5	NC_004350.2	489701	5E+05	-	SMU_522	-13	SMU_523	-59	/+/-/+/	AM	CCAAGATAACTTGCGGCCGATTGGTTTTAACTCAGCTGTTTCATCAAAAAAAGCAGTCATTACTGAGCTGTTC
>sRNA0195	s0002150	97	77	31	14	10	7	9	6	NC_004350.2	494749	5E+05	+	SMU_526c	-38	SMU_527	239	/-/+/+/	AM	AATGTAGGAAAAATCTCCAATTTTTAAAATAAACTCCATAACATTTCTCCTTGACTCTATAGTTACTATAGATATTATAATATATTTGTGACATAAA
>sRNA0196	s0002152	79	392	53	83	83	51	64	58	NC_004350.2	494833	5E+05	+	SMU_526c	46	SMU_527	173	/-/+/+/	IGR	TTTGTGACATAAATATGGCTGTTTAGTTTATCTTTTATAGTGTTGTTAATCCCCTTGTTTAACTCAAGTTATTCTGCAG
>sRNA0197	s0002157	127	142	29	26	26	18	20	23	NC_004350.2	495030	5E+05	-	SMU_526c	243	SMU_527	-72	/-/-/+/	AM	CAAGATAATTAAGAATGACCTTTGACATTTTACCACAGCCATATTGGACAGCACGAATTTTTGTATTTCTCATGAGAGGTTCCTCCTTTTATTGTTATAGTCTTTTAGTACTAGGCAGATACAAATT
>sRNA0198	s0002161	73	92	16	11	22	16	17	10	NC_004350.2	496509	5E+05	-	SMU_529	-36	SMU_530c	144	/+/-/-/	AM	AAATGTAAATGTCTTAGGTTATCATCAGAAAGGAGACTAACCAATGAAAATAATTAATGCAAGCTTTTTTATT
>sRNA0199	s0002171	97	119	31	10	22	13	21	22	NC_004350.2	497390	5E+05	+	SMU_530c	-72	SMU_531	648	/-/+/+/	AM	CAATGGCGTAACCCCAGTTTTCTTGCAGATGTGCTTTAGCTTCTGCTTTTAGCTCAGCTCTTGTTTTTGACATGAAAAATCTCCTTAATAAATAATG
>sRNA0200	s0002179	113	656	98	122	157	104	65	110	NC_004350.2	497871	5E+05	-	SMU_530c	409	SMU_531	151	/-/-/+/	IGR	TGTGCTCACCGGAAGAATATTGTTTATCTTCGCTTTCTAAAAATCAGCCCACTTCCCAAAATTCTGCTGCCTGTTCACAATACCCACAGACTCCCTGAAAACAAAAGGTTTGG
>sRNA0201	s0002180	23	158	30	37	27	33	24	7	NC_004350.2	497984	5E+05	-	SMU_530c	522	SMU_531	128	/-/-/+/	IGR	TAACAAGGAGTTGCACGATAAGG
>sRNA0202	s0002184	34	270	52	46	53	44	31	44	NC_004350.2	498064	5E+05	-	SMU_530c	602	SMU_531	37	/-/-/+/	IGR	AAAATCTGTGTGAGGGCGTTAAATCTAGCTAACA
>sRNA0203	s0002207	63	322	55	76	49	51	42	49	NC_004350.2	504696	5E+05	-	SMU_538	-29	SMU_539c	204	/+/-/-/	AM	AGAAACAACATTCCCGACTGGCTGATTGAATAATTATTTTTGATAATGGCTTCCTGCATAAAT
>sRNA0204	s0002216	103	128	21	25	19	18	8	37	NC_004350.2	506286	5E+05	-	SMU_540	40	SMU_541	65	/+/-/+/	IGR	TAATAAAAAAAGCCTGACTTCAACAAATGACATATCTCAATTGCTGGACAACTAATCCAACGTCTGGGATATATCAAAAGTGTCAGACTTTCACCTAACATAG
>sRNA0205	s0002276	101	1956	325	416	333	333	262	287	NC_004350.2	520313	5E+05	-	SMU_557	189	SMU_558	221	/+/-/+/	IGR	ACTTTCATCAAAGGACGAAAGCTCGTGGTACCACCTTAATTCAGAAAGTTCTAAAAAAATTTTTCTTAGCCCAAGCAGTGACTCAAGATGGCAAACCTACCA
>sRNA0206	s0002282	67	132	26	17	24	19	21	25	NC_004350.2	520558	5E+05	-	SMU_557	434	SMU_558	10	/+/-/+/	IGR	TTATAATATAATTACGTTTTGCTTTGGAATTAGGAATGAGGAATGAGGGGGTCTCATTCACTATACT
>sRNA0207	s0002294	96	475	73	106	84	78	64	70	NC_004350.2	524133	5E+05	+	SMU_558	707	SMU_560c	-29	/+/+/-/	AM	CTTTCTATATTCTCACTATTTAACTATTAATATTAGAATGAAGCTGTGCTAAGTTTCAGATGATTTCTAATCAGCTATTGGAATGGAGATCTCAAT
>sRNA0208	s0002311	123	186	28	21	21	39	20	57	NC_004350.2	530370	5E+05	+	SMU_566c	15	SMU_567	100	/-/+/+/	IGR	AAAAGTTAGATTTTTTCTGTCTAACTTTTGGGGTGCAGTTCAGTTCTGGACTCCTTTTTAAAGTTCAGTACAAGAGCTTTATTTTTGGTGTTGTTGATTAAATAATAGTTTTTAGGGGACTTG
>sRNA0209	s0002351	63	298	61	36	46	46	32	77	NC_004350.2	540970	5E+05	+	SMU_577	14	SMU_580	367	/-/+/+/	IGR	TATTATTGAAATCTAATACGGACAGTTACTTATTAGCAAAAAAAAGCTCTTTTTCGAAATATTT
>sRNA0210	s0002352	46	64	5	7	11	12	6	23	NC_004350.2	541020	5E+05	+	SMU_577	64	SMU_580	334	/-/+/+/	IGR	TTTCGAAATATTTTTTTGATGACGTCTGTTTCTAGTTTTCTATTGT
>sRNA0211	s0002359	101	63	14	14	5	5	5	20	NC_004350.2	542889	5E+05	-	SMU_581	-49	SMU_582	-54	/+/-/+/	AM	TTTGCGCATAATAATGCTTAATAGCCTGATTAATGGATTTAATCTTATCTTTCACAACTCGTCCATTTCTGCTTCACTGCCATCAGCCTGCATGACTTTGA
>sRNA0212	s0002385	144	856	141	192	138	187	83	115	NC_004350.2	549864	6E+05	+	SMU_590c	-35	SMU_591c	20	/-/+/-/	AM	TATCGTTAGATTTTCTGTCCAACTTTTAGGGTGCAAGTCAGTTTGCTATGGTCTTTTTAATGTTCTATTAGCATGTTTTCTTTTACAGTGAGTGCTACCATAATACTTATTTGTTACTTCCTGTTCTAGCTGTGTCATATAGCT
>sRNA0213	s0002391	54	129	30	21	16	19	14	29	NC_004350.2	551876	6E+05	+	SMU_592c	83	SMU_593	127	/-/+/+/	IGR	TGTATTAATTATCCTAGAGATTGTTTGAATGATTTTTAGAGATAAAATTGATAG
>sRNA0214	s0002402	83	158	27	15	45	20	38	13	NC_004350.2	553697	6E+05	+	SMU_595	-51	SMU_596	187	/-/+/+/	AM	TGAGACAATTATCAAAGTCAAAAGAGCCAATTGTTGTATGAGTTGAAACCATGGAACCTCCAAAATAATGAATTTTTTCTATT
>sRNA0215	s0002417	147	232	33	61	49	34	43	12	NC_004350.2	556934	6E+05	-	SMU_597	-49	SMU_598	-87	/+/-/+/	AM	AAGCTAAGCGAGTCGCTGTTTTGATACCAATACCCGGTAATTTTGTGAAGCTCTCAATAAGTTTGGCAATAGGCGTTGGGTAGAGCATTCTTTTTCCTCACTGATTCATTGGATACATCTTTTGATAAAGATTGATGATATCTCTCG
>sRNA0216	s0002420	78	156	30	45	27	18	19	17	NC_004350.2	557612	6E+05	-	SMU_598	20	SMU_599	115	/+/+/+/	IGR	TCTTCTTTTTGGTATTTCTTTTTCTAAAAAAAAACCAATTGGCCGATATTTCCAATTGGTTTTTTCGTTGAATTGGGAA
>sRNA0217	s0002424	95	640	128	101	137	119	95	60	NC_004350.2	557791	6E+05	-	SMU_598	199	SMU_599	-81	/+/-/+/	AM	TGACACTCTCAGCTGAGAGCACAGAAACATCGCGTTCTGCAGAACGGCCACCATATAATAAAACAAGCGTTTCTTTAGACATAAGATTCCTTTTC
>sRNA0218	s0002427	134	869	184	133	208	146	104	94	NC_004350.2	558803	6E+05	-	SMU_599	-50	SMU_600c	89	/+/-/-/	AM	TTTGCAAAAAGCAAGAAAAAAGACCCTAGCCTCATATCTATTCTTCTTAAGGAACAAGACATGAAACAAGAGCTTTATTATCTTTAAACCAAATGGCTTTCGCGTTTATCAAACATTTCTTTAGCAAGACTAAC
>sRNA0219	s0002429	25	269	38	44	64	49	46	28	NC_004350.2	558950	6E+05	+	SMU_599	97	SMU_600c	51	/+/+/-/	IGR	TATATTTGATTTTTATTGCCTGAAA
>sRNA0220	s0002431	76	295	44	67	46	54	51	33	NC_004350.2	558982	6E+05	+	SMU_599	129	SMU_600c	-32	/+/+/-/	AM	TTATGGGCTACAAAAAATCAGAGAGGGATGTCTCTCTGATTAACTATTTAAAATTTGCAAGTTTTCGTAATTCTGG
>sRNA0221	s0002442	102	117	27	24	19	16	15	16	NC_004350.2	560822	6E+05	-	SMU_602	-66	SMU_603	43	/+/-/+/	AM	TTATAACATAAACAAATTAGAGATTCTCAGCGGATTTACTTTCTTTCTGTTAAATGAGATTGTGAATGATTTTTCCACCAAGATGATAGAATGGAACCAGAA
>sRNA0222	s0002453	57	371	61	69	67	64	39	71	NC_004350.2	563258	6E+05	-	SMU_605	-5	SMU_606	-38	/+/-/+/	AM	AAATCCCATAACAACAAAACAATACTTTTTACCATTCACTGTTAGCTCCTCCTATCT
>sRNA0223	s0002485	56	124	18	18	28	27	19	14	NC_004350.2	575822	6E+05	-	SMU_611	-26	SMU_613	461	/+/-/+/	AM	AAACATATGTTTTAAGAGAATTTGAATGGTTATCTGTCACCTTTATTGCGAATGGT
>sRNA0224	s0002490	84	1115	176	205	217	177	166	174	NC_004350.2	576315	6E+05	-	SMU_611	467	SMU_613	-60	/+/-/+/	AM	AATCTGTATCCGCCACTTCAAAACTATCAAAGGCCATTGAATCTAATGTTACTGTGTTCATAAAATCCTCTTAAAATTCACAAT
>sRNA0225	s0002493	63	250	48	46	37	49	24	46	NC_004350.2	576610	6E+05	-	SMU_613	-6	SMU_614	38	/+/-/+/	AM	TAAAATAGTATAACCATTACAATAAGGCGGAGCGAAGTTAATCAAAAAACGATATATCACCAG
>sRNA0226	s0002498	107	735	166	83	130	144	92	120	NC_004350.2	577203	6E+05	-	SMU_614	353	SMU_616	592	/+/-/+/	IGR	ATAGCACTCGTAATACCAAGTATCACGCCTCCTTCTGATTCAGAAAGATAAGCGATATCCACTGCCACAAAGTCATCAAGAAAGTAGTGACAAAACCTCCGATAGGA
>sRNA0227	s0002499	89	352	73	49	64	56	34	76	NC_004350.2	577299	6E+05	-	SMU_614	449	SMU_616	514	/+/-/+/	IGR	ATTATAGTCCAAAAATAGGGGCTGTGATTGCAAATCCTGCAAATCTGCCAGCAAGCACATCACAATTACACCACTACGATAGCACTCGT
>sRNA0228	s0002504	68	150	35	26	33	19	22	15	NC_004350.2	577617	6E+05	+	SMU_614	767	SMU_616	217	/+/+/+/	IGR	TCGCAATAAGGAAAATAATTCTGAATTTGCAGAGCTAAAAAAGATTTGTCTATAAGCAAGTTAATATT
>sRNA0229	s0002506	40	122	31	21	17	17	25	11	NC_004350.2	577761	6E+05	+	SMU_614	911	SMU_616	101	/+/+/+/	IGR	TAAGAAAAAGTTAAGGATTAAAATGTACTTGTAGTTTTCA
>sRNA0230	s0002509	145	1841	320	282	340	325	295	279	NC_004350.2	577813	6E+05	-	SMU_614	963	SMU_616	-56	/+/-/+/	AM	ACTATCTGCAACTTCAAAATTGTCAAAGGCCATTGAGTCTAATGTTACTGTGTTCATAAAATCCTCCTAAAATTCACAATAAGCTTATTAGCTTACAAGTTTTTTTACTTTCACAAGTTTTCTAATAAAACATGAAATAATTTTA
>sRNA0231	s0002511	124	189	45	20	36	28	30	30	NC_004350.2	578077	6E+05	-	SMU_616	-72	SMU_618	53	/+/-/+/	AM	TTTGTCATTATCAATACCTCAAATGATTGAAAATATTATAATCAAATCATTTTAATCTAATGTTGCTGCACCATAACCTATCATCCCTCCTGCAGCGGCACATGTAAGTAAATAAGCGCCTCCA
>sRNA0232	s0002512	103	311	53	46	62	54	51	45	NC_004350.2	578195	6E+05	-	SMU_616	46	SMU_618	-44	/+/-/+/	AM	TAGATTAGAAAATACTCCTTCCCCATTATTGAAAGGCCAAATCATTAATAAATTAGCTATAACATTTAGAGATGAACAAAAAATAAACGACCATTTTTTTGTC
>sRNA0233	s0002526	137	217	42	33	41	23	52	26	NC_004350.2	580552	6E+05	+	SMU_621c	-34	SMU_622c	-56	/+/+/-/	AM	ACACGTTCTTTCTTTTTTAAAATTGGTTTTGTCATAGTCTTTATTATAGCAAAAAAGTAAAACTGATGATAGTTGGAAAATTAGCTGTTCAACCATTTAGAAAATTGCTTACCCAGCCTGAGAGCAAGCAAGAGATT
>sRNA0234	s0002558	95	175	41	26	29	17	19	43	NC_004350.2	588964	6E+05	-	SMU_629	85	SMU_630	373	/+/-/+/	IGR	TTTTAAACGTCAAACTGAAGGTCTCGAAAATCTTCTCAAATTTTGCCTCTGATGGTCAGTATTTTTTGCACTACGGCGGAAATTGTTCCTTTATA
>sRNA0235	s0002583	111	122	20	26	18	19	21	18	NC_004350.2	591973	6E+05	-	SMU_632	31	SMU_633	-94	/+/-/+/	AM	TTTACTTGACAGCAAGGGGGCAAACGTTCAATGATAAGCTCAAAAGGACTATCTGCTAAATGATTATTTTGACTTTTTAGTTTAAATGTCAGCATGTTATCTCCTTATATG
>sRNA0236	s0002593	120	193	26	38	43	35	27	24	NC_004350.2	595367	6E+05	-	SMU_635	78	SMU_636	-67	/+/-/+/	AM	TCTTCTTCTAACATTCTAAAAGCAACTTTACTACCTTCTGTTTTATTTTTTACTTTAATAGTTTTCATAACGCCCTCCTAATCGGTCTATACAAATAGTATAAGGTATAGACCAATTTTT
>sRNA0237	s0002598	145	3114	496	756	602	456	350	454	NC_004350.2	596280	6E+05	+	SMU_636	160	SMU_637c	-111	/+/+/-/	AM	AATCCTTTTTAAAGTAGAATAGGATGTGTTTGTCTATGTTTTAAACTTTTCAGTGTCTCTTTTTAAAGATACCAACCCAAATCTGGTCCGACAGAAACGATACTTGTAAGATTGATGGTCTTGTGGCTGCCGTAGTAATGTTTTT
>sRNA0238	s0002604	102	150	26	17	27	20	27	33	NC_004350.2	596972	6E+05	+	SMU_637c	156	SMU_638	62	/-/+/+/	IGR	AGAGTCAGCAATGACACCGTCCGCTTATTCAAAGATCTCCCCACTTTCCATTCTGTAAAAGTCCCATGAAATCTCCTTCAATTTAATGATTACTAATTAATG
>sRNA0239	s0002609	109	402	64	68	85	67	52	66	NC_004350.2	597785	6E+05	-	SMU_638	-66	SMU_639	-27	/+/-/+/	AM	CTATCATTTGACGGATAGTGGTAGTCATAGGGGATCCTTTGCTTAATTCAAATAATTTCTAAGGCTGTTCTTCTCTTCGGCCGTCAGCTCCCGATACTCTCCAACTGGC
>sRNA0240	s0002624	94	261	44	22	44	52	32	67	NC_004350.2	600978	6E+05	-	SMU_641	53	SMU_642	99	/+/-/+/	IGR	ACATCACTTTAAAATCTTAAGAGAAAGCGGAATTATTCATGTTAGAAAGAAGGTAGACAACGTTTTATCTCACTTAGACAAGCAGAGTTAGATA
>sRNA0241	s0002636	147	71	21	9	9	10	11	11	NC_004350.2	602588	6E+05	+	SMU_643	138	SMU_644	24	/+/+/+/	IGR	AAAAAGACATCAATTTTTGTTGGGATTTACGAATAATATAAGTGAAATCTACTTTAGTCCTTTTTAAACTATTTGTTTGGAAATTTACTATCAGTCATAATTGAAGTTGTAGTTTTTAAAGCCTGCCTTTGCGCGGGTTTTTTAACT
>sRNA0242	s0002644	57	124	19	14	25	18	14	34	NC_004350.2	605538	6E+05	-	SMU_645	21	SMU_646	136	/+/-/+/	IGR	ATTGACCGCCGATTTTTTCAAGCAGAGAACGACTGGGAAAAGTGTTTTCATCCTAAG
>sRNA0243	s0002655	80	209	29	41	46	29	42	22	NC_004350.2	607150	6E+05	-	SMU_647	46	SMU_648	-63	/+/-/+/	AM	AAGCCGCTAAAGTCACAATAGAAAGCAAAGTCACAAGTCCTGTAGCAATCGTACGTTTTTTCATTCTTGATAAACTCCTT
>sRNA0244	s0002664	112	96	26	12	22	10	8	18	NC_004350.2	608943	6E+05	-	SMU_649	37	SMU_650	67	/+/-/+/	IGR	TTAGGCAGACTATCTATTTTTCGACAATCACAGGACGAATTCCCTTTCCTTTCTTTTCATTAAAAATAGTTGAACGAGACAAACAGAAAAAGCACCTGTTAATAGGATGAAT
>sRNA0245	s0002665	138	431	58	114	95	47	55	62	NC_004350.2	609032	6E+05	-	SMU_649	126	SMU_650	-48	/+/-/+/	AM	AAAAATCTAACCACATTTGGCGGACTTGAGCTGATGTTAATTGTTTCATATTTAGATATAGAGTCCGACTGTAATTGAACAGAAAATTAGGAATCTGACGACAGATACTTGTATCTTAGGCAGACTATCTATTTTTCG
>sRNA0246	s0002667	53	62	11	18	10	12	4	7	NC_004350.2	611693	6E+05	-	SMU_650	-46	SMU_651c	206	/+/-/-/	AM	TAAGTTTTACAAATTCTCTGCTACTGCGGCAATCAAATCTTTGATGGCTGAGG
>sRNA0247	s0002688	105	162	32	28	26	32	21	23	NC_004350.2	620636	6E+05	-	SMU_660	214	SMU_661	-12	/+/-/+/	AM	TAAGTATTGGCATCAAATATATCCTCATTTCTCATCATGCTGATAATGATAAAAGACTAGGAATAACAGCTAAGCATAACATTTATTAAACCAAAATGACTTACT
>sRNA0248	s0002693	126	223	45	21	40	61	25	31	NC_004350.2	620956	6E+05	-	SMU_661	-17	SMU_662	37	/+/-/+/	AM	TCATTTAAAGTTTATAACTAAAATACAAGGATGAATGACAGTAGCAACAGTCACGCTTTGAGTAATGAGAAACCAGAGCTGCCTCCAGCAGTAGCGATAAACAACGGCTTAACTCTTCTTACTACA
>sRNA0249	s0002694	102	78	19	7	18	19	4	11	NC_004350.2	621052	6E+05	-	SMU_661	79	SMU_662	-35	/+/-/+/	AM	TCCAATAACTTTAATACTATTTAAAACAATTTTCATTTTAATCTCCTCTGATTTATTTATAATATTCTCTGATCATTTAAAGTTTATAACTAAAATACAAGG
>sRNA0250	s0002699	88	61	18	2	8	16	4	13	NC_004350.2	621927	6E+05	-	SMU_662	120	SMU_663	-72	/+/-/+/	AM	TCTGATGGCCATTTAAGATTCGGACTAATTCTAATCCACTATATCCTGTAATACCGACAATCGAAACTTTCATACTTATTCACTTCCT
>sRNA0251	s0002704	116	83	15	8	8	23	8	21	NC_004350.2	623052	6E+05	-	SMU_663	88	SMU_664	-44	/+/-/+/	AM	TGAGAAACCTAGCGGACTGGCAATATTTCCATCAATTATTTTCATATCGTAAACTTTCAACCTTCTAACAACAGCACTCTTGCAAAGTAATATCTTAAAAGAATGACTCTCTTTTA
>sRNA0252	s0002707	33	149	28	20	23	38	18	22	NC_004350.2	624303	6E+05	-	SMU_664	-13	SMU_665	10	/+/-/+/	AM	TTTGCTAATGGCTCAGCTGCTAGGTCCGATAGA
>sRNA0253	s0002711	74	130	29	24	16	31	7	23	NC_004350.2	625070	6E+05	-	SMU_665	-12	SMU_666	75	/+/-/+/	AM	TTATGACGTCACTTTGCTTTAATGAATCTATGTCCTTCTATTCGACTCCAATTCCTTTCTATTAATCTGCCGTG
>sRNA0254	s0002719	115	78	16	4	10	14	23	11	NC_004350.2	626440	6E+05	+	SMU_666	83	SMU_667	-73	/+/+/-/	AM	TAAGTGCTTCTTCTTTTTAATTTATTTGAATAATGATTGGCTTATTTTCCCATGAGATAATCGTCATCATGCATGGCTTCAACAGAACCAAGCAGGTAACCATTACCGACTTGTG
>sRNA0255	s0002722	117	182	34	41	36	27	23	21	NC_004350.2	627592	6E+05	+	SMU_667	152	SMU_668c	295	/-/+/-/	IGR	TATCTTCTTATCACAGCAGCAAGTATTTATTTGGGAGACTTCATCATTTTTTTAGAAATTCTATTTTTGGCGCTTGTTTACCAAATATTTACATGCTTTGCATTAGTAGAAATGGCG
>sRNA0256	s0002727	133	80	17	11	22	13	12	5	NC_004350.2	630099	6E+05	+	SMU_668c	-63	SMU_669c	131	/-/+/-/	AM	TAGGACGATTAATTTCATTGTTTAGGCGAAAGTAAGAGATATCGCCAAGATTTTTTAAACTCATCTATCTAATTCCTTATTATTCTTTGACATAAGAAGGGGGTTGGAAATTTCCCAACCTCTTTCTTTAAAA
>sRNA0257	s0002731	48	90	11	12	18	17	16	16	NC_004350.2	630292	6E+05	+	SMU_668c	130	SMU_669c	23	/-/+/-/	IGR	CCAGTTCATTAGTTGTGGTATAGAAATGCCTCTAGCAATGAACATGTT
>sRNA0258	s0002732	134	140	36	17	33	27	18	9	NC_004350.2	630322	6E+05	+	SMU_668c	160	SMU_669c	-93	/-/+/-/	AM	TCTAGCAATGAACATGTTGCTGATTTTGTTATCGAATTGCTTAAATAATGGTTTTCAATTTAGCAGGCTGAAACCCTGAGAAGACAAGATCGCTTGTTTCAATAACAGGAGCGGCAGTAAATCCTAGACTTTTG

>sRNA0259	s0002747	41	858	127	178	130	161	135	127	NC_004350.2	635984	6E+05	-	SMU_672	171	SMU_673	457	/+/-/+/	IGR	TAAAATTGAAATTTGCAGAGCTCAAAATTTTTTTACGTTGG
>sRNA0260	s0002766	83	79	14	9	24	9	15	8	NC_004350.2	641297	6E+05	-	SMU_676	195	SMU_677	394	/+/-/+/	IGR	TTAGATATTGACAGAAACTGACCTCATGAGCTTAGATAAGCTCCAACTGTTTCCCAAACAATTGGAGCTATCATTTGCAAAGC
>sRNA0261	s0002767	87	104	13	20	29	14	12	16	NC_004350.2	641303	6E+05	+	SMU_676	201	SMU_677	384	/+/+/+/	IGR	CAAATGATAGCTCCAATTGTTTGGGAAACAGTTGGAGCTTATCTAAGCTCATGAGGTCAGTTTCTGTCAATATCTAATCCACTTTGC
>sRNA0262	s0002776	110	192	28	31	38	32	37	26	NC_004350.2	641490	6E+05	+	SMU_676	388	SMU_677	174	/+/+/+/	IGR	AAAATTAAACTGAGGGATTCAGAGTGTAGATAAAGTCTCGATTAGGGCTCGCTTCGCTTGCAAATCAATGCCATTTAAATTGGTATTTGCAGAGTGTCAAAACATATATG
>sRNA0263	s0002777	56	87	18	15	11	13	11	19	NC_004350.2	641498	6E+05	-	SMU_676	396	SMU_677	220	/+/-/+/	IGR	TTTGCAAGCGAAGCGAGCCCTAATCGAGACTTTATCTACACTCTGAATCCCTCAGT
>sRNA0264	s0002807	95	233	48	38	45	37	30	35	NC_004350.2	644901	6E+05	+	SMU_681	42	SMU_682	167	/+/+/+/	IGR	TTTCCCAAAAAAATCAATACTCTTAATTAGAAAATGGAACCTTGGATATCATAAGATAGAAAGAAGTCGTTCAAAAATATGAGCGGCTGTTTTTTA
>sRNA0265	s0002831	45	83	26	18	9	11	11	8	NC_004350.2	653450	7E+05	+	SMU_688	38	SMU_689	164	/+/+/+/	IGR	AAAGGAAACTCCTTTTGTGAGATAAGATTTCTGTATGTAGTGAGA
>sRNA0266	s0002834	53	69	16	12	11	13	8	9	NC_004350.2	653566	7E+05	-	SMU_688	154	SMU_689	40	/+/-/+/	IGR	TATCATGTTTATCAGGACGTCTCAAGATTAAGCATTGTGCTATATATTCCAAT
>sRNA0267	s0002860	109	242	36	53	51	45	33	24	NC_004350.2	661894	7E+05	-	SMU_699	91	SMU_700c	8	/+/-/-/	IGR	CTTAAGTATCAAACGGCCAATACAAGCCATTTTCTTTGTCCATTTATGAAGCATAAAATCTCAAGCATCAAAAAACATCTGAAGACTAACTAGTTTTCAGATGTTTTTT
>sRNA0268	s0002862	97	1197	195	217	234	183	188	180	NC_004350.2	661936	7E+05	+	SMU_699	133	SMU_700c	-22	/+/+/-/	AM	TGCTTGAGATTTTATGCTTCATAAATGGACAAAGAAAATGGCTTGTATTGGCCGTTTGATACTTAAGCTTTGTCTTACAATTCTTTTTTATAAGAGG
>sRNA0269	s0002880	41	156	32	28	33	17	37	9	NC_004350.2	666034	7E+05	+	SMU_706c	-26	SMU_707c	143	/-/+/-/	AM	CAATCCAAGGAGTCCATGTGTGTTCATTTAGTTCATTCATG
>sRNA0270	s0002881	108	1710	267	394	364	254	215	216	NC_004350.2	666062	7E+05	+	SMU_706c	2	SMU_707c	48	/-/+/-/	IGR	TAGTTCATTCATGAAACGAATCACATTCCCACGCTAGTATTAACTAGATCAGGTCCATAGGATTTTGCAAATGCAATCTCAGCTAAGGTTAGCACTCCTTGTGATATT
>sRNA0271	s0002883	83	151	14	11	49	28	23	26	NC_004350.2	666178	7E+05	+	SMU_706c	118	SMU_707c	-43	/-/+/-/	AM	TATTTTTATTATAAAATGAAACTGGCAATTGTGTCAAGTTTATTGGTTGCTTGATCCAAATAATTTTTCATAAGTAGATTTTG
>sRNA0272	s0002885	73	91	16	26	13	13	10	13	NC_004350.2	667019	7E+05	+	SMU_707c	-46	SMU_709	248	/-/+/+/	AM	AAACTAAAGAAAGCTAGAACAAAGACAGGCTTAATTCTTCTTCTCATCTCATTTATTGTAGCGTAAAGTTTTG
>sRNA0273	s0002918	141	1020	179	178	221	181	122	139	NC_004350.2	675254	7E+05	+	SMU_715	136	SMU_716	-66	/+/+/-/	AM	TATCAAAAAAACACCTGATTGAAGGTGCTTCTTTTAGACTGAAGATAAAACTTAGACTGTTTAATGAAAACAGGTTAACGTCTTAGAATTTTTTTAATCGTATTTAAAAGTCTGTATTTTAAGGGATTTGGATAATAATGG
>sRNA0274	s0002930	128	449	101	69	88	85	44	62	NC_004350.2	678633	7E+05	+	SMU_718c	47	SMU_719c	74	/-/+/-/	IGR	AAAAATTAGCTTATTTGTCATGTTGTGTGGGGATTAATTGATAAAGCAAGCGGATAAGGAAAGATAGGAAGCAAAATGTTATCAGAAAATTTCCTTCCTATCTTAATTTTATTTCTAAACGATTAGTC
>sRNA0275	s0002957	119	146	28	26	33	18	27	14	NC_004350.2	685455	7E+05	-	SMU_724	-63	SMU_725c	250	/+/-/-/	AM	AAAACAGGAGAGTGAGGAAAATCAATTTCTACGAAATACCGATTGAGCCCCGCTTTCATTCTTTTTTAGTTTGTAATGATTTTCTGAGTTTAACAGCTTCTTCTGGTTTATCTGTTATA
>sRNA0276	s0002969	126	620	107	86	102	134	81	110	NC_004350.2	688370	7E+05	-	SMU_728	194	SMU_730	-50	/+/-/+/	AM	TTTTTCCCCAACTGTAGCTGTCCAAGCATATTTACCTTTTGGTGTTTTCACGATGATCGGCCTCCTTATAGTATGTTTTGTATGATTTGTATAACTTATTATACCATATAATTTCAAAATAACAAG
>sRNA0277	s0002970	98	169	34	13	27	41	20	34	NC_004350.2	688603	7E+05	-	SMU_730	-54	SMU_731	-41	/+/-/+/	AM	ATAAATTTTCGTAAGACCATTTACTTCTAGAATATATCTCATGTCATTCGTCTTCTTTCTCTTCAAAAGCTACTTCCATCAAGTGTGTGAATGCTCCC
>sRNA0278	s0002978	30	207	43	38	39	28	17	42	NC_004350.2	690101	7E+05	-	SMU_732	58	SMU_734	127	/+/-/+/	IGR	CGTGCAGGGATAACGATTTGGTCTTTTTCA
>sRNA0279	s0002986	111	160	37	28	26	23	24	22	NC_004350.2	690651	7E+05	-	SMU_734	-31	SMU_735	107	/+/-/+/	AM	GAGAGTCTGAAGTCAATCTAATCTTGAACTGCTTCCCTCGATCTGACTTCCTGTATTCAATTTAGAGGGACAGCACAATTCAGTTTCTCTTTTCCGATTTTGACTCTCCTT
>sRNA0280	s0003000	120	114	15	18	16	31	12	22	NC_004350.2	692781	7E+05	-	SMU_737	190	SMU_738	12	/+/-/+/	IGR	AAAAGGCAAGACCAGCCAAAATCAGCGCTGATTTTCTTCTAAAAAGATAGAAAATTGGACTCAAGACGCCACCTGACAATGACCGAACCACACGATCACAAAGAAAATCGGATAGTTGGT
>sRNA0281	s0003004	95	115	7	25	35	15	9	24	NC_004350.2	693027	7E+05	-	SMU_738	-19	SMU_739c	69	/+/-/-/	AM	ACTGCATCAAACTGTTATCATTGTTTTATCTAACGAGGTTGGCACTTCTTGTCCTAACCTCGTTTTGACTGATACCTAAATCTCTTTCCTTCTTT
>sRNA0282	s0003033	149	315	72	78	62	38	35	30	NC_004350.2	700731	7E+05	+	SMU_745	-34	SMU_746c	4	/-/+/-/	AM	CGAGAGACTTTATCTGTCTGTTGTGTACTAATCATGTTTTCTCCTCTTCTTACTTCTCATTATTGTAACACAAAAAAGAAGTACAACAAAGCTGATTTTTTAGAAATGAACCTTGTTAGACTTCTTTTAATTGAGATATTATTCTTAAA
>sRNA0283	s0003058	94	110	18	16	28	13	18	17	NC_004350.2	706569	7E+05	-	SMU_752	67	SMU_753	-72	/+/-/+/	AM	TATCCGACAGTCCTGCCACAACTCCGCAGATCATACTACCTTTTCTTTTCTTATAAAAAGTGGCTTTAGACATACAAACAACTCTCCCTTTTTT
>sRNA0284	s0003061	102	414	98	44	90	64	56	62	NC_004350.2	706905	7E+05	-	SMU_753	16	SMU_754	81	/+/-/+/	IGR	CTAACTTGTCTTTCAAACTATTAGGCAAAGGTTGTAGGGGGCTTCTTTCCTGTTTCTAAGAAGTCCTATTCTAAAATAATTGATTATCTTTAAGTTCACACT
>sRNA0285	s0003074	99	95	11	21	21	9	22	11	NC_004350.2	709642	7E+05	-	SMU_757	-31	SMU_758c	31	/+/-/-/	AM	CCAAAAAAACAAGATAACTGCAATTATTAGTCTCCTAACTTCTATGCAGCAGTCTTGTTTTTAATTATTAATCTTTTTTAGTTTTTTCTTCTGCTTGAT
>sRNA0286	s0003084	142	3294	607	404	742	528	554	459	NC_004350.2	711141	7E+05	-	SMU_759	-83	SMU_761	214	/+/-/+/	AM	GCTGTGCCCTTAAAAAGGAAAACTCTATCACAGCAATCAAAATCTAAACAGTGAAAAATTACTTAACTGTTTGCGGATCAAGCTCATAAAATCCTGTATCTAAACCACGACCAGCTGGATGAAGCTTTCTAATTTGTTCATC
>sRNA0287	s0003088	117	75	10	10	8	18	10	19	NC_004350.2	711358	7E+05	-	SMU_759	134	SMU_761	22	/+/-/+/	IGR	AGATTGTAAGAAGATTTCCAAAGTCAGACTCATCAAAACAATATTGTTGATGGGGCTGACAAGTGCAATCCCTAGAAAAACCTTATGAGAGTGGGACAAAATCAAAAAGGCAGAACT
>sRNA0288	s0003095	104	2372	382	471	479	381	286	373	NC_004350.2	712881	7E+05	-	SMU_761	99	SMU_764	-72	/+/-/+/	AM	TAACCGTAACAAATTCTCCTTGATGATAAGCTTGGGCTGAAAATTCAACCATTTCCTTTCCGACTAAAGACATACACTTGTCCTCCTTCTAAATTGTTTTCATT
>sRNA0289	s0003100	95	127	21	14	16	24	15	37	NC_004350.2	714976	7E+05	-	SMU_765	-38	SMU_766	15	/+/-/+/	AM	AAAAGTTAGATTTTTTCTGTCTAACTTTTGGGGTGCAGTTCAATAAGTCCTTTGTTTTAATTTCTAATCAAATAATCAAAGGCACCTAAAGCCGC
>sRNA0290	s0003120	65	86	12	9	21	11	11	22	NC_004350.2	719325	7E+05	+	SMU_771c	-22	SMU_772	400	/-/+/+/	AM	TAAGAAATATAGTATCGTTTCATGTTGATTATGCTTTCATTAGCAGTTGATTTTTCTGAACTGTA
>sRNA0291	s0003121	107	257	48	74	50	29	25	31	NC_004350.2	719354	7E+05	+	SMU_771c	7	SMU_772	329	/-/+/+/	IGR	TATGCTTTCATTAGCAGTTGATTTTTCTGAACTGTAATTATTGTTATTATAGCATAATGGACTGAAAAATATATTATCAAGATTACTGTATTCAAAAAAATAGATTC
>sRNA0292	s0003152	137	308	39	52	87	66	24	40	NC_004350.2	725259	7E+05	-	SMU_774	108	SMU_775c	62	/+/-/-/	IGR	TATAGGTTGGAAAAATACTGAAATTATAGTAATTAAACGTTTTTTACCCTATCACTGATTTTATGAGAAACGAAAAAGGCAATCGTATGTGCTTCCGCCAAGCCACACACGATTGCCTTATATATTGTCTCATAACA
>sRNA0293	s0003153	22	77	17	6	5	15	18	16	NC_004350.2	725313	7E+05	+	SMU_774	162	SMU_775c	123	/+/+/-/	IGR	ATTGCCTTTTTCGTTTCTCATA
>sRNA0294	s0003158	149	81	13	4	29	11	12	12	NC_004350.2	725415	7E+05	+	SMU_774	264	SMU_775c	-106	/+/+/-/	AM	TTAGATTAGGAATTTCCAGTTTCTTTTATTTGGATAAGCTTGTTATTTTGATGATGTAGTTGTTGAGGATTCTTTTGGTGATTCTTCTGGGTGTAGTTCTTTATAACTTGGTGCCTTGTATAAATCTTGAGTTGATTTGTTACCATTTT
>sRNA0295	s0003165	148	221	47	38	47	28	24	37	NC_004350.2	729515	7E+05	-	SMU_777	-69	SMU_778	-88	/+/-/+/	AM	AAAGCGTAGTTGTGAATAAAAGGTGAGATAGAATGTTTAATTGGTGTCGCGACAACAGCTGCTAAACGTGTATAACCATCAATTTGCATCTAGAACCTCCTTAATTTTACGCATATCTGCCAAAGAAATCTGTCCCGGAGCGCTTTCT
>sRNA0296	s0003211	129	2488	455	381	385	467	331	469	NC_004350.2	740245	7E+05	-	SMU_788	335	SMU_789	57	/+/-/+/	IGR	TTACAGACAATCATACCTTATTACATTTTACTTCTCACTTCAAAGAATAGCTGCCAAAACAACAGACTAATCACTCCGCTTATATTATAGCATAAAATATTATGCAGAAGTTAGGATTATATAATGTAG
>sRNA0297	s0003218	37	747	139	150	141	110	106	101	NC_004350.2	741096	7E+05	+	SMU_789	31	SMU_790	514	/+/+/+/	IGR	TAAGTTATAATAAACAGTTAAGGCGCAGATCTATAGC
>sRNA0298	s0003219	144	107	25	9	15	19	22	17	NC_004350.2	741148	7E+05	+	SMU_789	83	SMU_790	355	/+/+/+/	IGR	GAGTTCAAGGATTTCATAAAAATGAATGTGGAATCTTGATAATGCCTGTGAAGTCAGAATTTGTACTGTATCTCCAATCATAGGTTGATAGGTTAACATGTCTCAATTTTGGCTAACAGGAGGAAGGCAGTTGCTTCATCAAGA
>sRNA0299	s0003220	35	64	9	13	8	11	17	6	NC_004350.2	741229	7E+05	+	SMU_789	164	SMU_790	383	/+/+/+/	IGR	TTAGCCAAAATTGAGACATGTTAACCTATCAACCT
>sRNA0300	s0003227	65	80	13	14	19	16	9	9	NC_004350.2	741418	7E+05	-	SMU_789	353	SMU_790	164	/+/-/+/	IGR	ATAGTATAATAGGAAATGATAGATGACTCCCCCACCCCGATCTGTCAAATGATAATAGACATAGT
>sRNA0301	s0003271	87	69	18	6	8	13	3	21	NC_004350.2	745713	7E+05	-	SMU_799c	90	SMU_800	35	/-/-/+/	IGR	CTAAAGGGCAATAAAATATGTGATTCCAAAGCTTCAACAGTAACCTTTAATGGGAATATAGAATTTATAGGAAACGCTTCCAAAATT
>sRNA0302	s0003272	135	321	62	61	30	48	28	92	NC_004350.2	745798	7E+05	-	SMU_799c	175	SMU_800	-98	/-/-/+/	AM	TTTATATTTATAGCTTATTTTTATCTTATTTATATTATCATGAATTAGTCTAATAAGCAAATAACAAATATAAGTCACGAGACTGCTATCACGATTCAACTATCCGTTCTAGCTCCATCTGTATATATATATACT
>sRNA0303	s0003285	119	4648	845	968	714	704	665	752	NC_004350.2	751066	8E+05	-	SMU_804	61	SMU_805c	77	/+/+/-/	IGR	TAAGGGTGCAAGTCAGTCCACAATAGTGGCAAACATTGACAGTTAGAGATATAAAGAATTGATTTAAAAAGTTCCGACGTAAAAGCTCTCAGTTCGATATTAAGTGAGAGCTTTTACTA
>sRNA0304	s0003287	145	762	117	132	177	122	109	105	NC_004350.2	751199	8E+05	+	SMU_804	194	SMU_805c	-82	/+/+/-/	AM	AAAGTCTTATAGGACTTTGCATAGTTTAGCTAGTGACTTTAGTCACAGATAAACTTAACACGTTAGACATTCAACACTTTATCCAGGAAGTCTTTGAGACGTGGGTGCTGTGGGTTATCAAAGATTTCTTCAGGTGAGCCATCCT
>sRNA0305	s0003313	68	71	22	10	9	15	6	9	NC_004350.2	757683	8E+05	-	SMU_809	200	SMU_811	101	/+/-/+/	IGR	AAATCATGGGACAAGCCTACAGTCCAAAAAGCACTAACCAGACAAGACTGACACCAATTACCTTAACC
>sRNA0306	s0003334	100	194	35	44	32	28	30	25	NC_004350.2	760793	8E+05	-	SMU_815	7	SMU_816	110	/+/-/+/	IGR	ACAGCAATCATGCCTGTCTATCCCAGCATCCAACTCATTAGCATATTATTCTAATGATTGACTATTTCGCACTCCAACTATAAATTGTGAAGGCTTTACT
>sRNA0307	s0003348	146	313	57	46	44	83	47	36	NC_004350.2	763838	8E+05	+	SMU_819	-101	SMU_820	116	/-/+/+/	AM	TAAGGAAGTGATAATGGCGTTAAAAGCAGCCCCCATAACAACAGCTACTGCAAGATCTAGGACGTTACCCTTAAATAAAAAATCTTTCAATTCTTTGATCATTTTAGATTTCCTTTCTGTTCATCAGATTCTTATTATACAGGAAG
>sRNA0308	s0003353	129	150	22	39	28	16	27	18	NC_004350.2	764063	8E+05	-	SMU_819	124	SMU_820	-92	/-/-/+/	AM	TAAGAGTGAAACGGCTGATGCGACAAAAAGAGTTAAAATAATGATTTCATTTTTTCGAACCAGCCCTTTTCGCTGACGTTTTCTGCTCTTCATTTTCTTCCTTTCAGTAAATTAGTGATAATATTATAG
>sRNA0309	s0003363	107	62	12	6	16	9	10	9	NC_004350.2	769046	8E+05	-	SMU_823	25	SMU_824	186	/+/-/+/	IGR	TGGGGGTAGGACTGCAAAATCAATTCTACGAAATCACGATTTTCATATGTAACTTATAGATTAGACTATTTTGTGGGTGATTACGCAATAATCATGTGTGTCTGGCC
>sRNA0310	s0003370	88	62	12	8	7	4	18	13	NC_004350.2	770280	8E+05	-	SMU_824	88	SMU_825	-2	/+/-/+/	AM	CACGAAAGTCTCAAAACCACCATATTTTGCTGGTAAGCCGCGACTTCCTATGATGAATACATGACGCAAACTCTATCCCCCCATTTTA
>sRNA0311	s0003387	134	412	73	75	72	79	53	60	NC_004350.2	782384	8E+05	-	SMU_833	-21	SMU_834	20	/+/-/+/	AM	AATATTATCAAGCAACAAAATTTAGAATCAACAACAATTAAACATGACTTACTAGTAAATGCTAAGCTCTGGTTTTATAAAGCTATTGATTCTAAATAAATGCCTATAGCCTTTATCCTTTTTCCTTAACAATA
>sRNA0312	s0003388	134	193	38	24	31	45	37	18	NC_004350.2	782509	8E+05	-	SMU_833	104	SMU_834	-105	/+/-/+/	AM	CATAAATGTCAGCTTCCGGTAAGACTCGCTTAAAATCAGAGATAACTTTTTGAATAGTAATCTCTTCATTGTAAGCTGGAAGTAATATTGCAATTTTCTCTGTCATAGTATAAATTCTCACTATTAATATTATC
>sRNA0313	s0003392	44	164	25	25	34	21	31	28	NC_004350.2	783470	8E+05	-	SMU_834	-5	SMU_835	96	/+/-/+/	AM	TAATCGCCACAAATAATACCCAACCCCAGCCCTAAAAACTATTT
>sRNA0314	s0003394	99	261	56	49	47	33	37	39	NC_004350.2	783513	8E+05	-	SMU_834	38	SMU_835	-2	/+/-/+/	AM	CAATTAGACCAATCTCCCTCTTTTTTATCTACATAAAATTCTAAAAATTTAAAATAGTGGCTAATAAGCTGAGTTAGATAAAAATTTGAACATTTTTAT
>sRNA0315	s0003404	147	264	47	59	41	59	34	24	NC_004350.2	788215	8E+05	-	SMU_837	-6	SMU_838	-7	/+/-/+/	AM	TTAGTCATAAAAATAAAGACTCCTTGTATTAATCATAAATTTACTTTATCACAAAACACTTGGACTGACAATTTTTTAATCTGAAAAAACAGAGCAAGCTAACCTATTGCCCAGCAAGCAGGTAAGCCTGTTCTGTTTTCTTAAAAG
>sRNA0316	s0003435	134	658	116	126	108	150	81	77	NC_004350.2	796904	8E+05	+	SMU_845	43	SMU_847c	-65	/+/+/-/	AM	TATAAAAGTTGACTATATGCACATCCTGTGCAACCGCACGAAAATCGCTTTTAAGCAGCTTTCGTAGCTCACGATTTAGGCGAGTCTTCACGGAGGAATTTCCTCAAACAAAAACTTATAGGAGGTGCATCATG
>sRNA0317	s0003443	50	224	38	45	28	61	20	32	NC_004350.2	798033	8E+05	-	SMU_849	22	SMU_850	243	/+/-/+/	IGR	AAATACAGACTGTTCTTATTCCCTGTAAGAAAAACTTTAAAAAATTTCCT
>sRNA0318	s0003466	94	3202	593	465	654	507	524	459	NC_004350.2	799879	8E+05	-	SMU_851	367	SMU_852	193	/+/-/+/	IGR	TAAATCAAGATACAAAGAGCGTTAAGCGGATAAAAGCAAAATAGGGAGAGTAGGATGTAAAATCATTAAGATTCTAGGAGGATTCCTCTTTTTG
>sRNA0319	s0003468	140	196	53	37	15	56	17	18	NC_004350.2	799987	8E+05	+	SMU_851	475	SMU_852	39	/+/+/+/	IGR	TGGATTCCTGAAATTATCCACTTGATAATTTACCACCTATTGGTGGTTCAAACAGTCCCCAGACTGTTTGAAGTTGAAAAGAAAATCTCAAAAGAAGACTTTTTCTTCAATCTGAATCACGCGCAATGTGTGATTTTTTT
>sRNA0320	s0003484	64	79	14	15	9	15	9	17	NC_004350.2	803265	8E+05	-	SMU_855	71	SMU_856	89	/+/-/+/	IGR	TCTTTGTCGCTCCTTGCCTGCCTCACGGGACAGTGTTAAAGGAATATTCATTTCTACTATTATA
>sRNA0321	s0003505	91	247	42	45	45	31	47	37	NC_004350.2	807443	8E+05	-	SMU_859	86	SMU_860	4	/+/-/+/	IGR	TAAGATATTAAGGGCGTTAAGCGGACATAGCAAAAATAGGAAACCAGCCGACGACGCTTGCGTCTAGGATGTGTTTACTTTTTTGCAAAGT
>sRNA0322	**s0003508**	29	96	18	17	21	19	9	12	NC_004350.2	810698	8E+05	-	SMU_860	-18	SMU_862	257	/+/-/+/	AM	TTTATTCTGATTATATAGCTTGTGTAATA
>sRNA0323	s0003517	88	176	32	23	23	41	26	31	NC_004350.2	812265	8E+05	-	SMU_862	-52	SMU_863	-29	/+/-/+/	AM	AAGATGAATGAGTTCTTTTCTATCACTCACTGCACTCACCTCTTTTCATTAGCCTTACTAGAAGGTTTTTCAATCTTCTTGCCTTCTT

>sRNA0324	s0003520	125	100	20	14	15	21	14	16	NC_004350.2	812996	8E+05	-	SMU_863	-34	SMU_864	-80	/+/-/+/	AM	TATGATAATCCCCAGCATCGTCAGAAAGGAACGCATTTTATGACTAAGAATAGAAGAAAGAGCAAATTTCCAATTTTCCATAAATTCCCCCTATTCTAATTCAGCCATTTGCTGGCTGTCTTCTG
>sRNA0325	s0003547	128	61	7	12	10	11	9	12	NC_004350.2	826470	8E+05	+	SMU_875c	4	SMU_876	814	/-/+/-/	IGR	GCGTAAAATAGTCTTTGGGTTACGGAAGAGTTGATAACAGATGAAGCGATAACCTTTCTGAGTATCATGGAAGAGGTCTGTAACCTTTTGAGCGAGGTTTGAATTAAACCGCTTATATTCAGGTTTCC
>sRNA0326	s0003550	36	120	14	12	19	17	30	28	NC_004350.2	826719	8E+05	-	SMU_875c	253	SMU_876	657	/-/-/-/	IGR	AATGCCATTTTAAAGATCTTAAGGCCTCTCCTCAGA
>sRNA0327	s0003553	133	167	35	22	26	31	29	24	NC_004350.2	826780	8E+05	+	SMU_875c	314	SMU_876	499	/-/+/-/	IGR	TCTTCTAGTGACATCTCATCTAGATTTCTCGTTTTAGGACGACCAGTGGACTTAGTGCCTCGTAAATCTTGTGTGAGTAGGGCTGGGTTTTCGCGGTATTTCCTTGTCCAAGTTAGTCCCTGATTATCTGATT
>sRNA0328	s0003558	128	120	35	14	20	22	6	23	NC_004350.2	827033	8E+05	-	SMU_875c	567	SMU_876	251	/-/-/-/	IGR	CATTATGACGCAACCTCTAATCAGCAAAAAAAGAGAACTCTATGAAAAGAACCACTTATAAAAGAACCACTTATAATGGACAGTATAAAAAAGTTATATACTGTTCTTATAAGGAGGTTCTTTTCATA
>sRNA0329	s0003563	38	100	29	4	13	17	6	31	NC_004350.2	827341	8E+05	+	SMU_875c	875	SMU_876	33	/-/+/-/	IGR	GTATCGCAAACGTTTGCAAAACTGTTCCTTTCTAATAA
>sRNA0330	s0003564	25	86	17	9	8	18	4	30	NC_004350.2	827355	8E+05	-	SMU_875c	889	SMU_876	32	/-/-/-/	IGR	TTTATTAGAAAGGAACAGTTTTGCA
>sRNA0331	s0003605	64	60	7	11	13	6	9	14	NC_004350.2	844378	8E+05	-	SMU_889	-40	SMU_890	181	/+/-/+/	AM	AAATGGTACTGTCCCTTGTCTTTTTATTTTGACGTTTCATTTTTCCTCAGCGCATACGAATCTG
>sRNA0332	s0003646	133	133	17	26	25	30	16	19	NC_004350.2	858901	9E+05	-	SMU_902	69	SMU_905	256	/+/-/+/	IGR	ACAGCTCAGCAACAGCCAGAAAAACTGGAGCAAATCCTCCAGTTCCAACAATCTGACTATAATCTTTCAAGACAAAATAAACTACTCTAGGAATAGATTGATAGTAGCTAGCCATCTAAGTAGGAATCAAAAC
>sRNA0333	s0003647	44	89	9	10	14	26	16	14	NC_004350.2	859063	9E+05	-	SMU_902	231	SMU_905	183	/+/-/+/	IGR	CAAAGATAGACTTTAAGAGCATACTGGATAGACCTTGGTCTTAA
>sRNA0334	s0003664	92	221	31	39	45	30	33	43	NC_004350.2	863841	9E+05	-	SMU_909	-3	SMU_910	132	/+/-/+/	AM	AAAGGCAGCATTGATTATCTTTTTTACCATCACAACAACCGTCGTAAAATAATTTAAAAGGGCTATAAATTACATCTTTTAACACAATTTAT
>sRNA0335	s0003668	133	66	9	12	12	11	2	20	NC_004350.2	863972	9E+05	-	SMU_909	128	SMU_910	-40	/+/-/+/	AM	TTTTTAACCTTGTACATTTTGTAACGTCGTTTGGTTTCCATAAAATATCCTCCTTTATCAGTTCAACATTATAACCTTATAATGCTACAAAAGCGATAACATATACGTTACAAACCCTTTTCTTTATTTTTCT
>sRNA0336	s0003674	28	239	33	63	50	28	35	30	NC_004350.2	868457	9E+05	-	SMU_910	5	SMU_911c	143	/+/-/-/	IGR	AAATTTATTGATCACGTTAAGCTATGTC
>sRNA0337	s0003691	128	237	33	60	57	29	29	29	NC_004350.2	869387	9E+05	-	SMU_911c	251	SMU_913	258	/-/-/+/	IGR	TGACTTTATCAGCGGGTGCCCTTTATATCTTGATTCAACTGAATACGGACTGCGGGACCTCTGCAAAAAGACAAACATACCTTAGACGCAAGCGTCGTCGGTTAGTTTCCTATTTTGCTCTTGTCCGC
>sRNA0338	s0003705	118	235	35	28	79	28	35	30	NC_004350.2	871554	9E+05	+	SMU_914c	-62	SMU_915c	54	/-/+/-/	AM	TAGGATAACAATCACTCTACGAAGAGTTGGTTTAAGAAAAGTTTTCAATTCATTTTTCATCATAGTGACCTTCTGTCTTTATTATAATATAAAGGAGAAAGGGGGTCTATAGCTTTAT
>sRNA0339	s0003717	109	98	19	15	30	14	14	6	NC_004350.2	873974	9E+05	+	SMU_919c	-64	SMU_921	508	/-/+/+/	AM	CAAAAAAGGCAGGTAGTTGAATCTTGACCACCGCTAAAGACGACAAGAGCTGATTGACGTTTCATAAAAACTCCTTTAAATTTCGTTAAGGATAATTTTGAGCAGTAAA
>sRNA0340	s0003720	99	4823	1058	430	769	986	760	820	NC_004350.2	874071	9E+05	-	SMU_919c	33	SMU_921	421	/-/-/+/	IGR	GAATTACATAAGGATGTTTGGGAGAAGCCATCTATAAAAAAAAACCAGCCTATTATTTGGCAGCTCCTATTTTTATATTAGGAGCTTTTTTACTGCTCAA
>sRNA0341	s0003721	29	363	44	28	113	64	51	63	NC_004350.2	874095	9E+05	+	SMU_919c	57	SMU_921	467	/-/+/+/	IGR	ATAAAAATAGGAGCTGCCAAATAATAGGC
>sRNA0342	s0003722	68	364	58	48	127	49	26	56	NC_004350.2	874112	9E+05	+	SMU_919c	74	SMU_921	411	/-/+/+/	IGR	CAAATAATAGGCTGGTTTTTTTTATAGATGGCTTCTCCCAAACATCCTTATGTAATTCTTCAATATGG
>sRNA0343	s0003743	137	901	150	190	175	149	114	123	NC_004350.2	879242	9E+05	-	SMU_924	-14	SMU_925	15	/+/-/+/	AM	TAATAACATAAAGATATTCTTAGTATAACAGAAAATATCCTCTAACGGAGTCAAAAATCCCAAATGGTAGCAATTTTGTCCTAAACGGTAAAAAGACACAGAAATTGTGCCTTTTTACTAACTTATTTCACTGCTGC
>sRNA0344	s0003747	133	144	33	12	34	24	28	13	NC_004350.2	879866	9E+05	-	SMU_925	3	SMU_926	93	/+/-/+/	IGR	TGAGGTTACAAACAAACTAAATAACTGAAGTCAGCAAGGCATCGTTTTGATTTTTGAAGAGTATTAACTAGCTGCCATAACCACAAAAAGCCTAGCATTATCTGCATGGTAGGCTTTTTGTGGTTTAATCAGT
>sRNA0345	s0003763	116	272	46	69	37	34	49	37	NC_004350.2	888548	9E+05	-	SMU_936	143	SMU_937	-51	/+/-/+/	AM	TAATGATAGCAATATTTGCATAAGACTTCACTTTTACAGATTTTACGGTCACTTTGGAACTTCCCTTCTTAAGTTTATTCATTATGATTGCATTATCATTTTAATATAAAAATGAG
>sRNA0346	s0003770	130	428	63	55	112	72	82	44	NC_004350.2	891454	9E+05	-	SMU_939	-95	SMU_940c	49	/+/-/-/	AM	ATAACCACCTTAATGACACCTTTAAAACAAGCCATTATTGTCTCTGTCCTTCTCTTAAGCGCCCATATAAAAGATAGTCAACATTTCGAAGTTCTGCAATCGTTTGACAGTTAAGGGCGCACATGATCAG
>sRNA0347	s0003773	135	74	31	0	13	10	8	12	NC_004350.2	892181	9E+05	+	SMU_940c	-107	SMU_941c	-58	/-/+/-/	AM	GAGCAGAACAAGCATAATAGCTGCACCAACAGCATGTGTCACACTGTTAGCAACCTCTTCGCCAAAACTCAATTGTTTACTTAGTTTCAGAGTTTGTGTTGGACTCATTGATATCACTTCTTTCTAGTTCTAAGA
>sRNA0348	s0003775	100	433	90	65	102	81	43	52	NC_004350.2	892553	9E+05	+	SMU_941c	-45	SMU_942	218	/-/+/-/	AM	TGATTGGCTTTTCAATATGACCATGTTTGACATAATTATTAATCATAGAAGCTGTTAGTCCTTTGTCTTTAGAAGCAGCAGCATCTGTGGTCTGATTAAC
>sRNA0349	s0003776	70	532	85	87	126	109	48	77	NC_004350.2	892624	9E+05	+	SMU_941c	26	SMU_942	177	/-/+/-/	IGR	GAAGCAGCAGCATCTGTGGTCTGATTAACATAGAGCAGGACTTGGTCTAAATATAAATCAAGTTCAGGTA
>sRNA0350	s0003802	114	144	37	28	25	21	24	9	NC_004350.2	899233	9E+05	-	SMU_949	-58	SMU_950	-39	/+/-/+/	AM	GAAGAATAGAAGCATTGTGGGTATTTAAAAATTCACTCATCACATCTCCATCTCCTTAGGAAGCTGTCTCAAGAATAGGTTTGTCGGTCCCTTCTACCGCTTTCTTAGTAATGC
>sRNA0351	s0003805	142	547	109	74	115	110	68	71	NC_004350.2	899809	9E+05	-	SMU_950	-91	SMU_951	279	/+/-/+/	AM	AATCACACCCTTCATCTAATCAACTTTTTAACTATTTCACCTTGTGAAATTTAAAGAGACTCTAAAATGGCATCCCAAGATGTATCCAAACCTGTCCGATCAACTGACGAAAAGATGATAAAAGCATCATTGGAATCAAAAT
>sRNA0352	s0003806	82	141	16	30	31	33	17	14	NC_004350.2	899951	9E+05	-	SMU_950	51	SMU_951	197	/+/-/+/	IGR	TTTTTTATCCACTCTAGGCAAAGCATTCACAGATTTATTTTATCTAAAAAAGCAAAAAGGTATGACTCACTATGTTTATAGG
>sRNA0353	s0003828	80	163	33	14	34	43	23	16	NC_004350.2	907833	9E+05	-	SMU_956	234	SMU_957	82	/-/-/+/	IGR	TTTTTCAGTTTACGTTTTGTGCCTCGGCAGGAATATTATGAGCACGCTCCCCTGCTGTCTTAGGTCAGTTTTTCAAAACC
>sRNA0354	s0003829	150	188	53	35	19	39	10	32	NC_004350.2	907902	9E+05	-	SMU_956	303	SMU_957	-57	/-/-/+/	AM	TTTTCTCAGCAACAGCATCAACTAATTCTGCTTTTTTAGCAATACTTGCTTCACTCATCAGTGTTTAACCTCCGTTTTTATTTTTGGCTGAATGCAATAAAAAATCGCACCCAAACCCAGACACGAAAGTACAATACGTTTTTTCAGTTT
>sRNA0355	s0003834	64	2023	371	355	394	345	298	260	NC_004350.2	908923	9E+05	-	SMU_960	-8	SMU_961	212	/+/-/+/	AM	ACTAAGAAAACCTTAGCGGACTTTATTGTTTGAATAGCTTTAAAACAAGACTCTCTTATTTAAG
>sRNA0356	s0003840	142	264	45	32	59	45	65	18	NC_004350.2	909670	9E+05	-	SMU_961	-79	SMU_962	-41	/+/-/+/	AM	AGTATCTAACCAATCAATAAATTCTCCCGAAAAATAAGCCATAATAGCCCCCTTTTCATTTTCTATTTCACATACTGCTGTAACTCTGGGTTAATTGGTGTGTCTGCCATATTATTGGCATAATTGCAAAGCGAGGCTAAAC
>sRNA0357	s0003843	132	382	76	59	56	86	50	55	NC_004350.2	910740	9E+05	-	SMU_962	-97	SMU_963c	558	/+/-/-/	AM	CATATCAAAAGAGGACAAGCAAGAGTTCAAGGGGCTAAACAGTTTCCAAAATATGTCGAAGCTGAACTGCACTTGGGGAAACTATCGGCAGAAAAGCCCCTTCATTCCAACGGCGAATAAAACTTGATGTTG
>sRNA0358	s0003848	106	71	6	23	13	15	12	2	NC_004350.2	911083	9E+05	+	SMU_962	246	SMU_963c	241	/+/+/-/	IGR	AAAGCAATCATTTAGGGTGACTTTGTTGTCCTAGAGACCCATCGTCTAAAAAATAAAGACGATGGGAATACCCACCAGTTGCCGCTGTGGTCACAGTGTCGTTTCC
>sRNA0359	s0003881	133	94	28	9	9	10	10	28	NC_004350.2	923170	9E+05	-	SMU_976	65	SMU_977	51	/+/-/+/	IGR	TGACCTCATCACCGGGCTTTTTTTGAAAACCTAGCCCCTTCCCCATAACAATAATCTCTTCGTTGTTGATACCAAGTATCTGAACGACATTGTTATTAAACACTTTTTCAATTTTCATAGAACTTTCCTTAGT
>sRNA0360	s0003882	147	68	20	11	13	13	0	11	NC_004350.2	923259	9E+05	-	SMU_976	154	SMU_977	-52	/+/-/+/	AM	TCAATTTCTTCGCTATCAAGATCAACATAAACACGCGATAACTCTCCGACCATGTCTGTATCCGTATTTTGTAAAATAAATCGTTTTTAGATTAAATCTTGATTGACCTCATCACCGGGCTTTTTTTGAAAACCTAGCCCCTTCCCC
>sRNA0361	s0003905	87	236	28	41	54	46	31	36	NC_004350.2	929293	9E+05	-	SMU_983	138	SMU_984	-39	/-/-/+/	AM	TAATCTCTTTAAATGTCATTTTTTGCTGTCTGCTAATCATTCTTGCAAATAACTACATATTTCTTATGTCTTATACTTAAAATTATA
>sRNA0362	s0003912	80	86	18	5	14	15	18	16	NC_004350.2	931373	9E+05	-	SMU_985	-37	SMU_986c	46	/+/-/-/	AM	TTCACCCTAAACTCTCTTTAAATTTTTAATCAACATCAATAATCATTCATAGAGGCTGGCACCATTAGTTTCAATAACCT
>sRNA0363	s0003914	108	120	24	20	16	22	20	18	NC_004350.2	931465	9E+05	+	SMU_985	55	SMU_986c	-74	/+/+/-/	AM	TCTAAACTTTTTTGTTGGCAGTAAAATATCTATTTAGCAAGGAGGAATAACGTCATCAAAAAAGGAAAAGGGCTTATAAGTAGAGGTGGTAGAACCCAAAATAGAAGT
>sRNA0364	s0003916	60	61	9	2	20	8	8	14	NC_004350.2	931988	9E+05	+	SMU_986c	-25	SMU_987	315	/-/+/+/	AM	TTTGAATATTGAATTCTTTTTGGCATAAACTCACTCCAATCAATTATTTTATATAATATT
>sRNA0365	s0004005	96	97	26	13	17	8	19	14	NC_004350.2	960193	1E+06	+	SMU_1005	88	SMU_1006	82	/+/+/+/	IGR	TTGGCTGTTATCAAAAAAAACAGAGCCAAACTAAAATATTGTTTGGCTTCTATTAAATTAATGTCAATGCTTACAATTTTGTAAGCTACGATTCTT
>sRNA0366	s0004006	31	116	21	7	18	28	16	26	NC_004350.2	960201	1E+06	-	SMU_1005	96	SMU_1006	139	/+/-/+/	IGR	ATATTTTAGTTTGGCTCTGTTTTTTTTGATA
>sRNA0367	s0004023	94	106	20	21	17	15	18	15	NC_004350.2	964914	1E+06	+	SMU_1009	124	SMU_1010	-66	/+/+/-/	AM	TTTTGCTGTTTATTGAGGGGATTCTAATTAATAATGAATGACATTATCTGCTTTTTGAATTTTTTTAATAAGCGGCTGTGCTTCAGGACTTAGG
>sRNA0368	s0004024	74	340	59	54	72	66	46	43	NC_004350.2	965947	1E+06	+	SMU_1010	-43	SMU_1011	146	/-/+/-/	AM	TTATCAGAAAAATAAATTTTTGAAATGGGATGAGAAGTTGACATGGTTTACTCCTTTAAAATAAATTGCAGATA
>sRNA0369	s0004036	86	151	45	21	16	25	20	24	NC_004350.2	967877	1E+06	-	SMU_1012	126	SMU_1013	19	/-/-/-/	IGR	AAAGAGGGTTAGGGAGCAAGTTTATTTCCTAGCTCTTTTACTTGTAATATTAGTAAGAGAGTGGGGCAGACTAGACTATTGCTGTG
>sRNA0370	s0004038	112	129	17	27	18	22	14	31	NC_004350.2	967961	1E+06	+	SMU_1012	210	SMU_1013	-91	/-/+/-/	AM	TTAGGATAAGTGTGATTTTCTTATTGTGGAATATAAAATGGTAGACGTCCCACGATAAGAATTGTCACCACAAAGATGATAAAGACAACCAGTGCATATTTAGCAGACTCTC
>sRNA0371	s0004095	138	1432	234	265	259	275	223	176	NC_004350.2	982629	1E+06	-	SMU_1032	106	SMU_1034	207	/+/-/-/	IGR	ATACTATCTTTTATTTTTTAAATAAACATATCACAAATTTCCCACTATCATTTTGAATATTCTTAAATGCTCGTAAAGCCTTGCTGTATAGACTTTCGGGCGTTTTTTCTTTAGGAAGATACTTCACGTTTCCTTGTG
>sRNA0372	s0004096	81	914	155	143	142	200	113	161	NC_004350.2	982694	1E+06	+	SMU_1032	171	SMU_1034	199	/+/+/-/	IGR	CGAGCATTTAAGAATATTCAAAATGATAGTGGGAAATTTGTGATATGTTTATTTAAAAAATAAAAGATAGTATCTTGATGT
>sRNA0373	s0004099	133	985	155	163	203	180	146	138	NC_004350.2	982797	1E+06	+	SMU_1032	274	SMU_1034	44	/+/+/-/	IGR	TATCTAAATGTAAAGATATTCTTATTTATATTAAAGATGGATAAAGTAGTATATAGATATATATGTTTTCATGCTCCTGCCTCAAAAGTTCCCCAAACCTTTTTATATAGCTATACTAACTATGAGGTATGGG
>sRNA0374	s0004120	46	348	61	20	76	58	48	85	NC_004350.2	989121	1E+06	+	SMU_1040	-41	SMU_1041	157	/-/+/+/	AM	AAAACCCGCCGAAGCACCTGTAATCAATACTGTTTCTGCCATTTTG
>sRNA0375	s0004131	83	541	113	102	85	98	65	78	NC_004350.2	991685	1E+06	+	SMU_1042	12	SMU_1043	7	/+/+/-/	IGR	TATCTCATATGTAAAAAAGAGACTTAGATTTTTTATTCTAGTCTCTAGTCTAAGACAAATTCCGTTATTTTGGAGTTTGTCTT
>sRNA0376	s0004193	130	186	40	20	53	24	34	15	NC_004350.2	1E+06	1E+06	+	SMU_1063	-103	SMU_1064	247	/-/+/-/	AM	TCAAGTATTTCCGTTTTACTTTTTTGTTCTTTAATCATCTCAAGTGCTGCTTTTTGTTTTTTACCAAAAATTTTGGTGAGATGTTTTATTTCAAGTTTATTCAACTTTATTCCTCTTTTCTAATCATAAT
>sRNA0377	s0004202	76	416	69	44	85	65	63	90	NC_004350.2	1E+06	1E+06	+	SMU_1065	-36	SMU_1066	165	/-/+/+/	AM	TGATTTGATCATGAATGCGAATATAAGCCGGTAACATCGTCTCTCCTTAACCACAAAATATTTTTTCGTACTAATG
>sRNA0378	s0004216	129	67	13	18	11	10	4	11	NC_004350.2	1E+06	1E+06	+	SMU_1069	-79	SMU_1070	-59	/-/+/-/	AM	AAAAGGCACAAGGTATAAATGCAGGTTTTATAATCAATAGCAATTTCCCTACTTAAAAAACCTTTCCATCTACTCTTCATCTTGACCTCTTTCCTGTAAAATCGTTCGAATTCTGTTAGCATACTTTCG
>sRNA0379	s0004265	58	62	17	8	5	12	2	18	NC_004350.2	1E+06	1E+06	+	SMU_1083	-24	SMU_1084	-40	/-/+/-/	AM	TAAGTGCTTCTTCAATTTTATCCATCGTCAACCACAACCATTCTATCCTTGCCAAAAC
>sRNA0380	s0004271	108	63	12	4	21	16	4	6	NC_004350.2	1E+06	1E+06	+	SMU_1085	10	SMU_1086	-57	/-/+/-/	AM	TAACCTTTCTTTGGACTTGATACATCAATAAGTCATTCCTGTCAAAAATATCAGGCTTTGATTGGAGGATGGAAGTAATGTCTTCGACAGACAGGAATATAAGTTTCA
>sRNA0381	s0004274	91	89	23	7	13	15	11	20	NC_004350.2	1E+06	1E+06	+	SMU_1086	-81	SMU_1087	141	/-/+/+/	AM	TATGAGCAACCTTTAAAATTTCGATTGTCTTACCTGAATTCATGGTCCCATATTTATAATATAACTGCGCCAAGACTGTCATCCCCATCTC
>sRNA0382	s0004279	141	355	53	38	103	60	56	45	NC_004350.2	1E+06	1E+06	-	SMU_1087	20	SMU_1088	-78	/-/+/+/	AM	CAAGTTCTTTAGCATTGTCGGCTTCAACCATAAGATCAATCGTTGTCCCCATCAGATGTAAAGTTTGTTGTGCCTGCAAATTTTTCCTACCTCCTTTAGAGCTTTCTGATTATATGATAGCGTTTTCTGAAAATAAAATCA
>sRNA0383	s0004287	114	140	25	24	29	33	8	21	NC_004350.2	1E+06	1E+06	+	SMU_1090	10	SMU_1091	119	/+/+/+/	IGR	TATTTTAACAATAAAAGGTTAGGACAAAAGTCCTGCCTTTTTCAAATATATTAGGCATAGTAGCTTGAAGTCAGACTGCTTAACTTTCTATTTTTGTCGATATGCTCTTAATTG
>sRNA0384	s0004318	70	72	18	15	25	7	2	5	NC_004350.2	1E+06	1E+06	+	SMU_1100	128	SMU_1102	188	/-/+/-/	IGR	AAGTGGCAGAAACAGTGAAGCTTCTGCCACTTTTATCACGGCGGAAAGTTCTCGATTAGGGCTTGCTTCG
>sRNA0385	s0004338	129	267	59	25	73	45	21	44	NC_004350.2	1E+06	1E+06	-	SMU_1106	173	SMU_1107	256	/-/-/-/	IGR	AGTGTAGGTAGCAAAAAAGGTTGATAAAATGACAGAATGGTTTAAAGGGACGGTTATTATAAATCTTTTATTTTAGGAATAAAAAGCAATCAAATTGAAAATACAATTTAGTGCTATCGTATGATAGTA
>sRNA0386	s0004348	63	357	63	59	77	60	62	36	NC_004350.2	1E+06	1E+06	+	SMU_1106	398	SMU_1107	97	/-/+/-/	IGR	AGATAAAAAATACGACTTAAATCTCTTCTTTCAATTGTGATAGAGCTCATTTGCTTGATCATC
>sRNA0387	s0004349	104	112	21	23	17	18	20	13	NC_004350.2	1E+06	1E+06	-	SMU_1106	421	SMU_1107	33	/-/-/-/	IGR	CAATCTAGTAGCCAAGTATGTTAAAGATTTGGAAACAGTGAGAAGGTTTTTTGAAAAATACTTTGATGATCAAGCAAATGAGCTCTATCACAATTGAAAGAAGA
>sRNA0388	s0004353	35	132	34	15	35	26	10	12	NC_004350.2	1E+06	1E+06	+	SMU_1106	511	SMU_1107	12	/-/+/-/	IGR	TGGCTACTAGATTGGGTGTTTGATTTTCTTTGGTT

>sRNA0389	s0004364	122	683	120	110	127	161	93	72	NC_004350.2	1E+06	1E+06	-	SMU_1108	30	SMU_1109	64	/-/-/-/	IGR	CAGGAAGTAAGTGATTTTATGCCAGAACTTTAAAAGGGATCCGATTTTGCCTTCAAATACAGTCGAACGAATTAGAAATATTATTTGATTTAAAATAATATTGTTATTATTTATCTAAGATT
>sRNA0390	s0004372	146	205	33	49	53	19	17	34	NC_004350.2	1E+06	1E+06	+	SMU_1109	119	SMU_1111	16	/-/+/-/	IGR	TAACTCATTAGAATGGGATACCTTATTAGAACAGACAGCTATGATTGCAGCGCAATAACCTATAAAAGAGTCTAATCGTGAAGATTACGTATAAAAAATCGGATTGTTAGCCTTAACCTACTCAAAACAAGACTAAATAACTTAGG
>sRNA0391	s0004377	93	100	19	20	17	15	14	15	NC_004350.2	1E+06	1E+06	+	SMU_1111	-17	SMU_1112	145	/-/+/-/	AM	ACTTAAAGAATACCCCATAACATAGTCTGGCTGAACATAAGATTCATCAATAACCTTCTGAACAGAATCGTTGCTGCTAGCAGCAACGAAACT
>sRNA0392	s0004388	36	544	96	90	89	110	89	70	NC_004350.2	1E+06	1E+06	+	SMU_1112	72	SMU_1113	111	/-/+/-/	IGR	CCATCACACCAGCGTACGTACTATTAGACATATAGC
>sRNA0393	s0004390	130	170	35	16	29	29	18	43	NC_004350.2	1E+06	1E+06	+	SMU_1112	117	SMU_1113	-28	/-/+/-/	AM	ACTAACTTCTCATGATTCCTTGTTATTACGTTTGCAATGCTCATGAGACCTCACAGAATAAGTCGTACCTAAAAGAAACTCGTTAGAGCGAGTTTCTTATTTTAAAATGATATTTGATTATAGGACTGCC
>sRNA0394	s0004408	57	714	141	128	125	117	102	101	NC_004350.2	1E+06	1E+06	+	SMU_1117	-51	SMU_1118	235	/-/+/-/	AM	TAATGGCAGCTGTACCTGCATGGTTAGCTCCAACAATAACGATTTTACTCATAAGCA
>sRNA0395	s0004437	132	62	14	11	9	6	5	17	NC_004350.2	1E+06	1E+06	+	SMU_1124	0	SMU_1125	-22	/-/+/-/	AM	TCACCAAGATACCAAGAACGCTATAAAAAGCAAGTGAATGTTAAAAGCTTGCTAAAATCCCAATTTCTGATGCAAACTATCCAACTAAATTCAAGCTTTCTACTTGTGTTCAATCTCCTTCTTTCACACTTC
>sRNA0396	s0004446	89	237	26	72	46	36	31	26	NC_004350.2	1E+06	1E+06	+	SMU_1127	34	SMU_1128	-32	/+/+/-/	AM	AAAAATTAATAACGTTCTTCTAATCATACTTAACCGAATCTGTTTTTTATAACACTTTATTTATTAGCAAGCAGGCGAGCTTCAAACAT
>sRNA0397	s0004450	55	213	43	53	28	32	36	21	NC_004350.2	1E+06	1E+06	+	SMU_1129	-38	SMU_1131	35	/-/+/-/	AM	TAGACTCAAATCGTCTTCTACTAATAATAACTTTATCATATTTTACCCCTTTTTT
>sRNA0398	s0004466	74	128	19	40	17	20	11	21	NC_004350.2	1E+06	1E+06	-	SMU_1131	235	SMU_1132	93	/-/-/-/	IGR	TTTTATCAAAAGCCTGTAGGCATACCCACTACAGATACTAAAGTGTCTGAATGTCTTAAATAGTAATATTCAAT
>sRNA0399	s0004473	89	68	12	11	2	11	10	22	NC_004350.2	1E+06	1E+06	+	SMU_1132	-40	SMU_1133	-7	/-/+/-/	AM	TCAGGTACAAACTTTTCAATAAGGTGCTCTACAGTTTCCATGTATCTCCTCAATTATTCAAAATTCTAAGACTATATTATATCATAATT
>sRNA0400	s0004477	86	95	14	7	15	19	20	20	NC_004350.2	1E+06	1E+06	+	SMU_1134	-39	SMU_1135	-34	/-/+/-/	AM	AAACAGAAAGATCGTTTACTTTTAAAATAGGTTCTGTCATCTCTTTCCTTTCTATCCAAAGTGTCCAGATACATAGTCACTTGTCG
>sRNA0401	s0004483	118	64	9	13	18	9	6	9	NC_004350.2	1E+06	1E+06	+	SMU_1137	-1	SMU_1138	-43	/-/+/-/	AM	ATGATTCCTCCACACATCAAGATAAAAGAACTTAAACAAGAAAAATAGAAATCTTATTCTCTTCTCTATTCAATTTACTTAGCAATTACAGTGCCATCTGCACGCTTAACCACTTTCA
>sRNA0402	s0004524	111	61	16	8	15	11	4	7	NC_004350.2	1E+06	1E+06	+	SMU_1153	181	SMU_1154	128	/-/+/-/	IGR	TAAACATCAGGAATATATCGTCCATTCATTTTTAGCCAGTGTAGACTATACTCAAAATACCTTATTTTCCCTACTGTAGTTCCCATAAAACCACTATTAAAGAAGGTATAC
>sRNA0403	s0004550	100	382	66	100	66	56	44	50	NC_004350.2	1E+06	1E+06	+	SMU_1160	-57	SMU_1161	31	/-/+/-/	AM	TACGAACATTAATTTTTTCCAAAATATTAAATATACTTTCAAGACTGTCAAATATCATATTTACCTCTTTTATTCATAAGCACTATCAAAACGGCAAATC
>sRNA0404	s0004575	105	466	95	65	80	86	71	69	NC_004350.2	1E+06	1E+06	+	SMU_1170	10	SMU_1171	83	/-/+/-/	IGR	TATTCGTTTTTTTAATTTAAAGAGGATAGAGGCATGGATTTGGATAGCGTCCACTTCCTTCCACTCCCATTATAGACCACTATTCTCACCCTCTTATTATACCAA
>sRNA0405	s0004593	63	303	55	50	62	32	31	73	NC_004350.2	1E+06	1E+06	+	SMU_1173	93	SMU_1174	126	/-/+/-/	IGR	TTAATTAGTAAACTGTTTTCCGCTTAACGCAAGTGTTGATTGAAATTTCGCAACTTGTTTTTT
>sRNA0406	s0004609	63	92	8	24	11	17	24	8	NC_004350.2	1E+06	1E+06	-	SMU_1176	-43	SMU_1177	104	/+/-/-/	AM	ATTGTTTACTATATTGTAATTAAAGAATAGGCGAGACTTGATGGTAAATAATCTGCCATCTCT
>sRNA0407	s0004614	28	71	10	16	11	12	9	13	NC_004350.2	1E+06	1E+06	+	SMU_1176	103	SMU_1177	-7	/+/+/-/	AM	TTAAGAAAGAGTGATTGTGCTTACCTCT
>sRNA0408	s0004622	114	127	25	31	25	19	10	17	NC_004350.2	1E+06	1E+06	+	SMU_1179	-56	SMU_1180	134	/-/+/-/	AM	TAAAGTAATCAGCGCCCCATCTAAAAGACTCGGTAAAACTTCTGATATATAAGACATACTTGCTCCTCAAAAAATAATCGTTTTTATTATATCAAATTTTCAGAAAATTTTATA
>sRNA0409	s0004624	38	90	15	13	29	13	7	13	NC_004350.2	1E+06	1E+06	+	SMU_1179	110	SMU_1180	44	/-/+/-/	IGR	TGTATCACCAAAAAAGAACAAGAATGAACCACCTTCAT
>sRNA0410	s0004631	120	291	41	46	61	62	37	44	NC_004350.2	1E+06	1E+06	+	SMU_1180	-45	SMU_1182	101	/-/+/-/	AM	CATAGACATACTCTGACTGACATTTAGGACAATTAGGTAAAGACATATAAACTCCTTTTATTTTGATTACCATTAACAGTTTACCTGAGTTTTTATCAGTTGGCAAGTCTTATCACAACA
>sRNA0411	s0004677	143	2287	458	234	515	379	340	361	NC_004350.2	1E+06	1E+06	-	SMU_1192	155	SMU_1193	-119	/-/-/+/	AM	TAAATCTCTAACTGTGGCTAGTTGTTGGCCGGGTTTGATTTCTTGACTGATAATTTGCATTTTGATATGATTGGCAATTTGAAGATAAATAGGGGATTTCTCATTAAATTTCCAAGCCATTTTTTCTCCTTTCTAAAGTGCAG
>sRNA0412	s0004690	107	621	79	134	141	93	90	84	NC_004350.2	1E+06	1E+06	+	SMU_1197	58	SMU_t37	133	/+/+/-/	IGR	TAAAAGCAAAAACGGTAATAAAAAATATTTCTAAACTTCTGTTTAGATAATCTCAACCGAAAGGCAGTAGTTAGCTTATGCTTCTCTTGTCACATAGTTGATAAACA
>sRNA0413	s0004693	118	82	12	21	30	4	9	6	NC_004350.2	1E+06	1E+06	+	SMU_1197	218	SMU_t37	-38	/+/+/-/	AM	ATATTAACTAATAATAAGTCCGCAAAAATCGGGTATCAAAACTACTTTTTGTAAAAGCACCGCTTTCATCTTTACCTAATCCACCTTGAGGGAATCGAACCCCCATCTCAAGAACCGG
>sRNA0414	s0004695	148	637	126	114	145	96	67	89	NC_004350.2	1E+06	1E+06	+	SMU_t37	-16	SMU_1200	7	/-/+/-/	AM	CATTACACTAAAGGTGGGTAACACAATAATTATACCATAGTTTCATTAATTTAGCTGTAAATGTATGAAAATTTCCTTGAAATATAAAAACCTTCTAAATCTTAAAAAAGATTTAGAAGGGAATAAAATTCAATTTCTACCCATTGAT
>sRNA0415	s0004754	105	135	35	19	22	29	19	11	NC_004350.2	1E+06	1E+06	+	SMU_1214	-80	SMU_1215	-14	/-/+/-/	AM	TTTACCATCAAGCAAAACATCTAATACCCGATCAAGACCAGACTTAGGATCAAGAACACGGCCATTTTTTATAAGTAACATAACATCTCCTTATTTAAGCCAATC
>sRNA0416	s0004757	134	635	122	102	112	130	86	83	NC_004350.2	1E+06	1E+06	+	SMU_1216	-84	SMU_1217	101	/-/+/-/	AM	TAAGAACTGAAATACCTAAGGTATATGGAATGCCTGTCAAAACAAAGGATAAACTTTTGATGATCAGATGAAAATTAATAAACATTACATCTATCCTTATCTTTCTATCTAGGGCTGAAATAGCTGAAAAGCAT
>sRNA0417	s0004759	136	72	6	12	12	8	10	24	NC_004350.2	1E+06	1E+06	+	SMU_1216	43	SMU_1217	-28	/-/+/-/	AM	AAAGCATCACGCACCTATCAAACCTATTGTAACAAATTTTCAAACAGCATTCCAGTTACAAAAAAAGACAAAACTAAGGCGATCTCTTGAAAAGAGATCGCCTGCTATTAATCAACGTCAGAAATATCAATTTTTT
>sRNA0418	s0004781	32	83	0	21	15	17	15	15	NC_004350.2	1E+06	1E+06	+	SMU_1220	254	SMU_1221	47	/-/+/-/	IGR	AATGGCACTTTTAGATTATTACTCAAATAAGA
>sRNA0419	s0004790	125	2204	302	322	504	300	558	218	NC_004350.2	1E+06	1E+06	-	SMU_1224	15	SMU_1225	114	/-/-/+/	IGR	AAAAAATGAATAACCTTTAATTTAGTCCAGAGAGGCTGTCAAGGGAAATCAAGATTAACAGGCAGAGGTACAATCTGCCCACTTTTTTGTGTAACCTTGCCAGTTAGTTGGTAAGGTTTTTTTTC
>sRNA0420	s0004791	99	149	31	19	23	25	19	32	NC_004350.2	1E+06	1E+06	+	SMU_1224	24	SMU_1225	131	/-/+/+/	IGR	CCTTACCAACTAACTGGCAAGGTTACACAAAAAAGTGGGCAGATTGTACCTCTGCCTGTTAATCTTGATTTCCCTTGACAGCCTCTCTGGACTAAATTA
>sRNA0421	s0004797	89	273	44	55	64	38	33	39	NC_004350.2	1E+06	1E+06	+	SMU_1225	18	SMU_1226	-40	/+/+/-/	AM	GCTGCAACTGTTGCCTTTTTTATTATAAACAAAATTGATGAATATTTGCTAATTCTGATAGATGCGCTTAATGAGTTCATTCACTTTGT
>sRNA0422	s0004808	128	142	24	14	30	22	22	30	NC_004350.2	1E+06	1E+06	+	SMU_1228	-58	SMU_1229	-59	/-/+/-/	AM	CAAGCTAAGTAGGCACCGGGTGCCTCAAATGTTTCGTGAAGGATAAAATGAATTCTCATACTGACTCCTTATAATTCTACTAAAATAGCTTTTACCAGTCCCTTAAAATCTTCTTTTATCTGCTGTGT
>sRNA0423	s0004819	29	76	12	12	14	10	18	10	NC_004350.2	1E+06	1E+06	+	SMU_1231	5	SMU_1232	76	/-/+/-/	IGR	TCTGTGAAAAAGAGGGCTCCCTTTCGAGC
>sRNA0424	s0004820	71	225	35	34	41	47	38	30	NC_004350.2	1E+06	1E+06	+	SMU_1231	26	SMU_1232	13	/-/+/-/	IGR	TTTCGAGCCAAGTCTTGAAGCCACCAATCAGGCAGTTCTTGGTAATCTTTCTCGTACTCCTCTTGTGAAAA
>sRNA0425	s0004821	90	133	26	27	27	18	18	17	NC_004350.2	1E+06	1E+06	+	SMU_1231	93	SMU_1232	-73	/-/+/-/	AM	AAAAGAGGGAAAAATACTAGACAAAGGGATAATGGTCTGCAAAGAATTGTTCAAACCGAGAACTGATGTTCGCACGAGGACAATCCGACT
>sRNA0426	s0004847	129	87	8	27	20	4	16	12	NC_004350.2	1E+06	1E+06	+	SMU_1238	-21	SMU_1239	-34	/-/+/-/	AM	TAATTTCTTCAAGACTTGTCATAATAACCTCTTTCTCGTTATTAATTGGATAAGACCGTTACACAAATAATATTCGTTGAACTTTCTTGTCTCGCTATTTGATCAATTCATAAATGGCTTCTGCATAAA
>sRNA0427	s0004860	83	175	25	25	54	31	21	19	NC_004350.2	1E+06	1E+06	-	SMU_1241	77	SMU_1243	-41	/-/-/+/	AM	GAGTTCATAGTTGGAGACACGTTTTGATAAAAACTGAGACATTCTTTTTCCTTTCTTTAAAAATACGTCGTTATTTTACCAAA
>sRNA0428	s0004864	82	67	5	2	18	16	13	13	NC_004350.2	1E+06	1E+06	+	SMU_1243	68	SMU_1245	-32	/+/+/-/	AM	TCTTAGAATCAGTAAATCTATAAAAAAACTGAGTCCATTATGAACAGTCTCAGCTTTTTCGTACTAATAAGGCAAGGAGTTG
>sRNA0429	s0004869	98	635	120	84	144	88	100	99	NC_004350.2	1E+06	1E+06	+	SMU_1245	290	SMU_1246	-35	/-/+/-/	AM	TATATATTCCAAGAAATAAAAACTATGGATCGAAAAAGTTTGTCAATGTTTTCTACATCATCCTAATCATTAAAAACGCAATCTGTGTCACTGATTAA
>sRNA0430	s0004900	118	102	18	23	27	11	15	8	NC_004350.2	1E+06	1E+06	+	SMU_1253	-44	SMU_1254	16	/-/+/+/	AM	AAAAGATGTTAACTCTTCACTTGTTTCTGAAAAATGAACGATCATCACCGCTACTCCTTTTTCTTTCATTATACAATAAAACAGCCATTTTTAATGCATTTTTTGTGCTATACTAATT
>sRNA0431	s0004907	135	95	14	12	43	12	7	7	NC_004350.2	1E+06	1E+06	+	SMU_1255	-68	SMU_1256	145	/-/+/-/	AM	TAAATGAATAAAAGCTGAATTGACAAAAGAAGTGCTGACATAGGAAACACCTTCAAAAGAAACCGTCACCTCTTCTCCTGCTCTCAAAGCATCTTCAACAAGAGTTAAGATTTTTAATCCAGAAGCATTATCTGA
>sRNA0432	s0004912	146	252	39	53	45	49	34	32	NC_004350.2	1E+06	1E+06	+	SMU_1256	-78	SMU_1257	-74	/-/+/-/	AM	CAGTGGCATTATCAGATAAGTCTGATATGCTGTAGTTATACTTAGCAACAATTTGGTCAACTGTTTCTTTTTCACTCATAAAGTTCCTCCTCTAAAGTTTCTCTTGTGTTGTTATTTGGTAATATCAGATTGATGTTTTCCATATC
>sRNA0433	s0004927	91	155	22	38	33	18	19	25	NC_004350.2	1E+06	1E+06	-	SMU_1259	200	SMU_1260	64	/+/-/-/	IGR	AAAATATAGTGATACAGTTGCACGATCTTTTGAAATGCCTCTGTATCTAATTATCCAACCTTTTGGATGTTTCTTGCCCTATAATCAAAGG
>sRNA0434	s0004935	109	86	14	15	9	18	14	16	NC_004350.2	1E+06	1E+06	+	SMU_1262	52	SMU_1263	-46	/-/+/-/	AM	TAACTAATTAAATTTCAAACTTTCAGACCGATCTATTCCTTTCTATAAATGCTTTTCTAGTCCTACTCAGGATTGTAAATCTCATTAAAAAAACAAGAATAAGCTCCTG
>sRNA0435	s0004943	35	120	11	18	21	38	10	22	NC_004350.2	1E+06	1E+06	+	SMU_1265	34	SMU_1266	372	/-/+/-/	IGR	AGAAAATAGGAAACTGACGACGTGACTTTGTCACT
>sRNA0436	s0004949	137	65	9	16	6	16	4	14	NC_004350.2	1E+06	1E+06	+	SMU_1265	157	SMU_1266	147	/-/+/-/	IGR	TAAGCGAGCTTTTAGCTCGAACTCATTTCCTTTCTCTTTCTTAATTATCTAGTCAGAAATAACGTGGTCATTCCTAGACTTTAGCTCGGGTTCAGTTCAGTTCGAAAAATCTAACTGAACTTTCCTTTCTATATAGT
>sRNA0437	s0004970	47	162	29	27	26	19	38	23	NC_004350.2	1E+06	1E+06	+	SMU_1273	40	SMU_1276	1088	/-/+/-/	IGR	AAAAACTTGCAAGGACGCTGACACACACGTGGTTCCACCTTCATTCA
>sRNA0438	s0004971	73	68	25	11	8	7	11	6	NC_004350.2	1E+06	1E+06	+	SMU_1273	135	SMU_1276	967	/-/+/-/	IGR	TAACGTGAATAACCCGTCGAGCACTACTTTCCTACGATTTGGCACCCGCACTCAAGAATGTGTTATCTAAGCT
>sRNA0439	s0004975	67	88	17	13	12	12	13	21	NC_004350.2	1E+06	1E+06	-	SMU_1273	399	SMU_1276	709	/-/-/-/	IGR	CTTGGGACAATAGTGCCCCAGCTCAAACAAAAAAGTAACGGTTTTGATACCGTTGCTTTTTTGAATA
>sRNA0440	s0005006	121	108	24	12	27	16	12	17	NC_004350.2	1E+06	1E+06	-	SMU_1280	121	SMU_1282	-102	/-/-/+/	AM	TTTGCTGGATAGTTAATTTACTAAAATCTTCTTCTAAAATGAGTTGGATGACCGCTGTTTCAATGTCGCGTTTACTCTTAAAAACGCGGATATCTTGTGCCATATAATTGGCTCCTTTTTG
>sRNA0441	s0005023	65	202	30	44	21	43	41	23	NC_004350.2	1E+06	1E+06	+	SMU_1288	102	SMU_1289	23	/-/+/-/	IGR	CTTGATTGTAAAGGAAAATATTTTAACAGTATGTGTCTTATTTTCTGAGAATGCTGCGTTTTCTC
>sRNA0442	s0005048	100	97	15	23	18	10	13	18	NC_004350.2	1E+06	1E+06	+	SMU_1292	237	SMU_1293	86	/-/+/-/	IGR	AATGATTTGAATTAGAATCTCGTGGAAGTTTACACAAAGCTTTATAATCAGCCAACTGACGATAAGCACTGTATACTTATCAGTAAATCTAACTACTGTT
>sRNA0443	s0005055	114	69	7	9	15	9	7	22	NC_004350.2	1E+06	1E+06	+	SMU_1293	-5	SMU_1294	52	/-/+/-/	AM	CTTCAATCTGCATCACCTCATATCTAAAAGTAGACCTATTAACACTGAAAGAATTCGCTTTCTTTTTCATTATACTCTTTTTCTCGTGAAAATTCAGTTATAAAACAAAAAAGC
>sRNA0444	s0005072	139	92	24	15	13	18	11	11	NC_004350.2	1E+06	1E+06	-	SMU_1297	53	SMU_1298	-94	/+/-/+/	AM	TTTGACGTCTTAGTTGAACCGCTGAGGAATTGGTAACCCGTTGAAGTATCCATAAAGACAACTGGACGGTATTCTGGATGAATATCTTTTTTCATTTTTAAAAATTTCCTTTCTGCCATAGCTCTTTTCGAGCCATAGT
>sRNA0445	s0005091	115	267	29	70	45	47	49	27	NC_004350.2	1E+06	1E+06	+	SMU_1302	95	SMU_1303	18	/-/+/-/	IGR	ATAAAAAAGAAGCTGAAAAACTTACTATACTGATCCCAAGAATTGGACAGAAAACATCTAACTTGAAAATCATATAAAGTTTAGCTTCTCTATTTTTTAGGTGTTCATACTTGGA
>sRNA0446	s0005092	119	154	42	10	39	19	18	26	NC_004350.2	1E+06	1E+06	-	SMU_1302	95	SMU_1303	14	/-/-/-/	IGR	TTTTTCCAAGTATGAACACCTAAAAAATAGAGAAGCTAAACTTTATATGATTTTCAAGTTAGATGTTTTCTGTCCAATTCTTGGGATCAGTATAGTAAGTTTTTCAGCTTCTTTTTTAT
>sRNA0447	s0005097	101	192	26	22	34	35	26	49	NC_004350.2	1E+06	1E+06	+	SMU_1303	153	SMU_1304	110	/-/+/-/	IGR	AAAGCGAGATGAAAATAGGAAACTGACGCCGTGTGACTTGCACACAAGGAAGTTTATCTTTTTCACGACGCTTTTAGGGCGTGTTCAGTTAGTAAGATACA
>sRNA0448	s0005146	92	272	51	74	45	40	21	41	NC_004350.2	1E+06	1E+06	-	SMU_1314	97	SMU_1315	24	/+/-/-/	IGR	TAAAAAATCGGCTAAATATAAGTTTACATTTGAGTATTATCAAAAAATACGGTTAAATGTCGTTTAACCGTATTTTTAATAGGCTTATTCGA
>sRNA0449	s0005157	25	125	26	31	18	20	13	17	NC_004350.2	1E+06	1E+06	+	SMU_1317	413	SMU_1319	394	/-/+/-/	IGR	TAAAACGAATAAAAAGATACTTTTT
>sRNA0450	s0005167	93	959	233	94	131	220	115	166	NC_004350.2	1E+06	1E+06	+	SMU_1319	-26	SMU_1321	124	/-/+/-/	AM	AGCATGTCCGTAAACATTAAAATACATTTCTCGGCCCAATTGACCAGACCAGTGACTTCTTCTCTCAAAATGCATAGTTAAGATACCAAGAGC
>sRNA0451	s0005168	72	95	21	4	16	21	15	18	NC_004350.2	1E+06	1E+06	+	SMU_1319	52	SMU_1321	67	/-/+/-/	IGR	TAAGATACCAAGAGCGAACCAAAAAGTACTTGAAATTAAAAAACTGTTTAAGAAATCAGAATTTGTTAAATA
>sRNA0452	s0005169	130	432	93	51	92	65	74	57	NC_004350.2	1E+06	1E+06	+	SMU_1319	104	SMU_1321	-43	/-/+/-/	AM	AAATCAGAATTTGTTAAATAATTTAGTCCCTTTCAAGCTTCCTGACCGCATTTATCCTTTCAATATCATTATTTAATTCTCAAAGATTAGATTTTAGCATGAGCAAAACAGATAATGTCACGCATTTCAT
>sRNA0453	s0005186	42	66	8	11	16	15	7	9	NC_004350.2	1E+06	1E+06	+	SMU_1323	43	SMU_1324	39	/+/+/-/	IGR	TTAGTAAATATCAAAAAACGGATGATTTAGAATCCATTCTAT

>sRNA0454	s0005193	120	149	32	29	18	22	25	23	NC_004350.2	1E+06	1E+06	+	SMU_1326	-75	SMU_1327	124	/-/+/-/	AM	TGAGCTTAAGTTCGTTTAACTCTTGTGATGTCTTTTGGGCAGCAATATTGTCATCCCAGAAGTCTGGTTCTGTCATTTGATTTTCTAAAAGAGCGATCTCTTCTTCTAAGCGATCTAAGT
>sRNA0455	s0005194	64	113	17	14	21	20	23	18	NC_004350.2	1E+06	1E+06	+	SMU_1326	31	SMU_1327	74	/-/+/-/	IGR	TAAGCGATCTAAGTCAAAGAGACCTCCGAAAGCTAGTCAACTTCTCTTTATTTTCTACTATTTT
>sRNA0456	s0005204	124	111	25	13	18	26	16	13	NC_004350.2	1E+06	1E+06	-	SMU_1327c	373	SMU_1329	264	/-/-/-/	IGR	TCTTAGTTTCTTTTTAAGCTTCAAACTGAAGGTCTTAAATATTGTGCCTCTGAGACCTAATATTTTGTAGCAGAACCTGTTTTTTTAATTAAGGCTGGAACAAAAGTGCCCCAGCCTTTTTATT
>sRNA0457	s0005218	112	1822	329	267	381	274	262	309	NC_004350.2	1E+06	1E+06	+	SMU_1332c	94	SMU_1334	577	/-/+/-/	IGR	TTCAATCCGATTAAATCTGTGGTATTCTTAAGATGTTCCACATGAGTCTTTCTAATGTAAGTTTGGTCGCTTTTCATTATAAATCATATGGGACTTTTTTCATACACTAAAA
>sRNA0458	s0005223	143	3602	549	716	720	562	549	506	NC_004350.2	1E+06	1E+06	+	SMU_1332c	663	SMU_1334	-23	/-/+/-/	AM	CTAACCTCCAAAATTAGACAAGATTTCTATGAGGATGATTGCGTTCTGTATTGCATAAATATTTGATACGGATAATTCTTATTATATCACTTTTAACAAATAACTATGAAAATTATCTATCAGTATAAATTGAATCTTTTTAT
>sRNA0459	s0005227	95	227	43	19	44	45	45	31	NC_004350.2	1E+06	1E+06	+	SMU_1334	5	SMU_1335	-71	/-/+/-/	AM	TTTTACCTATTACCAGTTTATTTTTAAGTATTGCCAACCTGTGGTAAATCATATTTACTGTAGGTCTTTTCTGTACAGCTATTCCATTCATTTAG
>sRNA0460	s0005240	52	62	14	6	12	9	13	8	NC_004350.2	1E+06	1E+06	+	SMU_1339	3	SMU_1340	44	/-/+/-/	IGR	AAAGCGTTTCTTCGATTTTATTTTGAGGTTTACTTGTAGTCATATCGTATAT
>sRNA0461	s0005241	26	98	35	7	12	16	23	5	NC_004350.2	1E+06	1E+06	-	SMU_1339	43	SMU_1340	30	/-/-/-/	IGR	TGAAGAAAGGGATTATATACGATATG
>sRNA0462	s0005253	103	102	17	18	13	26	13	15	NC_004350.2	1E+06	1E+06	+	SMU_1344c	-37	SMU_1345	-50	/-/+/-/	AM	CCAGAAAATAATGCTACTAATCTCATAATCTGCTCCATTCTATTAGACTAAATTAACTCAATAGTCTTATTTGCTCTTCAATATACTCTGCTAACAATTTAGG
>sRNA0463	s0005269	132	985	160	174	201	159	148	143	NC_004350.2	1E+06	1E+06	-	SMU_1349	-96	SMU_1351	148	/+/-/+/	AM	AAAAATGATTTCTAAGAGCTTTGTCTTCAGTCTAATTATTGGTTTCTCTTCTTTTTATAGGTAATTTGAAATTGAGTTTGATACATCAATAGAAATTTTTTTGTAAAAATATCAGGAGATTCTGGTATTGGC
>sRNA0464	s0005296	130	66	20	7	6	8	15	10	NC_004350.2	1E+06	1E+06	-	SMU_1358	43	SMU_1359	-36	/+/-/+/	AM	ATAGGTTAATTTTTTTCTCAATTCCTGCTAATTTCATATTAAAATTATGTTATTAGTCAGATTGAAAATAAACTTTTTTTGGGGTGCTTTTCTATTAATACTCAGAAGGATCAATTAAACAAACCTTAAA
>sRNA0465	s0005301	37	76	10	4	22	20	11	9	NC_004350.2	1E+06	1E+06	+	SMU_1359	30	SMU_1360	-31	/+/+/-/	AM	CAAAACTAATAAGGCGAACTAAGAAAGCCCCTACTTT
>sRNA0466	s0005309	120	664	107	110	108	139	95	105	NC_004350.2	1E+06	1E+06	+	SMU_1361c	424	SMU_1363	98	/-/+/-/	IGR	TAAGGATATAACCCCATCTAAACTTCAAATGCTGGTCTTTTCCCTGACACCCAATCTAGACACCCTCCCCTTCAAATCATAACCACCCGTCTAACGGGTGGTTTGGACGAGGGCTATAAG
>sRNA0467	s0005312	33	229	22	33	57	38	42	37	NC_004350.2	1E+06	1E+06	+	SMU_1361c	563	SMU_1363	46	/-/+/-/	IGR	CCTAAAGACGCTGGCTTTCACTTTGTTCAAGCC
>sRNA0468	s0003547	128	61	7	12	10	11	9	12	NC_004350.2	826470	8E+05	+	SMU_875c	4	SMU_876	814	/-/+/-/	IGR	GCGTAAAATAGTCTTTGGGTTACGGAAGAGTTGATAACAGATGAAGCGATAACCTTTCTGAGTATCATGGAAGAGGTCTGTAACCTTTTGAGCGAGGTTTGAATTAAACCGCTTATATTCAGGTTTCC
>sRNA0469	s0003550	36	120	14	12	19	17	30	28	NC_004350.2	826719	8E+05	-	SMU_875c	253	SMU_876	657	/-/-/-/	IGR	AATGCCATTTTAAAGATCTTAAGGCCTCTCCTCAGA
>sRNA0470	s0003553	133	167	35	22	26	31	29	24	NC_004350.2	826780	8E+05	+	SMU_875c	314	SMU_876	499	/-/+/-/	IGR	TCTTCTAGTGACATCTCATCTAGATTTCTCGTTTTAGGACGACCAGTGGACTTAGTGCCTCGTAAATCTTGTGTGAGTAGGGCTGGGTTTTCGCGGTATTTCCTTGTCCAAGTTAGTCCCTGATTATCTGATT
>sRNA0471	s0005357	35	82	15	10	15	9	19	14	NC_004350.2	1E+06	1E+06	-	SMU_1370c	655	SMU_1372	73	/-/-/-/	IGR	TTACCTTCTTGCAATACGATCTTGCCCCTCTTGAA
>sRNA0472	s0005377	126	317	43	36	57	73	54	54	NC_004350.2	1E+06	1E+06	-	SMU_1373c	191	SMU_1374	68	/-/-/+/	IGR	AATATGTCCTTTCTAGTCAGGACGATTGAGGCACAGAAGAGTAGTAGCTTAGACTCTTCTCTCACATCAAACTTGATATTATCCACCCTAGTGAACTCTCCCACGTTGTCAGTAAGAATAACAGGA
>sRNA0473	s0005378	62	218	26	40	33	50	36	33	NC_004350.2	1E+06	1E+06	+	SMU_1373c	251	SMU_1374	72	/-/+/+/	IGR	TGTGAGAGAAGAGTCTAAGCTACTACTCTTCTGTGCCTCAATCGTCCTGACTAGAAAGGACA
>sRNA0474	s0005380	36	202	28	42	48	28	22	34	NC_004350.2	1E+06	1E+06	-	SMU_1373c	314	SMU_1374	35	/-/-/+/	IGR	CAAATTATCCCGAATGAGGGTGTGGCTTTTCTCAAT
>sRNA0475	s0005389	127	135	19	31	26	16	17	26	NC_004350.2	1E+06	1E+06	+	SMU_1374	167	SMU_1375	38	/+/+/-/	IGR	AAATTCTGTTGATTTGACACCTTTTTCCCATAACTAATACTCACTTAAAATCAAAAAGCAACCTCTCGTTCATTCTCTACTGACAATTGATTCCGCTTCTTCCAGACAGGCTTTAGATCACTTATTG
>sRNA0476	s0001824	42	103	15	12	12	18	13	33	NC_004350.2	407614	4E+05	+	SMU_437c	15	SMU_438c	84	/-/+/-/	IGR	AAAAGTTAGATTTTTTCTGTCTAACTTTTGGGGTGCAGTTCA
>sRNA0477	s0005404	123	721	136	84	171	78	141	111	NC_004350.2	1E+06	1E+06	-	SMU_1380	-51	SMU_1381	38	/+/-/-/	AM	CTATCTAGAAAAAAAATAAGAGAGGCCTTATGATGCTTTAGTGTACTGACCCCAAAAAGTTAGACAAAAAATTTAAGTAAAGGATTTCGTTCTGTACTGTACAGGACTAAGTCCTTTTAGTTTT
>sRNA0478	s0005407	124	92	13	25	23	7	10	14	NC_004350.2	1E+06	1E+06	+	SMU_1381	-87	SMU_1382	-34	/-/+/-/	AM	GAAATTGCTTAGGCAAAATTTGATCGGTATCAATATTATCATTCATTAAAGGAACCGTTGTTCCTGTATAAATTGTAAATTCTTCCATATTAAACTTGTGCCTCCGGTAATTGACGAACATCTA
>sRNA0479	s0005430	61	66	9	19	11	6	10	11	NC_004350.2	1E+06	1E+06	+	SMU_1388	4	SMU_1389	129	/+/+/-/	IGR	CAGAGTTGGTGATTGCTCTGTTTTCATTTTTTTGATAGATTTGTTCAAAAGCCGCCTATTT
>sRNA0480	s0005432	106	280	39	32	75	42	43	49	NC_004350.2	1E+06	1E+06	+	SMU_1388	79	SMU_1389	9	/+/+/-/	IGR	TAAGCAGTAAGGGGAACGGCTGCTGTTAGCGGCTCGACAAAGACTAACGAGCAAAAAAGAAGGCAATCAATAGAAGAGAAATAGATTTACGAGTAAGCTTAATAGC
>sRNA0481	s0005435	110	160	39	25	27	28	23	18	NC_004350.2	1E+06	1E+06	+	SMU_1389	-91	SMU_1390	301	/-/+/+/	AM	TAAAAAGCAGTTTCAATAGTAGCTTTTAATGCTGAAAAATAAGAATTGTTTTTACGCATTGAATCTTCTGAAAAATGTTGGCGTGTAACCATATTAATTCCTCCCAAATA
>sRNA0482	s0005442	39	63	20	13	7	13	3	7	NC_004350.2	1E+06	1E+06	+	SMU_1389	116	SMU_1390	165	/-/+/+/	IGR	TAGCTAGTGAGCAGGTTCTAGGAGTTTAAGAGAGTGAAA
>sRNA0483	s0005448	120	1302	238	152	319	194	173	226	NC_004350.2	1E+06	1E+06	-	SMU_1390	-74	SMU_1391	20	/+/-/-/	AM	AAATGAAATCTCACTTTTTTGTGTTTTTAGGTTTAATTGAAAAAATCAGGACATGTTCAAAGAGGAATTGTCAGTCACTTGCCTCTTTTGACCAATCATTTGCTTGATTCCCAATAGAAT
>sRNA0484	s0005451	102	138	17	24	30	24	18	25	NC_004350.2	1E+06	1E+06	+	SMU_1391c	-59	SMU_1392	38	/-/+/-/	AM	TAAAATACTAAAAACAGCCTTCTTTTTTGATTTCCTACGGCTAGCACGACGTCTTGGCATGAGAATCCCTCCATTTTCTTATTCTACTCTTCTTATTTTACA
>sRNA0485	s0005474	80	66	17	2	20	11	14	2	NC_004350.2	1E+06	1E+06	-	SMU_1395c	262	SMU_1396	54	/-/-/-/	IGR	ATTGTATAGTAGTTGATTAGATTAGACTCAAGCCTTTAACAGTCTACTGAACTGTTGAAGGCTTGAGCAGCAGTTCTTGT
>sRNA0486	s0005475	76	111	11	19	16	32	13	20	NC_004350.2	1E+06	1E+06	+	SMU_1395c	291	SMU_1396	29	/-/+/-/	IGR	TTCAGTAGACTGTTAAAGGCTTGAGTCTAATCTAATCAACTACTATACAATAAAAAGGGCGGAACACTTTTTTGTC
>sRNA0487	s0005481	115	127	24	17	24	19	28	15	NC_004350.2	1E+06	1E+06	+	SMU_1397c	-101	SMU_1398	341	/-/+/+/	AM	TTTGGGACTTAAACTATAATTCACTCTGAGAGCTCGCAATGTCAACTTCTCCACTTCTGCATCCCCTTTCAACTTACCATTTGATTCCCATACAAACTGCATAGGAATCATTACC
>sRNA0488	s0005485	132	630	148	90	129	90	90	83	NC_004350.2	1E+06	1E+06	+	SMU_1397c	29	SMU_1398	194	/-/+/+/	IGR	TATTCATATGAAATACTTTTAATTTCAAAAGGCAGATTAATTAAGAAAAAGGGAACCTAATCAATCATCTTAACCACCACAGATTTTAAGATTTTGAAATTAATAAGGTGAAGATTATATTGTAGTTAAAAT
>sRNA0489	s0005494	88	215	31	35	52	24	42	31	NC_004350.2	1E+06	1E+06	+	SMU_1398	90	SMU_1399	91	/+/+/+/	IGR	TAAATCAGTAACTAACATGGCTCTAAAACTCAAATGTAGTAGTTACTTTCTTTTCACATGTTTTGGAACCATTCGAAACAACACAGCT
>sRNA0490	s0005498	147	309	56	49	52	80	38	34	NC_004350.2	1E+06	1E+06	-	SMU_1399	-123	SMU_1400	58	/+/-/+/	AM	AATCAAAACCTTTTAAAAAAGAATCATTTTGAAGCTGAAATCTGGCTGAGATGAATGGCGCGATTACGAAATGTCGAGACAAAAATTGGTCCACGAGGTTTTAGAGCTGTGTTGTTTCGAATGGTTCCAAAACAAAAGGAAACGCCA
>sRNA0491	s0005515	136	12331	2178	1631	1960	2462	1771	2329	NC_004350.2	1E+06	1E+06	-	SMU_1405c	46	SMU_1406	200	/-/-/-/	IGR	GCTTACAGTAAAGCAAATAAAAAAGCACCGAATCGGTGCGCACTTTTTCAAGTTGTGTACGGACTGGATTAAAAATCACTGCCTTATTTTAACTTGCTGTGTTGTTTCGAATGATTCCAACACAATTATTGTATAA
>sRNA0492	s0005528	119	712	133	84	173	76	139	107	NC_004350.2	1E+06	1E+06	+	SMU_1406c	49	SMU_1407	-51	/-/+/-/	AM	AATGCATCTTGCGATTAAAAAAAGTCATATCTTATATGTACTGACCCCAAAAAGTTAGACAAAAAATTTAAGTAAAGGATTTCGTTCTGTACTGTACAGGACTAAGTCCTTTTAGTTTT
>sRNA0493	s0005532	53	109	17	12	16	18	13	33	NC_004350.2	1E+06	1E+06	+	SMU_1408c	15	SMU_1409	28	/-/+/-/	IGR	AAAAGTTAGATTTTTTCTGTCTAACTTTTGGGGTGCAGTTCAATATAATGACT
>sRNA0494	s0005599	95	110	28	17	19	14	15	17	NC_004350.2	1E+06	1E+06	-	SMU_1430	6	SMU_1431	144	/+/-/-/	IGR	GATTGCGTTACGACTTACCGATTTTTGTTCCCCCTCTCGTAAATGTTAATAAATTATAATATAAAGCACAGATGTTATTTATCTGTGCTTTTATC
>sRNA0495	s0005604	135	142	21	29	28	27	22	15	NC_004350.2	1E+06	1E+06	+	SMU_1432c	-73	SMU_1434	173	/-/+/-/	AM	TAATATTTGTAGAGCTTAGCAAATTGGGACTTAGTTGTTATGCCCTTCTTAGCAGCTTTAATGGCAATATACATTCCATAACCCTGTCCTTCAGAAAGAGCAGTTGTCCCTTCATCACTAGTAGCCGTCTTTATA
>sRNA0496	s0005621	45	77	16	8	17	14	10	12	NC_004350.2	1E+06	1E+06	+	SMU_1437	152	SMU_1438	131	/-/+/-/	IGR	TTTTCACAAGAGCGTCCAAGGATAAAAATCATTACTAAATAAGTA
>sRNA0497	s0005622	117	603	127	81	134	90	70	101	NC_004350.2	1E+06	1E+06	+	SMU_1437	187	SMU_1438	24	/-/+/-/	IGR	TAAATAAGTATTTGGCAAGCTTTAGAATTTTATCATTTTAGTTTAATGAGATAAAAAATTAATCAAGAGACTGTTTCGTATATGTCAATAATATTAAATTTATATAATAAAGTGATA
>sRNA0498	s0005628	89	485	90	134	95	61	59	46	NC_004350.2	1E+06	1E+06	-	SMU_1438c	62	SMU_1442	451	/-/-/-/	IGR	AATATTATACTGGATTTTCAAGAATGAAATAAATATAAGAATATTTGGATCTTGCTTAGTGTTATAGATTAAAAAGTGAGATGAATATC
>sRNA0499	s0005655	83	308	49	31	72	50	39	67	NC_004350.2	1E+06	1E+06	+	SMU_1444c	169	SMU_1445	-4	/-/+/-/	AM	CTTAGCAAGTCGAATGACTGCGGGCAATAAAAAATTGCATACATTGTTAGTATACAACTTTTCGCCAGTTTTTTCAATTTATT
>sRNA0500	s0005657	117	112	32	24	15	15	6	20	NC_004350.2	1E+06	1E+06	+	SMU_1445c	-61	SMU_1446	-58	/-/+/-/	AM	TAATCATCAAAGCCATTGTTAACTTGAACCGTAGCATTCTTTAATTCAACAATTTTAGTCATCGTGATAACTTCACTCCTTTGAAGAATTTCTTTTTCAGCTCTGGAATCATAAGAC
>sRNA0501	s0005667	138	551	133	39	95	114	70	100	NC_004350.2	1E+06	1E+06	+	SMU_1447c	41	SMU_1449	84	/-/+/+/	IGR	TATGATCAAAATCTAACCTGCTGCTAACAAAAAAAAGCTTTCCCACGCTTACAAGCCAAAAAATTGTCTGTTTAACTCTCTCAAATTCACTAAGTTACTGTTTCTATTTTACTGTAATTTTCAGCTTTTTTCAATATAG
>sRNA0502	s0005676	83	186	35	29	47	25	23	27	NC_004350.2	1E+06	1E+06	+	SMU_1449	92	SMU_1450	131	/+/+/+/	IGR	TAAAGCTTTTCTAGTAAAGCTAACACTCTCTAGTCACTGTTTTCAAGAAATCTAAGTTCTCTTAAGTCCATAAATCTTGGACA
>sRNA0503	s0005697	99	75	12	15	19	12	13	4	NC_004350.2	1E+06	1E+06	+	SMU_1454c	38	SMU_1455	-67	/-/+/-/	AM	AATACGCTTTATCTCTATCAATCAAGCAACTCTATTTTTCATAAAAAAATCACATGTTTATCAACTAGCTTTTGCTCTTGATAAACAAATTTGGCAGAGA
>sRNA0504	s0005710	147	281	40	52	47	60	27	55	NC_004350.2	1E+06	1E+06	-	SMU_1457	124	SMU_1459	134	/-/-/-/	IGR	AAAAAAGTACTGAAAAAAGTACTAGAGAAATAAGGCGAGTTTACGTCATCAAAATTGGCGAGCGATAGCGAGTCAGAGCTATGTGTCGTCATTCTGATGAAAAGAAACATAAATTAGAAAGGAGTGTTCAGTTTATCTTTCTAAATT
>sRNA0505	s0005711	106	309	65	40	50	53	44	57	NC_004350.2	1E+06	1E+06	+	SMU_1457	169	SMU_1459	130	/-/+/-/	IGR	TTTCATCAGAATGACGACACATAGCTCTGACTCGCTATCGCTCGCCAATTTTGATGACGTAAACTCGCCTTATTTCTCTAGTACTTTTTTCAGTACTTTTTTCAAG
>sRNA0506	s0005784	130	67	12	6	10	7	13	19	NC_004350.2	1E+06	1E+06	-	SMU_1477	60	SMU_1479	-114	/-/-/+/	AM	TTTCTTCTGGATCAATGATTGTTTTTTTAACAGCAAAGACTGCCCCAGCAACTGTTGCAGCAGTGCCGATAACACCTGTTGCAAGACCTCTAGCAAATGCATGTTTCTTAGCCATAATTAAGTCTCCTTT
>sRNA0507	s0005896	100	308	52	75	59	50	43	29	NC_004350.2	1E+06	1E+06	-	SMU_1517	50	SMU_1519	204	/-/-/+/	IGR	TAAGTAAATAAAAATGGTTCTAACATAAAGTTTACTCTAAGTGGGCTTGATATAAAAATCAGATAAAAATTAATAAAATAGCATGATTTTTAATCTTTCT
>sRNA0508	s0005898	53	129	26	20	33	16	19	15	NC_004350.2	1E+06	1E+06	+	SMU_1517	98	SMU_1519	203	/-/+/+/	IGR	TATCAAGCCCACTTAGAGTAAACTTTATGTTAGAACCATTTTTATTTACTTAA
>sRNA0509	s0005904	112	277	57	46	49	38	55	32	NC_004350.2	1E+06	1E+06	-	SMU_1520	-61	SMU_1521	-32	/+/-/+/	AM	AAAAGGGCCGGAAGTAGCAGTAAATAGTAGCATAATATCTCCTTTACTCATTATTTATCTGGTTTGGCTGATTCAAGATTATGTTTATTATAAATGCTTTCAAGACTTTTAT
>sRNA0510	s0005911	141	417	54	90	126	52	55	40	NC_004350.2	1E+06	1E+06	+	SMU_1523	-76	SMU_1524	-22	/-/+/-/	AM	AATACGCTCAAAGCAGCAAGGAGAGATAAGCATAATTTTTGCTGTTTTCGCTGAGACTTGTTCGTTTTTCTAGTCATAGAAACTCCTCTAAAAATACCATTAAAAAAGTAGGCTAATCCTACTTTCCAGTAAATTTATCAA
>sRNA0511	s0005919	98	440	58	103	90	85	60	44	NC_004350.2	1E+06	1E+06	+	SMU_1525	16	SMU_1526	-47	/-/+/-/	AM	TTATATTTTATTCTTGCATTATACCACAATAAAGACACTATTCGAATAAATTAAATCGCAAAAAAGATATCTTGACTTAACGAAATGACAGACCAGAT
>sRNA0512	s0005926	63	123	23	25	30	17	14	14	NC_004350.2	1E+06	1E+06	-	SMU_1526c	152	SMU_1527	83	/-/-/-/	IGR	AATTTTCGCATCTTAATTTTTAGGCTTAAACCTTTAACTGTTTAGGAATCATTGAAGCTTATC
>sRNA0513	s0005936	132	273	48	63	46	53	52	11	NC_004350.2	1E+06	1E+06	+	SMU_1529	10	SMU_1530	-122	/-/+/-/	AM	CTCCCATTATTAACTAAATTGTGACTGATCTTTGAAAGTCTGAATGGCTTTGTCCAGCACTGCTTCTTCTGGAAGATCTTTCGTTGTGCGAATCGTTTCAAAGATATCAGCGTCGTGTGAGTCAATGTAATC
>sRNA0514	s0005970	29	70	16	18	16	12	4	4	NC_004350.2	1E+06	1E+06	+	SMU_1541	54	SMU_1542	17	/-/+/-/	IGR	TATGGTAATGTACGACTACGGTATTTTTC
>sRNA0515	s0006010	101	302	59	52	61	60	30	40	NC_004350.2	1E+06	1E+06	+	SMU_1554c	108	SMU_1555	-52	/-/+/-/	AM	AAAAAGACAAACCTCAACAAGAAGTTTATCTTCGGTTTGAAATAGCTTTTAATCGTCTCCTTTTTTTTCATCTTTGTTAAGAGTATGATGTCTGGATTGAA
>sRNA0516	s0006024	43	65	16	15	4	9	9	12	NC_004350.2	1E+06	1E+06	-	SMU_1558c	229	SMU_1560	218	/-/-/+/	IGR	TCAAACTATCACGGCTATTTTATATGACAATCTCAAACTTCTG
>sRNA0517	s0006044	63	87	13	18	13	7	15	21	NC_004350.2	1E+06	1E+06	+	SMU_1563	-35	SMU_1564	245	/-/+/-/	AM	TATTCCCTGCATCGTTTCTTGTTCTGTGCTACTCATAACATATACCTCATTTGACCTCACATG
>sRNA0518	s0006075	81	114	30	24	22	20	6	12	NC_004350.2	1E+06	1E+06	+	SMU_1572	26	SMU_1573	38	/-/+/-/	IGR	TTAAACGATAGAATCTTAACCAAGTTATTTTATCATATATTACACAACATTTCAAACTAACTGAATCATTATAAAATAATG

>sRNA0519	s0006081	51	149	42	23	19	27	23	15	NC_004350.2	2E+06	2E+06	+	SMU_1573	67	SMU_1574	667	/-/+/-/	IGR	AAACACAAGAAATCTTTCCCTTTCAAGTGTTAAACTAGTAAGGCGCTGAGG
>sRNA0520	s0006082	115	247	37	44	40	51	22	53	NC_004350.2	2E+06	2E+06	+	SMU_1573	118	SMU_1574	552	/-/+/-/	IGR	CAGACCGCCAATAGCGGCTACCATCAAATGACTAGCAACTTTAGATGCAGTCATTTAGAGCGAGTTACAAAGAACCGACTGAAAATTGAAAACTTTCCGAGCAATCCGCATTTAA
>sRNA0521	s0006084	85	85	6	23	8	18	15	15	NC_004350.2	2E+06	2E+06	-	SMU_1573	284	SMU_1574	416	/-/-/-/	IGR	GTTAATGAGTCCCGAAAAGGCGGCTTTTGTGTTTAAAATACTTTTATTCTTTCTTGGCTTGTTGTGGTAACCTTAAATGCTAATT
>sRNA0522	s0006088	132	3076	429	649	750	310	600	338	NC_004350.2	2E+06	2E+06	-	SMU_1573	568	SMU_1574	85	/-/-/-/	IGR	ATTTTCCCTTCTTAAGTTTCTTTTAAGAATCCTATCTTATACTATAGTCATCCTAGCAATAGGAATCCTAAAACTTTTCTTTTTCATAAATCTCCTAAGAATCTCAGTCCATTCGGACTGGGATTTTTTTGC
>sRNA0523	s0006089	104	255	43	51	59	27	31	44	NC_004350.2	2E+06	2E+06	+	SMU_1573	586	SMU_1574	95	/-/+/-/	IGR	GAATGGACTGAGATTCTTAGGAGATTTATGAAAAAGAAAAGTTTTAGGATTCCTATTGCTAGGATGACTATAGTATAAGATAGGATTCTTAAAAGAAACTTAAG
>sRNA0524	s0006095	129	267	56	28	49	46	43	45	NC_004350.2	2E+06	2E+06	+	SMU_1574	36	SMU_1575	166	/-/+/-/	IGR	CCTGTGCCTGCATATCATAAGGAGACAAGGTTTTGTCAAAATCATAACAAAGCGCAATAACTGGCTTATCTTCTCTGACTTCACGTTGAAATTCTGTATCGGTCATACTAACACCCCATATCAATCATT
>sRNA0525	s0006098	129	183	29	40	40	21	28	25	NC_004350.2	2E+06	2E+06	-	SMU_1574	191	SMU_1575	11	/-/-/-/	IGR	TTTAAAAATCACTTCTTATTATAAGAATGAGTACTGTTGAGTCTCAGATATAGATTGGAATTTTTGCTTTTGTAAAAATATAAGAAGCTTGCAAACTGTATATTTTTCCCTCAAATCCTTTGCTCATCA
>sRNA0526	s0006134	73	129	31	23	25	18	11	21	NC_004350.2	2E+06	2E+06	+	SMU_1587	-35	SMU_1588	228	/-/+/-/	AM	ACTACAAGGCGATCCGCCAATCATATAAATCACCATTCTCCCTCTTTCTGACAAAAGAAAAACGACATCCTTA
>sRNA0527	s0006136	33	117	23	22	25	19	12	16	NC_004350.2	2E+06	2E+06	+	SMU_1587	225	SMU_1588	8	/-/+/-/	IGR	AAAAATTATTATAGCAGGATTTGGGAATTTTTC
>sRNA0528	s0006162	112	111	21	13	29	21	4	23	NC_004350.2	2E+06	2E+06	+	SMU_1593	-93	SMU_1595	124	/-/+/-/	AM	TAGGCTGATAAGTCCAAGCTAAAATATCAGATTTGCAGAAAAAACGATTGGCAACCCCAGCCAAAAAAACAGGCATGACAGTCACATACATCATCACAATAGTCTTCAAAGG
>sRNA0529	s0006166	65	864	151	134	115	187	133	144	NC_004350.2	2E+06	2E+06	+	SMU_1595	10	SMU_1596	192	/-/+/-/	IGR	TTTTCTATTTTCCAATGACTGACCTTCTTATCCTAAGAGAAAGTCAGCTAGCTTTTAGGTTGACT
>sRNA0530	s0006211	70	81	9	26	19	2	12	13	NC_004350.2	2E+06	2E+06	+	SMU_1611	78	SMU_1612	-25	/-/+/-/	AM	TTAAGAAGCTTGACGTCCTAATTTTCTGGTTTATATAGTCTGACCTAAGCTTCTGGCTTATGGGCTGAAA
>sRNA0531	s0006245	121	366	87	65	55	70	43	46	NC_004350.2	2E+06	2E+06	-	SMU_1623	101	SMU_1624	24	/-/-/-/	IGR	CAGTCGCATTAGGATAAGATATTTATAGATAGATCTTGTCGTCTCATTAAAATAAAGGGCAGAAGAGAAGGGTGTGGCAGATCCACTCCCTTTTGTAAATGTAAAGGAAAGGAATTAAACA
>sRNA0532	s0006269	98	70	7	6	17	21	12	7	NC_004350.2	2E+06	2E+06	+	SMU_1629	-45	SMU_1631	132	/-/+/+/	AM	AAATTGCAAAAAAGAGGACAAAAGCCATAAACAAAGCAAATACCATGCGCTTTATGGCTCTTTGTTTCTCTAATTCCGCCTTTGTTGGACGGCGTGTT
>sRNA0533	s0006288	77	75	17	10	19	10	9	10	NC_004350.2	2E+06	2E+06	+	SMU_1636	27	SMU_1637	-1	/-/+/-/	AM	ATTATTCAGGGTAAGGCAAACAGATAACAGTAGACTGTAGAGATTCTTTAACCACGCAAACATGAAAATGACTGTTC
>sRNA0534	s0006291	133	681	98	123	159	107	95	99	NC_004350.2	2E+06	2E+06	+	SMU_1637	-11	SMU_1638	23	/-/+/-/	AM	CAAAGCATCCATAAGGCAAGGATCTGTAACAATTTATGATGATAAAAATGAATCAAAATAGGGATAAGAACATCAATTTCAAAAAACAAGATGAGGAGCCATAAGGCAAAAGTATCCCCAAGCTTTCCTTTTC
>sRNA0535	s0006298	57	439	67	123	72	57	57	63	NC_004350.2	2E+06	2E+06	+	SMU_1638	-42	SMU_1639	21	/-/+/-/	AM	AATTCTTTTTAATATCTCTATTTAAACTTCCGACTAATAGCATCTGTTATTATTACA
>sRNA0536	s0006317	55	104	16	17	22	20	13	16	NC_004350.2	2E+06	2E+06	+	SMU_1639	538	SMU_1641	-14	/-/+/-/	AM	ACTACTAATTAGATAGGATTTCTGCTCTTTTATATTTAGATTATTCTTTGTTAGA
>sRNA0537	s0006329	73	86	27	17	13	8	14	7	NC_004350.2	2E+06	2E+06	+	SMU_1643	282	SMU_1644	21	/-/+/-/	IGR	CAACTTTTTGAACTGTTTGACCATTGATCCCTAGTCTTTCTTTATCAATTAAAGTTTAAAACGGCAATATCAT
>sRNA0538	s0006341	132	308	68	39	34	85	49	33	NC_004350.2	2E+06	2E+06	+	SMU_1647	-94	SMU_1648	82	/-/+/-/	AM	ATTTCACCAGCTGATAATTGCCCCAACTTTAGTAAATCTAGAATATCGCGTCGAATTGGATTTGACAAAGCCTTAAACGTTTCAGCAAATCCCATGATGACTCCTTCAGTATTTAGAATTATTTCTAAATAG
>sRNA0539	s0006351	139	2468	389	572	466	406	356	279	NC_004350.2	2E+06	2E+06	-	SMU_1648	104	SMU_1649	-9	/-/-/+/	AM	TAAGTTTCATTATTAAGATATTAAGTCCATCCAGTAAGTGAGCAGCATTTGGAAAAGCAAGGTTATGTGCCTGCACACCAACTGCTCTATCTAAATACTCAGACTGTAGGACGGCTTTCTTCCTTTCTAGTCTTGCCTT
>sRNA0540	s0006354	92	219	26	44	54	34	37	24	NC_004350.2	2E+06	2E+06	+	SMU_1649	28	SMU_1650	-54	/+/+/-/	AM	ACTCACTGCAGAGGTTATTTTTATTGGTACCGAATGCTCAATCGAATTCATACAATTGCGGATACTTTTGACATTCAGGATTTTTTGGATGG
>sRNA0541	s0006356	117	211	32	51	50	40	22	16	NC_004350.2	2E+06	2E+06	+	SMU_1650	-16	SMU_1651	-35	/-/+/-/	AM	TTTTTTGATAAAACCATAATTGTCAAATTCTCCTTGGACTCATTATAGCATGAAAAAGAAAGGAAAGAAAAAATCTCGACTTTAAAAGTCAAGATCTTCGTAGGCTGTTCGATAGCC
>sRNA0542	s0006362	103	91	19	9	22	19	12	10	NC_004350.2	2E+06	2E+06	+	SMU_1651	-20	SMU_1652	-45	/-/+/-/	AM	ATATTCGTAAAATGTCAGCATATGTTCTCCTTTCAAATTTATCTTAGCAAAGATAGATTACTTGTCAACTTTAAAGCCTTCATGCTCTAATAACCACATCTTT
>sRNA0543	s0006375	95	171	27	51	29	28	26	10	NC_004350.2	2E+06	2E+06	+	SMU_1656	127	SMU_1657	-1	/-/+/-/	AM	AAAAACGAAAAGCTTAACTTATCTTTAGGACGTACTTATCAAAAAGCTTAAATCTCTCTTTGAGCAGCAATATTAATAGCAAAGACAGGCAACTT
>sRNA0544	s0006382	93	2040	304	559	416	298	239	224	NC_004350.2	2E+06	2E+06	+	SMU_1658	-89	SMU_1659	302	/-/+/-/	AM	CAAGCCTCCATAGAAAAAGGCTAAACCCGGTGTCATTAAAAAAACAAGCGCAGAACAAATGATAATAAATGCTATAGATCCTGAATCCATATC
>sRNA0545	s0006388	141	95	11	25	19	13	7	20	NC_004350.2	2E+06	2E+06	+	SMU_1658	219	SMU_1659	-54	/-/+/-/	AM	ACTCTTATCAATCGATAGTTTATGAATTACGAAGATCATAGAGCGAGACGAACTAATACTCAAAGAAAAAAGACAGTCGAAACTGTCTACTGTAATTCATGATAGAGTTGATATAGTTCTTGACGATTGAAATCATTCTCT
>sRNA0546	s0006482	123	256	54	35	72	48	19	28	NC_004350.2	2E+06	2E+06	+	SMU_1691	-52	SMU_1692	748	/-/+/-/	AM	TGAGCAAAATTTTCAATTGTTGCAATCATATCTTTTATTTTTTTATTAGCCATTGTTACTTCCTAAAATTCGTTGTAGATAAATTCTCCCTGACCTTGACCAAGGTAACTAAAGAAATAGAGT
>sRNA0547	s0006492	122	245	36	37	56	37	41	38	NC_004350.2	2E+06	2E+06	+	SMU_1691	486	SMU_1692	211	/-/+/-/	IGR	CGAGCAACCGACTCCGTCATTTTATTGCTAAAATCGAAACTTGTAATCTCTAATTTGCCAGTATTGTTACCAAAGGTATTGATACTGTGACACAGCAGCAACTGGTCAGTATTCCCATCGCT
>sRNA0548	s0006496	74	103	14	17	21	15	8	28	NC_004350.2	2E+06	2E+06	+	SMU_1691	711	SMU_1692	34	/-/+/-/	IGR	AAACAAGACTATTATTTAATTGGATAAGCTGACTGACTTATTAACTTCACCAAACTTGAAAAAGTAGCATAACA
>sRNA0549	s0006523	67	84	12	16	18	15	15	8	NC_004350.2	2E+06	2E+06	+	SMU_1701	161	SMU_1702	-40	/-/+/-/	AM	AATTATTGAATTGGTATGACTCCTTTTTACTTCTGCTTGCCTGAAAAGCGCCATTTAAAACGCCACT
>sRNA0550	s0006529	56	188	18	24	39	51	25	31	NC_004350.2	2E+06	2E+06	-	SMU_1703	442	SMU_1704	-38	/-/-/+/	AM	TAAGTATTCTATTAGCAGGGCTGAAACAGGATAATACATTCAGTTACCTCCTATAT
>sRNA0551	s0006531	143	233	61	42	42	40	24	24	NC_004350.2	2E+06	2E+06	-	SMU_1704	-69	SMU_1705	-86	/+/-/+/	AM	AAAGTAATCCATTGCTTCTTCATAATCAGTATGTGGTAATCTCTTTAAATAATGTTTTAACTGATTGAGGTATTCAGTTCTTGTCATGGCGAATTCTCCCTTCTACAATGCCATCAATGGTCATTTTATAAACTGACCATTCC
>sRNA0552	s0006533	147	582	103	100	148	95	60	76	NC_004350.2	2E+06	2E+06	-	SMU_1705	-87	SMU_1706	-62	/+/-/+/	AM	AATAAGACCAAAGCAACTTAAGATTAGTCCGATACCTATAGTCATTTTCAGTGATTTTCTCATACTCTGTACCCCCTTTTATAGCCTTGTTTAACAATCCATTGACTCCATTTAATAAAAGCAAATTTAAACCATTGCGCTAATAAA
>sRNA0553	s0006538	127	857	146	146	151	183	101	130	NC_004350.2	2E+06	2E+06	+	SMU_1707	19	SMU_1708	321	/-/+/+/	IGR	ACTTTACAAAAAAAGAACCACGGAACCCGGAGTCCAGCTCAGCAGGTAACGTGGTGTGCGGTGCTTTCATTATCTTAAATCATAACAGGTTTGATTTAAAACAATGATTAGCTTATTACTTAGTATA
>sRNA0554	s0006548	76	73	12	4	14	9	17	17	NC_004350.2	2E+06	2E+06	-	SMU_1709	-53	SMU_1710	45	/+/-/+/	AM	TATGTTAATTGATAAAAAGGACTTAAGTTTTAGACCAAGTTTTTGGAATAAATAGCAACAACATGGGGTAAATTTC
>sRNA0555	s0006589	108	83	19	13	14	16	10	11	NC_004350.2	2E+06	2E+06	+	SMU_1722	-2	SMU_1723	-85	/-/+/-/	AM	CATTTTATTCTCCTAAATTTTTCTAATGATTGTGACTGCCTAAAAACTTACGACGAAAGATACGGCGTTTTTGCCATTTCATTCTTAAAGGGGTTATCGCCTCTTTTA
>sRNA0556	s0006604	105	115	29	20	27	14	10	15	NC_004350.2	2E+06	2E+06	+	SMU_1727	312	SMU_1728	-1	/+/+/-/	AM	TAGAATGGAGAGAAACTAGATGAAGTCAGAAATATTCTTCCCTTTCCTATCTCTCTTTAATAATCAAAATGTTAGAAGCTATGGTTCTAATAAGTTCTTCTTATT
>sRNA0557	s0006628	68	97	13	31	25	5	10	13	NC_004350.2	2E+06	2E+06	+	SMU_1736	6	SMU_1737	94	/-/+/-/	IGR	AAGATACCAAAGGCTAAAAAACAAAACGGAAATAGAAAATCTGCAGAAATCTCTCTAATGATACAAGC
>sRNA0558	s0006664	144	112	20	15	27	14	22	14	NC_004350.2	2E+06	2E+06	-	SMU_1748	-118	SMU_t41	359	/+/-/-/	AM	AATATTGGCCTATTAAAATAACTATTTACTCATCTTTCATAAAAAAATGTTTGGTATAAGGCTCTAACTGCTTGTTTTTCTTGTTCAGTTTTGATAACAAACATGACGGATACTTCACTGGAACCTTGGGAAATCATTTCCAAGT
>sRNA0559	s0006676	109	1346	286	165	257	246	191	201	NC_004350.2	2E+06	2E+06	+	SMU_1748	309	SMU_t41	-33	/+/+/-/	AM	AATGTCAGTATAAGTCAACCCACTATGTTGACAATAAGTCTATATCTCTTCACTACAATTAATAAAGGACTATAATCGGGAAGACAGGATTCGAACCTGCGACCCCTTG
>sRNA0560	s0000078	52	169	23	42	39	28	10	27	NC_004350.2	22705	22756	-	SMU_t09	-19	SMU_t10	22	/+/-/+/	AM	AAAGTGTGCTAGTCCTTTACTAACCCCACTCATGCACCCTGCAGGATTCGAA
>sRNA0561	s0000077	50	82	18	12	10	19	11	12	NC_004350.2	22610	22659	-	SMU_t08	-27	SMU_t09	-8	/+/-/+/	AM	CAAGGGTGCTCCTTCTTATTTATGCCGAGGACCGGAATCGAACCGGTACG
>sRNA0562	s0000076	43	73	18	13	11	14	7	10	NC_004350.2	22527	22569	-	SMU_t07	-17	SMU_t08	-17	/+/-/+/	AM	CAGTTCCGCCACCCCGGCCTCCTAAAGCGAACGACGGGATTCG
>sRNA0563	s0000075	59	1581	278	302	218	350	183	250	NC_004350.2	22447	22505	-	SMU_t06	-23	SMU_t07	-32	/+/-/+/	AM	CAAGGTGATGTTCTACCACTGAACTACGTTCGCAATGCCGGCTACATGACTTGAACACG
>sRNA0564	s0000072	82	825	138	126	115	151	113	182	NC_004350.2	22176	22257	-	SMU_t03	-16	SMU_t04	-37	/+/-/+/	AM	TTAAAAGTCAAATGCTCTACCAACTGAGCTAACGAGTCGATGATCCCTCTCTTTTCAGAAAAAGACGGTCCCGACGGGAATC
>sRNA0565	s0000071	79	436	92	51	76	72	52	93	NC_004350.2	22051	22129	-	SMU_t02	-39	SMU_t03	-9	/+/-/+/	AM	CCACGGGACCTCTAACTAATCACTTTAGTTACTTAGTTATGGGAGTTAACGGGCTCGAACCGCTGACCCTCTGCTTGTA
>sRNA0566	s0000060	118	166	31	34	41	27	13	20	NC_004350.2	18559	18676	-	SMU_t01	1	SMU_r02	141	/+/-/+/	IGR	TAGACTTCCTTGTCTTAAACAAGATATGAAGTTGAACTCCAGACTGACTTCTTAGAAAAATAGATAATCTTCTACAGAAGTATTCGCAAGCGAACCGTCTGTTAGTATCCTGTTTTAA
>sRNA0567	s0000057	48	105	22	14	26	21	2	20	NC_004350.2	16873	16920	-	SMU_18	112	SMU_r01	-43	/+/-/+/	AM	TTAGGCACGCCGCCAGCGTTCGTCCTGAGCCAGGATCAAACTCTCATT
>sRNA0568	s0006681	49	78	11	15	12	18	12	10	NC_004350.2	2E+06	2E+06	+	SMU_r09	283	SMU_1750	481	/+/-/-/	IGR	CTTACATAAAACCCAAGGACGGACTGAGATTAATACAACTATGAAATCA
>sRNA0569	s0006723	98	82	23	29	13	6	2	9	NC_004350.2	2E+06	2E+06	+	SMU_1765	-59	SMU_1766	258	/-/+/-/	AM	ATTTCCTTTTTCCAATTCTGACATAAAATAGAGCAACCGTTTTGTCTTTGACTCCGCCATATTACTTAACCTCGCTGTTCTCTTTTAGTTATATGATA
>sRNA0570	s0006727	150	630	101	94	149	95	88	103	NC_004350.2	2E+06	2E+06	+	SMU_1765	179	SMU_1766	-32	/-/+/-/	AM	AGCCCAACCATGCGGTTAGACTTGTTGAGAAGATTTACTTTCAATATTTCTCTTAAAATTACCAATATCTGAACACTTAGTGACATGGAAATCTACTCACATTTAATATAGCAGCAATTACCTTTTCAAGCGCTCCAAAAATTCCTCGGG
>sRNA0571	s0006739	107	2277	373	440	424	374	307	359	NC_004350.2	2E+06	2E+06	+	SMU_1768	400	SMU_1770	65	/-/+/-/	IGR	CAATGCATTAATGATGCTTTTGGTCAGTTTTTCCCTATCCCTTGATTCTTAGGATGTGTTCTAAGATATGCATGTACAGCTCTTCCAGTATCAGAGCGCGTATAGC
>sRNA0572	s0006753	123	140	12	50	16	28	25	9	NC_004350.2	2E+06	2E+06	-	SMU_1774	38	SMU_t51	-8	/-/-/+/	AM	TATAGCGGCGACTCATCTATCATACTATAAAATAATGAAGAAAGCAAGGAGGGAAACTTAATATCATTTGTTCATTAATTTAAATTAGAAAACGAAATGGCTATCTCTTTTTCTCTTAAAATG
>sRNA0573	s0006755	92	863	164	106	171	218	95	109	NC_004350.2	2E+06	2E+06	-	SMU_t51	-38	SMU_1775	218	/+/-/-/	AM	TAAAAAGGCTCTAAAAAAGCCGACCTAATAGGAATTAGGTCGGTCAAGTTTCATGCCACCACCGAGAATCGAACTCGGAACGGAGCATTACC
>sRNA0574	s0000075	59	1581	278	302	218	350	183	250	NC_004350.2	22447	22505	-	SMU_t06	-23	SMU_t07	-32	/+/-/+/	AM	CAAGGTGATGTTCTACCACTGAACTACGTTCGCAATGCCGGCTACATGACTTGAACACG
>sRNA0575	s0000072	82	825	138	126	115	151	113	182	NC_004350.2	22176	22257	-	SMU_t03	-16	SMU_t04	-37	/+/-/+/	AM	TTAAAAGTCAAATGCTCTACCAACTGAGCTAACGAGTCGATGATCCCTCTCTTTTCAGAAAAAGACGGTCCCGACGGGAATC
>sRNA0576	s0000071	79	436	92	51	76	72	52	93	NC_004350.2	22051	22129	-	SMU_t02	-39	SMU_t03	-9	/+/-/+/	AM	CCACGGGACCTCTAACTAATCACTTTAGTTACTTAGTTATGGGAGTTAACGGGCTCGAACCGCTGACCCTCTGCTTGTA
>sRNA0577	s0000060	118	166	31	34	41	27	13	20	NC_004350.2	18559	18676	-	SMU_t01	1	SMU_r02	141	/+/-/+/	IGR	TAGACTTCCTTGTCTTAAACAAGATATGAAGTTGAACTCCAGACTGACTTCTTAGAAAAATAGATAATCTTCTACAGAAGTATTCGCAAGCGAACCGTCTGTTAGTATCCTGTTTTAA
>sRNA0578	s0000057	48	105	22	14	26	21	2	20	NC_004350.2	16873	16920	-	SMU_18	112	SMU_r01	-43	/+/-/+/	AM	TTAGGCACGCCGCCAGCGTTCGTCCTGAGCCAGGATCAAACTCTCATT
>sRNA0579	s0000054	159	125	19	16	26	31	23	10	NC_004350.2	16693	16851	-	SMU_18	-68	SMU_r01	26	/+/-/+/	AM	ATTACTGTCCACTGACAGATTTATGGTTCGTTCTTTGACAGGTCACATTTCTTTATGTCACCCTGCACTTTGGTTGGTTCGTCTTCTTGTTCAGTTTTCAAAGGGCTCTGCCTCTTTCTGAGACAACTTCTTTATTCTACCAGCTCTTCCCCCCTTTC
>sRNA0580	s0006791	73	96	23	10	25	27	2	9	NC_004350.2	2E+06	2E+06	-	SMU_1784	-27	SMU_1785	-29	/+/-/+/	AM	TAATAATAAAAGCTATTAGTCCTGACATTCTTTCCTCCTCCTTTTAGAACAAACCAAAGAAGTGCATGATTGG
>sRNA0581	s0006800	104	67	8	18	17	13	5	6	NC_004350.2	2E+06	2E+06	+	SMU_1786	52	SMU_1787	-43	/-/+/-/	AM	TTATGAACGAATTTTATTCCAGTTGAAAAAAGCCCCAATCACAACGATTGAGGTTTATAATTATTCTTCAATGGCAGATTCAGTACTTGCTGTTTCCTTAGAAT
>sRNA0582	s0006806	58	106	12	15	23	12	21	23	NC_004350.2	2E+06	2E+06	+	SMU_1787	108	SMU_1788	10	/-/+/-/	IGR	AAAATTCAAAAATTTAAAAGGACGATAACATTAAGAGACCAAAATAGCCAAACAGTCT
>sRNA0583	s0006827	47	107	19	18	19	27	14	10	NC_004350.2	2E+06	2E+06	+	SMU_1791	67	SMU_1792	35	/-/+/-/	IGR	CAATACAAGCGCAGATAGAAAGATGAGACATGTTATTCCAGACTAGA

>sRNA0584	s0006829	27	91	14	14	21	17	8	17	NC_004350.2	2E+06	2E+06	+	SMU_1791	109	SMU_1792	13	/-/+/-/	IGR	CTAGATAGGCTGTCTGTTTCGTAGAAA
>sRNA0585	s0006834	77	106	20	12	21	18	15	20	NC_004350.2	2E+06	2E+06	+	SMU_1792	-18	SMU_1794	15	/-/+/-/	AM	CAATGGCTGTATAAATCATGAAAATTGCTGTTGCATAGATGCCCTTTCGGCTTTTTGGGCTATTTTGACTGTTTGTT
>sRNA0586	s0006870	79	156	12	35	24	42	25	18	NC_004350.2	2E+06	2E+06	-	SMU_1804	124	SMU_1805	36	/-/-/+/	IGR	TTTTTGCTTCTTGTTTCAACGTGTAGTCCCTTCTTTTTCATAAAACAGACCAACAGTCTGTTTTTTAGATTATAGTAAC
>sRNA0587	s0006873	104	70	21	11	12	10	4	12	NC_004350.2	2E+06	2E+06	-	SMU_1804	187	SMU_1805	-52	/-/-/+/	AM	ACAATTTCCCGCATTGTTGTCTCTTCATAACCCTTTTGCAAGAATAATGTCATCGCTGTTTGGGCAATTCTCTTTTTAGATAATTGCGTTTTTGCTTCTTGTTT
>sRNA0588	s0006951	142	201	45	33	55	22	18	28	NC_004350.2	2E+06	2E+06	-	SMU_1818	110	SMU_1819	2	/-/-/-/	IGR	ATGTAATTATTTTGTTTTTACAAATAAAGCGAGCTGCTTTGAACTGCACCTCAAAAGTTGAACAAGAAATTATGGCAAGGATTGGATTTGATATAATAAAGTGGACACGAAGTTAAGTGAAAACGAGGAAAAATATTATGCC
>sRNA0589	s0006952	66	68	13	6	14	22	4	9	NC_004350.2	2E+06	2E+06	+	SMU_1818	118	SMU_1819	70	/-/+/-/	IGR	ATTTTTCCTCGTTTTCACTTAACTTCGTGTCCACTTTATTATATCAAATCCAATCCTTGCCATAAT
>sRNA0590	s0006964	128	463	83	84	87	69	62	78	NC_004350.2	2E+06	2E+06	+	SMU_1822	-11	SMU_1823	142	/-/+/-/	AM	TAATTCTTTCATGATGTCCTCTTCTTCGTAAAATAAAAAGCACCACCTTCATCTAAACAGACGAAGACCGTGGTGCCACTGTTATTTGTCATCAATAAATTGATAACCTCTTGATTCGTAACGTGAAT
>sRNA0591	s0007012	28	121	11	21	17	27	22	23	NC_004350.2	2E+06	2E+06	+	SMU_1835	99	SMU_1836	144	/-/+/-/	IGR	CATTACGATTTATCGTCCTGCTTTCCGA
>sRNA0592	s0007041	115	502	61	99	111	97	87	47	NC_004350.2	2E+06	2E+06	-	SMU_1841	175	SMU_1843	-93	/-/-/+/	AM	AAGGAGACAAAGCTACATTGGTTTTAATACTTTTTATTTCTTCTTCAGTCCAATCTTGATAGCGGCGATATCTGATATTTTGAGGCAAATTCATCGTTTATCTACTCCTAATAAT
>sRNA0593	s0007054	103	266	37	73	67	24	47	18	NC_004350.2	2E+06	2E+06	+	SMU_1846	-30	SMU_1847	-29	/-/+/-/	AM	TAACAATTTCGCCAATCATTTCATTTTCCATCAAACTTGTCCTTTCTAATAAATTCATCCAAAACTGTTTTCCTTAGGCACGGGAAACGTAGGTTCCCTCAGC
>sRNA0594	s0007072	92	83	12	11	26	20	10	4	NC_004350.2	2E+06	2E+06	+	SMU_1851	250	SMU_1852	330	/-/+/+/	IGR	TAATACAAAGGGCATAAAATAAAGTACAAAGGGAAAAAAGATTAATGATGAGTTGATAACACTCAAGGAAACCTACCTTTTTTCACACAGTG
>sRNA0595	s0007073	146	698	119	130	134	132	105	78	NC_004350.2	2E+06	2E+06	+	SMU_1851	332	SMU_1852	194	/-/+/+/	IGR	TCACACAGTGTTAAACTCGTGTTCTATCCTCTCAAAAGAATACAAGGTTCCTAAAAAGCGACTGAATTTTAGAAAAGCAATGCAATGCTGTATGCTTTGCATACCAAACATTTACTCCTTAAGGCAATCCTCTTTGTAAATCAATG
>sRNA0596	s0007074	54	69	10	9	15	20	8	7	NC_004350.2	2E+06	2E+06	+	SMU_1851	467	SMU_1852	151	/-/+/+/	IGR	GTAAATCAATGTAAGTCGAAAAAAATATGTCTCTAAATTTCGAGCATTTAGAAC
>sRNA0597	s0007075	37	93	7	30	27	20	7	2	NC_004350.2	2E+06	2E+06	+	SMU_1851	502	SMU_1852	133	/-/+/+/	IGR	AATTTCGAGCATTTAGAACATCATCTAAACTTTTCTC
>sRNA0598	s0007088	132	237	64	26	33	44	26	44	NC_004350.2	2E+06	2E+06	-	SMU_1854	-54	SMU_1855	-80	/+/-/+/	AM	AAATCCCGGGATAAAAGCAATAGTAATAAGGTAAAGTGCGAGAAGCCCAAGCAGACCGTACCAAAAATAATTTTTTTTCATAGTAAACTCCTTTTTTTCATAAGTAATTCTTTGTAGTATTTACGAGATACC
>sRNA0599	s0007093	144	577	105	96	139	109	73	55	NC_004350.2	2E+06	2E+06	+	SMU_1856	234	SMU_1858	-58	/-/+/-/	AM	ACTCAGCCCTGTGCAATACAGGACTAAATCCTTTCAATAATTTCTTGTCCAACTTTTTGGGGTCAGTACAATTAGGGTCTTTTTATTAATCTTCATTTACAAAAGGCATAAGTGCCATTACGCGAGCACGTTTGATAGCTGTTG
>sRNA0600	s0007112	139	116	30	8	14	19	9	36	NC_004350.2	2E+06	2E+06	+	SMU_1862	107	SMU_1865	792	/+/+/+/	IGR	TAGCTTTTCAACTTTAGCAAGAATCAGTACAACAACTCCTAGCAAAGCTGTTCGCTGTATTTGTTTCGGACCTTAGTCTCTTAGAATATCGCGTAATAGCGATTTATGCCATTTTTTACTTTAAAATCAAATAGTTGGT
>sRNA0601	s0007120	49	63	5	9	19	17	0	13	NC_004350.2	2E+06	2E+06	+	SMU_1862	649	SMU_1865	340	/+/+/+/	IGR	TATAATAAAATACATTTGTCTCTGGTTTCTTGTTCTCCACATTCCTCAT
>sRNA0602	s0007121	69	72	18	2	13	14	12	13	NC_004350.2	2E+06	2E+06	+	SMU_1862	743	SMU_1865	226	/+/+/+/	IGR	TTATAAAGTTACTGCCTGCTGTCTATTCTTTGTCACCATTTTTATAGCGACCTACATCAATCATTAACA
>sRNA0603	s0007149	68	250	39	52	38	43	34	44	NC_004350.2	2E+06	2E+06	-	SMU_1874	-11	SMU_1875	30	/+/-/+/	AM	AATAAGTTCAAAAGATTTCTGAACTGTACTCTGCTTCCGTTAGAGATAAAAACTTTTTATTTATTTGA
>sRNA0604	s0007156	74	152	23	25	31	21	25	27	NC_004350.2	2E+06	2E+06	-	SMU_1875	88	SMU_1876	-62	/+/-/+/	AM	AAGAACAGCGATAGCAATAGTGCTGCTTTTAAAAGCTCGGGCTGTTTTTTCGTATGATATCATGTAAACCTCCA
>sRNA0605	s0007158	131	1296	192	173	295	234	236	166	NC_004350.2	2E+06	2E+06	-	SMU_1876	-68	SMU_1877	815	/+/-/+/	AM	TTAGCATTAAAATGTATGAAACTGCCAGCTCTAAATAAGAAAATGAGCGTTTTACGTCAACTTTAAGCCTCTTCTTTTTCTGCTAGTATCCTTGCTTTTTGGATAGTATAGAAATAAAGAACAATAAAAGC
>sRNA0606	s0007160	133	770	156	125	119	141	103	126	NC_004350.2	2E+06	2E+06	+	SMU_1876	16	SMU_1877	729	/+/+/+/	IGR	CTCATTTTCTTATTTAGAGCTGGCAGTTTCATACATTTTAATGCTAAAAAAGAGTGTGATAAGAAGTAAAAATCTTAGATTTTCTGACTTCTATATTACGCTCTTTTTTGCCTATCTCAAGCAAGAACACTTC
>sRNA0607	s0007168	34	66	10	11	12	9	14	10	NC_004350.2	2E+06	2E+06	-	SMU_1876	410	SMU_1877	434	/+/-/+/	IGR	TTTAATGCAGTGGATGATTGGAGATTACCTAATC
>sRNA0608	s0007175	111	88	15	19	15	11	17	11	NC_004350.2	2E+06	2E+06	-	SMU_1876	841	SMU_1877	-74	/+/-/+/	AM	AAAGATCATAGAACCAGATTGATGAATGCCGGCGGCAAATTCGCCATGACTGGCGATAACGATTCCGATAGCCATTCTTGTTCTTCCTCCTTTTAAATATTTTTTCTCAGA
>sRNA0609	s0007182	127	81	8	14	13	13	13	20	NC_004350.2	2E+06	2E+06	-	SMU_1879	-79	SMU_1881	285	/+/-/-/	AM	ATACCCAATTTTTTTGAAATCAGATATGGAAAATTCTAATTTTTTGTTTACATCAATCCAATGACGTGAGCAATAATACCTACAACGAACAGTCCAAGAATCATTACAATTGGAGAAACTTTTTTCT
>sRNA0610	s0007183	130	198	32	40	38	26	23	39	NC_004350.2	2E+06	2E+06	-	SMU_1879	82	SMU_1881	121	/+/-/-/	IGR	CTATCATCTCTCAAGGTGAGCAAGGATAACTTACTTCATAAATGGAGAATTGAATGTTAACTTTTACACAAATGTGAAGTGCAATCCTAATTGTTAGATTTTTTTCTAATTGTTGGGATGCATTTTATTT
>sRNA0611	s0007184	40	127	15	20	46	21	14	11	NC_004350.2	2E+06	2E+06	+	SMU_1879	108	SMU_1881	185	/+/+/-/	IGR	AAAAAATCTAACAATTAGGATTGCACTTCACATTTGTGTA
>sRNA0612	s0007189	33	282	51	55	56	43	41	36	NC_004350.2	2E+06	2E+06	+	SMU_1879	203	SMU_1881	97	/+/+/-/	IGR	AGATGATAGAAAGCTCTTTCTTGATAAAACAGA
>sRNA0613	s0007190	104	1074	155	151	200	229	184	155	NC_004350.2	2E+06	2E+06	+	SMU_1879	264	SMU_1881	-35	/+/+/-/	AM	AAGTACAACTAACACTGTCAGAAAATTATGGCAGCGACGGGGGCTTTCGGATAATTTAAATGTCTCCCTTAGTTAAACAGCTTGTAGTAGAAGCCTTTATGAGC
>sRNA0614	s0007193	76	587	104	140	117	77	79	70	NC_004350.2	2E+06	2E+06	+	SMU_1881	17	SMU_1882	458	/-/+/-/	IGR	TCAAAGGCACTTAGAAGACTCAAAAATTTCTTCTGTTTAACATTTGTAAATGTTGTTAAATCAAGATTCTGATTTA
>sRNA0615	s0007200	149	294	53	67	46	61	45	22	NC_004350.2	2E+06	2E+06	+	SMU_1882	100	SMU_1883	71	/-/+/+/	IGR	ATATAATAGCAAATATTCTGCTATTATAATTATTTTCTTAGTATCTACTCACCTTTATTTTTTTATTCAAGATAAAGAGGAAGTTTTTTATGTCCAAAAGCTGAGCAACAGATCTAGGGAGCCTCAGCAGTATAGCTGATTTCGCTAAT
>sRNA0616	s0007228	113	719	136	84	167	80	143	109	NC_004350.2	2E+06	2E+06	+	SMU_1892	59	SMU_1893	-51	/-/+/-/	AM	TAACCCTTTGTAAAAAAAGAGACAGGACTTTGTACTGACCCCAAAAAGTTAGACAAAAAATTTAAGTAAAGGATTTCGTTCTGTACTGTACAGGACTAAGTCCTTTTAGTTTT
>sRNA0617	s0007231	48	109	15	15	15	18	13	33	NC_004350.2	2E+06	2E+06	+	SMU_1894	15	SMU_1895	47	/-/+/-/	IGR	AAAAGTTAGATTTTTTCTGTCTAACTTTTGGGGTGCAGTTCACTTTTG
>sRNA0618	s0007240	107	85	22	11	11	17	13	11	NC_004350.2	2E+06	2E+06	+	SMU_1896	141	SMU_1897	93	/-/+/+/	IGR	AAATTATAAATTGGAGCTTGCAGTTATATTTTTAATATAACGTAACAGCAATATCGTAAACGGTAGTGATTTCATCTTAAATGGTTATTGACTAAATGATTTAAGAT
>sRNA0619	s0007254	124	207	31	14	75	32	34	21	NC_004350.2	2E+06	2E+06	-	SMU_1900	69	SMU_1902	70	/+/-/+/	IGR	TATGTCATCTTTAATCAATAACTCAATGAAAAATCAAAAAGTAAACCAGAAAGCTAGCTGCAGGCTGCCCTGAGGCAGGGTAAGACGACACTGATGCATTTGATTTTGACTTTCGCAAGGTATG
>sRNA0620	s0007268	54	161	24	23	29	27	28	30	NC_004350.2	2E+06	2E+06	+	SMU_1904	220	SMU_1905	213	/-/+/-/	IGR	CCTAATGATAGCTGCTGAATTAATGATGTATCAATATAAAAAATAAAATGTCGC
>sRNA0621	s0007270	137	71	15	10	20	8	4	14	NC_004350.2	2E+06	2E+06	+	SMU_1904	314	SMU_1905	36	/-/+/-/	IGR	TATAGATATTATCGCTATTTGGAAGAATCCCGAAAAAATAAATACAGAAGATAGAACTCTACGCAAATCAAGGTTAAACTTACAAAAATATCAGTACTGCGAAAAATTCCAACTATCCCTAATATCATAAAAATAGC
>sRNA0622	s0007272	126	91	9	9	22	12	32	7	NC_004350.2	2E+06	2E+06	+	SMU_1904	434	SMU_1905	-73	/-/+/-/	AM	AATATCATAAAAATAGCTAAACCTACTTTAGTTCTCATTACTTTAACTTTTATCAACAAAAACTTCCCGCTGCAATTGTACCGGCTGCCATGGCAACTCCAACAGGATCTCCAACTACTGCTAAGC
>sRNA0623	s0007292	93	116	10	23	32	15	14	22	NC_004350.2	2E+06	2E+06	+	SMU_1908	85	SMU_1909	598	/-/+/-/	IGR	AATTATTCTTCATGGACCACCTACAAGAGGTATATTCTAATGCTATAGCGGTAACAACGACCGCAGGAGAACTACTCTATTAGTAATAATATC
>sRNA0624	s0007293	36	78	14	14	17	6	13	14	NC_004350.2	2E+06	2E+06	+	SMU_1908	224	SMU_1909	516	/-/+/-/	IGR	TTTATTATAGAGCGTAGATTTACTTTTTAATAAGTA
>sRNA0625	s0007303	63	81	11	10	22	2	22	14	NC_004350.2	2E+06	2E+06	+	SMU_1908	727	SMU_1909	-14	/-/+/-/	AM	TAACAATTGTTCCTATTCAGCATTATATTGCCTGAAAGTAAAAACTGTTTAGCGTCTCTTATG
>sRNA0626	s0007309	66	69	16	11	13	6	10	13	NC_004350.2	2E+06	2E+06	+	SMU_1909	64	SMU_1910	18	/-/+/-/	IGR	AAATCATTCAACTCTTGAATAATTGATAATCTGACATGTAATCATTAGAAAAATAATGCGATAAGC
>sRNA0627	s0007312	136	121	22	17	27	18	15	22	NC_004350.2	2E+06	2E+06	+	SMU_1910	-43	SMU_1912	723	/-/+/-/	AM	TCACGAGAATAAGTAATTTTTGCTTTTTGTTTACTTTTTTTCATCATACCTTCTTTATTCTATTTCTTCTTGTACAGGATATACTTGTAGGGGTGTTAGATTGTAGTTAACAAAAAGTCCACTTGGATACATACTG
>sRNA0628	s0007314	129	443	55	65	117	48	54	104	NC_004350.2	2E+06	2E+06	+	SMU_1910	82	SMU_1912	605	/-/+/-/	IGR	GATACATACTGTAAGTGACCAAACAAACGAAGATCGTCCAAGCAAATCGTAATTAGCCTTTGTTATCAAAATACCATCGGTGAGAGGTTTAAAAAGGTATGAATTTTGGTTAGATCAAACCATCTTTTA
>sRNA0629	s0007315	82	68	13	10	13	5	8	19	NC_004350.2	2E+06	2E+06	+	SMU_1910	205	SMU_1912	529	/-/+/-/	IGR	CTTTTATAACTTAGGATTTTTTAACTAGCACCACAGATTTATTTAATGAATTATAAATAAAGGTTCTAATTAATTCATTAAA
>sRNA0630	s0007319	108	159	22	30	33	23	21	30	NC_004350.2	2E+06	2E+06	+	SMU_1910	402	SMU_1912	306	/-/+/-/	IGR	AATTGTTATAAAAACATGTATGATGGAAGAAAAAGCAGATCTTAGACTTACTTTTTAATAATGAACTTATTACTTAAACTTTAGAAATTCGTTTAACCAGTTATTTCT
>sRNA0631	s0007322	66	93	18	9	22	11	9	24	NC_004350.2	2E+06	2E+06	+	SMU_1910	628	SMU_1912	122	/-/+/-/	IGR	ACGTCCAATAGTATAATACAATTATTAATCAATTTTGCTCTGATCATTCACAAGTTCTCCCATAGG
>sRNA0632	s0007329	90	1129	179	205	248	139	202	156	NC_004350.2	2E+06	2E+06	+	SMU_1912	9	SMU_1913	313	/-/+/-/	IGR	TAAATTAACTTTCATAAAAGGAAAAGACTCTTCTGGTTCAGATAAATATGAACGCATTTTTAAAAGTTGCTTAATTCTGTTACAATGATA
>sRNA0633	s0007343	87	60	19	9	10	9	8	5	NC_004350.2	2E+06	2E+06	+	SMU_1916	-23	SMU_1917	-66	/-/+/-/	AM	AAGTATCATTAAGGCTTCATTCATTTTGCTCTCCTTTGATCAGCAATCACAGCTCTCAGTTTTGTTAACTTAGTTCGTGAAATAAGA
>sRNA0634	s0007346	135	389	66	65	88	67	58	45	NC_004350.2	2E+06	2E+06	-	SMU_1917	26	SMU_1918	312	/-/-/-/	IGR	TATCACTAGAATGAACAAAAAATGCTTAATCATCTGATTATATTGCGAATAGATAAGTAGGGTTATTAAGTTAGTAGATTTTATTGAGTGATATCGTTTTATACCTTTTCTGTGTATAGTGTTAATCAGCTAACC
>sRNA0635	s0007348	67	63	5	4	24	7	12	11	NC_004350.2	2E+06	2E+06	+	SMU_1917	67	SMU_1918	339	/-/+/-/	IGR	ATATCACTCAATAAAATCTACTAACTTAATAACCCTACTTATCTATTCGCAATATAATCAGATGATT
>sRNA0636	s0007369	99	76	19	6	19	14	11	7	NC_004350.2	2E+06	2E+06	+	SMU_1921	-42	SMU_1922	-55	/-/+/-/	AM	TATGGTTATCTTTTTTAGCCAATGTCTGTCCAATTGTTTCCACTTACTCACCATCCCTTCTCAATCTTGCTAAAGTTTTGCGTTTAATCTCGTCTAAAC
>sRNA0637	s0007372	121	71	25	4	13	6	7	16	NC_004350.2	2E+06	2E+06	+	SMU_1923	-58	SMU_1924	308	/-/+/-/	AM	TCAGCTTGACGGCTATCAACAACACTGGATTTTAGATAATTACATTTTGGACAACGCATGAAAACACCTCAGTTCTAAGACTCAATTAATACTTTATTTTACCATATTTTTCATCATAACA
>sRNA0638	s0007377	68	141	31	25	22	23	25	15	NC_004350.2	2E+06	2E+06	+	SMU_1923	202	SMU_1924	101	/-/+/-/	IGR	AATGATACCTATGAGGTCCAATTTCTAATCTTATCGCCTGTCATGGTTGTAACTGTTTTACTCAAGCC
>sRNA0639	s0007470	77	938	138	225	178	135	128	134	NC_004350.2	2E+06	2E+06	+	SMU_1947	33	SMU_1948	47	/-/+/-/	IGR	CTAGATGGCTTTTGGAAATTTTCGTACATTATTATATCATAAAAGCTTAAGAACCCCTAGTCTTTTTTATTAAATAA
>sRNA0640	s0007471	135	177	42	24	51	22	20	18	NC_004350.2	2E+06	2E+06	+	SMU_1947	57	SMU_1948	-35	/-/+/-/	AM	TACATTATTATATCATAAAAGCTTAAGAACCCCTAGTCTTTTTTATTAAATAAACAAATTTCTTCATAAAAAACAGCCTTTTCCAGCTGTTTTTTACTTTTAAAAACGTTTTAATAATTCAGCCAATCCCAGAGT
>sRNA0641	s0007475	26	173	37	29	33	26	16	32	NC_004350.2	2E+06	2E+06	+	SMU_1948	140	SMU_1949	50	/-/+/-/	IGR	CTAGACTTGCTTTTTTCTGCGCCATA
>sRNA0642	s0007484	105	75	12	22	15	15	2	9	NC_004350.2	2E+06	2E+06	+	SMU_1951	-36	SMU_1954	576	/-/+/-/	AM	TAAAGTATTTCTGCCACTTTTCTTTTAATCTATCCATCTTGAACTCCTTGAATCAATTAAAATTTTATAATAGACTTTAGAAAATCTATTCTTATCTGATTGACT
>sRNA0643	s0007489	27	72	14	16	10	18	7	7	NC_004350.2	2E+06	2E+06	+	SMU_1951	73	SMU_1954	545	/-/+/-/	IGR	TTTTGTCAGTCAGCTCTCAGTATTTGG
>sRNA0644	s0007492	59	98	14	26	16	14	23	5	NC_004350.2	2E+06	2E+06	+	SMU_1951	153	SMU_1954	463	/-/+/-/	IGR	ATATCGAAAGTCCCCTCAATATTGTCTCTGACCTCAATAACTTTTTACCACGGCGTAAG
>sRNA0645	s0007503	133	494	74	104	124	81	44	67	NC_004350.2	2E+06	2E+06	+	SMU_1951	584	SMU_1954	-72	/-/+/-/	AM	TAAAAATATTAATGACCAGCATTAAGCCGTTTCACGGACTTGCCACGATAAGAAGCTAAATTACATCATACCACCCATCATGCTTGGATCCATAGCGGGAGCTGCTGCTGGTGCTTCTGGAGCTGGATGATCC
>sRNA0646	s0007510	66	136	27	14	32	15	27	21	NC_004350.2	2E+06	2E+06	+	SMU_1955	-30	SMU_1956	87	/-/+/-/	AM	CGACCACTCGGTCTCCTAAGGGTTTTAACATAATATACCTCCATAGATTTTTAGCACTCCAATATA
>sRNA0647	s0007521	111	141	20	15	32	29	23	22	NC_004350.2	2E+06	2E+06	+	SMU_1958	17	SMU_1960	247	/-/+/-/	IGR	TTAAGATACAAAGAGCGTTCACGAGCAAAGGCAAAATAGGAGTTTTGACGAAGAGTCACAGGACTGGACTCTAGAAGAAACTACCTTTTTGACATAGCTCGTAGCTCGTGT
>sRNA0648	s0007523	54	229	43	29	66	18	54	19	NC_004350.2	2E+06	2E+06	+	SMU_1958	135	SMU_1960	186	/-/+/-/	IGR	ATTAAGATACAAAGGCGTAAAGCGACACACCGCAAAATAGGAAAGCTGATGAAG

>sRNA0649	s0007524	101	115	20	14	27	22	12	20	NC_004350.2	2E+06	2E+06	+	SMU_1958	224	SMU_1960	50	/-/+/-/	IGR	ACAGTGTCGTAACTCATGTTCTTTTTTACTTTTTGATACTTTTTTAAGCAGTACGGACGTGAGTGACCCTGTTTGGAATATCATTGCGAAAAGCCTTCGCT
>sRNA0650	s0007570	119	170	24	51	48	18	17	12	NC_004350.2	2E+06	2E+06	+	SMU_1975	35	SMU_1976	-100	/-/+/-/	AM	TAATTAGCATCTTTTACATCACAATAAGTGATTGAAGAACACTCTAAGTAAAACGCCACATATGATTGTCCCATAAAGAAGAATCATCAGAGTAATCAGATAGCTGAAAGCGATATGCC
>sRNA0651	s0007573	57	113	12	11	30	26	15	19	NC_004350.2	2E+06	2E+06	+	SMU_1977	-23	SMU_1978	536	/-/+/-/	AM	TAATTCTTGAATTCTCGTCTGCATCATTGTCCTCCAAGAAAGTTTAATGGGCATTAT
>sRNA0652	s0007575	86	208	47	32	31	33	37	28	NC_004350.2	2E+06	2E+06	+	SMU_1977	88	SMU_1978	396	/-/+/-/	IGR	AATAGACCCATAAAAGAGATAAACTGATACTCGCTCCTGCTAAAAGCTTGTACAAAAATCTAAATAGCACTTGTATAGCTGTTATG
>sRNA0653	s0007576	48	259	42	35	52	50	37	43	NC_004350.2	2E+06	2E+06	-	SMU_1977	138	SMU_1978	384	/-/-/-/	IGR	TTTATTTATTGTCATAACAGCTATACAAGTGCTATTTAGATTTTTGTA
>sRNA0654	s0007577	76	88	19	9	18	14	12	16	NC_004350.2	2E+06	2E+06	+	SMU_1977	161	SMU_1978	333	/-/+/-/	IGR	TATAGCTGTTATGACAATAAATAAACCAATTAATAGCAGTGTTAATTTAACTATTTGACTATTTGACTATTTGACT
>sRNA0655	s0007579	118	655	114	118	105	119	120	79	NC_004350.2	2E+06	2E+06	+	SMU_1977	182	SMU_1978	270	/-/+/-/	IGR	TAAACCAATTAATAGCAGTGTTAATTTAACTATTTGACTATTTGACTATTTGACTATTTGACTAAAGTTGTACTGAAATATTCTTGACTAAATATCTTTTTAATGATCATAGCATATC
>sRNA0656	s0007580	113	221	65	18	22	63	23	30	NC_004350.2	2E+06	2E+06	-	SMU_1977	235	SMU_1978	222	/-/-/-/	IGR	GTAAAAGAGATTTGACATCTCTCACTAAATAGTAATTATGGGGGATAAGATATGCTATGATCATTAAAAAGATATTTAGTCAAGAATATTTCAGTACAACTTTAGTCAAATAG
>sRNA0657	s0007602	51	163	37	32	29	21	27	17	NC_004350.2	2E+06	2E+06	+	SMU_1987	80	SMU_1988	0	/-/+/-/	IGR	TGAGAACGAATCTCAGTCTAAAACGATATACAAATACTGAATTAACTTTTC
>sRNA0658	s0007607	116	264	60	38	33	56	35	42	NC_004350.2	2E+06	2E+06	+	SMU_1988	94	SMU_1989	-81	/-/+/-/	AM	TATCTTTTTAAGAGATAAGTATACTTTTAAATAGTTACTCTTCTACTGTGTCCTCTTGTTCTTCAGAATCTTCAATGACTTCAATTTCATTGACAGCCAATGGTTCAAGATTACGG
>sRNA0659	s0007615	138	135	19	24	36	24	21	11	NC_004350.2	2E+06	2E+06	+	SMU_1990	-77	SMU_1991	201	/-/+/-/	AM	TAAATCAAGAACCTCTTTGATTCTTGAAAAACTACGACGCGTACGATGTTTCCCGTACTGAACTTCATGTCCTGCCAAGTTTTAGCTCCTTCTTCTTTCTAGATCTGCCAAGCAGATCGGTACGTTAACAATTTGTCT
>sRNA0660	s0007620	108	258	52	31	60	38	39	38	NC_004350.2	2E+06	2E+06	-	SMU_1991	127	SMU_1992	-78	/-/-/+/	AM	TAAGGGCTTCTTCATCAGTGGTTTGAAAGACTAAGCCACGCGCTTTGAGTTCTTCAAAAATAGTCATAGAGTTCTCCATATTCTTATAACATTACAGGACATTATATC
>sRNA0661	s0007628	106	582	126	68	89	99	106	94	NC_004350.2	2E+06	2E+06	+	SMU_1994	-54	SMU_1995	-48	/-/+/-/	AM	AAACAGGTTCTGCATCATACTGATAAGACAGATTTTCAACAGTAATGTATCTCACAATTATTCCTCTTCTAATTCCTTAGTTAAAGCCGTTAAAAAGCGACTAATC
>sRNA0662	s0007633	33	113	19	18	15	17	12	32	NC_004350.2	2E+06	2E+06	+	SMU_1995	246	SMU_1996	357	/-/+/-/	IGR	AAATACAGTGTTTGAAGGGTAAGAGATATAAGC
>sRNA0663	s0007635	137	915	199	143	164	154	135	120	NC_004350.2	2E+06	2E+06	+	SMU_1995	422	SMU_1996	77	/-/+/-/	IGR	AAAATTAACCACATTCTAAGTAACTTTGTGGAATGAACTGCCTACTTCAAACAGTGAATAATTTTTTACCCTTACCTTAAAAACAGGGGAGTGAAAAAATTCATTTTGAAAAAACCGCAAATCATGACGTTCATTCG
>sRNA0664	s0007660	27	107	21	9	22	15	16	24	NC_004350.2	2E+06	2E+06	+	SMU_1997	605	SMU_t59	-12	/-/+/-/	AM	TATGAATATATAAACTCCGGCAGTAGG
>sRNA0665	s0000060	118	166	31	34	41	27	13	20	NC_004350.2	18559	18676	-	SMU_t01	1	SMU_r02	141	/+/-/+/	IGR	TAGACTTCCTTGTCTTAAACAAGATATGAAGTTGAACTCCAGACTGACTTCTTAGAAAAATAGATAATCTTCTACAGAAGTATTCGCAAGCGAACCGTCTGTTAGTATCCTGTTTTAA
>sRNA0666	s0000057	48	105	22	14	26	21	2	20	NC_004350.2	16873	16920	-	SMU_18	112	SMU_r01	-43	/+/-/+/	AM	TTAGGCACGCCGCCAGCGTTCGTCCTGAGCCAGGATCAAACTCTCATT
>sRNA0667	s0007665	34	66	12	9	15	12	9	9	NC_004350.2	2E+06	2E+06	+	SMU_1999	154	SMU_2000	39	/-/+/-/	IGR	TAGTGGTAGACCAGAGCTAGACTAAGAATTATTA
>sRNA0668	s0007672	30	147	25	37	27	18	28	12	NC_004350.2	2E+06	2E+06	+	SMU_2001	17	SMU_2002	0	/-/+/-/	IGR	AAACAATCCTGTGTACTATTAACTTATGAT
>sRNA0669	s0007713	82	1404	199	343	246	227	199	190	NC_004350.2	2E+06	2E+06	+	SMU_2017	-53	SMU_2018	-1	/-/+/-/	AM	TTCGCGGGCACCACTGTTGTCAGCGACTTTTAAACGACTTTCTGATTGAATCATTTAAGTTTTTCTCCTTTCAGGCTTAATT
>sRNA0670	s0007768	94	661	111	80	163	119	90	98	NC_004350.2	2E+06	2E+06	+	SMU_2033	-29	SMU_t61	215	/-/+/+/	AM	TAAAAAACGTAACCGAAAATCTTTTATCATTTTTCTCCATTAGATCGCTGTAACATTCTCTTCATAACTTTATATTTTTTATGCAGATGATTGC
>sRNA0671	s0007779	142	83	11	18	10	19	11	14	NC_004350.2	2E+06	2E+06	-	SMU_t61	398	SMU_2035	-94	/+/-/+/	AM	TTAGCCGATAAATTAGCATTGAATCCACCTAAACTTTCAATAGAATTTGATGGACTAAGCACTCTGATATGATCACCATATGTTAGTTTTTTCATTCATCTTTCCTCCCCATAAGTAATCCCCTAAATGTGACATTTTTTCT
>sRNA0672	s0007782	41	73	10	13	22	10	8	10	NC_004350.2	2E+06	2E+06	+	SMU_2035	66	SMU_2036	39	/+/+/-/	IGR	TAAAATTTTCAAGCGTTTTATTTTAGATTGTTTAATATAAT
>sRNA0673	s0007784	131	73	14	8	23	11	8	9	NC_004350.2	2E+06	2E+06	+	SMU_2035	107	SMU_2036	-92	/+/+/-/	AM	AAAGCGAGTTAACACATCATAAAATATAAACTAGATATCTACCAAATAATAACACGTTTATCTTCATCACGCCACATGCCATCACCTTCTTTAACCGCAAATTCTTGGTGGAATTCGTCAAAGTTGGTAAT
>sRNA0674	s0007788	46	98	19	16	10	25	3	25	NC_004350.2	2E+06	2E+06	+	SMU_2036	186	SMU_2037	278	/-/+/-/	IGR	CTAAATAAACTGCACCTAAATTACGAGCAATGTTGTTTAGCCAATC
>sRNA0675	s0007813	121	93	7	37	13	14	11	11	NC_004350.2	2E+06	2E+06	-	SMU_2042	182	SMU_2043	183	/-/-/-/	IGR	TCAATCTTGCTTATCATCAATCTTATATGAAAAAGTTTGGAAATTTTTCATGAAACTCTCATTGAAATTTTGTAATATTTTTGATAAAATACAAATTTATATTGTTAATATTAATATTATA
>sRNA0676	s0007817	134	83	14	4	22	15	12	16	NC_004350.2	2E+06	2E+06	+	SMU_2043	-106	SMU_2044	-20	/-/+/-/	AM	TCTGGACCTATCCCGACTAATAAGACTAGGCCTTGGTTAATGGAAGCCCTGCTCTTACCATTAACAGTCAGACTGGCTTTCATAACGCGCTGAATGACAATTTTCATCAGCACTTACCCGTTAGTCCTTTTAAC
>sRNA0677	s0007820	62	119	21	25	20	26	10	17	NC_004350.2	2E+06	2E+06	+	SMU_2044	-47	SMU_2046	134	/-/+/-/	AM	GCAGATATCAATAACCTCTTGACCTGTTAAATTTGCATCTTTCGCCATCTTTTGCCTCTCTA
>sRNA0678	s0007824	72	92	24	5	6	14	21	22	NC_004350.2	2E+06	2E+06	+	SMU_2046	-8	SMU_2047	28	/-/+/-/	AM	CTTAACCATTGTTCCTCCTTTATCAATCAAGAAAAGGAGCGGAATCTAGAATCTCAAGCAAAGACTTTTTAG
>sRNA0679	s0007825	53	101	23	9	4	24	2	39	NC_004350.2	2E+06	2E+06	+	SMU_2046	41	SMU_2047	-2	/-/+/-/	AM	AATCTCAAGCAAAGACTTTTTAGATTCTAGCCTACTCCTTTTTAATCTTTTTA
>sRNA0680	s0007831	138	73	9	6	18	14	12	14	NC_004350.2	2E+06	2E+06	+	SMU_2047	148	SMU_2048	113	/-/+/+/	IGR	TAGCCATTTGAAGATATTAAAAAACACCTCAGAGAGATATAATGTTATTCATCACAACCACATCAGAAAAACTCTGAGGTGCAAAACCGTTATAGCTCAAAAGGAGGACACTTGACAATAACGGAACGTCAATTAATT
>sRNA0681	s0007842	72	115	16	12	18	29	20	20	NC_004350.2	2E+06	2E+06	+	SMU_2048	92	SMU_2049	126	/+/+/-/	IGR	TAGGAGAATAATTAAGTTCTTTCATTGCCTGATGTACGCGCTCCTTTGTTGTCTGACTAATCATGTCATATA
>sRNA0682	s0007866	140	227	28	38	43	42	36	40	NC_004350.2	2E+06	2E+06	-	SMU_2054	134	SMU_2055	-125	/-/-/+/	AM	CTTCTGAATGATTTGATCCTTACTAGCAACGGGTAGGGGGACTGGTGTATTTTGCTCGGACCAAGTCTTATTCTCCAGTTCAACAACTTGTTCAATGTCGCTTAGTAACATAGGGCTTATTTTCATTTTCTTTGCTCCTC
>sRNA0683	s0007873	112	339	53	31	78	59	58	60	NC_004350.2	2E+06	2E+06	+	SMU_2057	-90	SMU_2058	134	/-/+/+/	AM	TAGTTAGACTATTTTTGACCTCAGGAGTAAAATTGGGCAAAAGTCCCGCAATAAATAATATTAACCCTAAAAAGAAAAAGGTTACTGGCATATTGAAAGCCAAGTCATTTTT
>sRNA0684	s0007874	79	1583	258	264	365	231	229	236	NC_004350.2	2E+06	2E+06	+	SMU_2057	20	SMU_2058	57	/-/+/+/	IGR	TTTGAAGCCTTTTCTTTTGTTTATCACCTTTAGACATGATTGGCTCCTTTCCAATACAGAAATCATTGCATTTTTTAGC
>sRNA0685	s0007893	109	66	14	8	19	14	4	7	NC_004350.2	2E+06	2E+06	-	SMU_2060	-18	SMU_2061	-21	/+/-/+/	AM	TAAGGATCTTTCTTTTCTTCATATTACCCTCCTAGTAATTATCATTATATCACGACATAGTTGTAGTATATAGATGAATTGCTATATCTGTTAGGCATAAGTCCTAATC
>sRNA0686	s0007897	106	131	28	27	16	35	9	16	NC_004350.2	2E+06	2E+06	+	SMU_2063	-72	SMU_2064	43	/-/+/-/	AM	GAATGGCTGGCGCAGTTGGATGCTCAGGTGTCCCTAAGTTAACCAGTAAAACGCCGATTTTTTGTTGAGACATAAGAGAATTTATCTTTCCTTGAATTTTCTAAAA
>sRNA0687	s0007931	72	394	62	63	81	67	60	61	NC_004350.2	2E+06	2E+06	+	SMU_2070	19	SMU_2071	-4	/+/+/-/	AM	ATATAAACAAAATAAGCCCTTCTAAACTGCAACTCCTTTGATCAAAGTTTAGGAGGATTTTTTCTTTCTATA
>sRNA0688	s0007940	48	434	64	91	84	69	62	64	NC_004350.2	2E+06	2E+06	+	SMU_2073	44	SMU_2074	6	/-/+/-/	IGR	GAAATGCAACCGGACTTCGCTTCAAAAGATAATGTTATTTCCTTTAGG
>sRNA0689	s0007950	36	177	29	12	38	38	36	24	NC_004350.2	2E+06	2E+06	+	SMU_2077	-20	SMU_2078	66	/-/+/-/	AM	TTGTTCATGATTATGTGACATAGTTGCCTCCTGTTT
>sRNA0690	s0007952	25	68	18	9	17	10	7	7	NC_004350.2	2E+06	2E+06	+	SMU_2077	59	SMU_2078	-2	/-/+/-/	AM	ATAATGTATAACTTAAAGCCTTTTA
>sRNA0691	s0007970	45	783	138	165	151	131	91	107	NC_004350.2	2E+06	2E+06	+	SMU_2081	202	SMU_2083	84	/+/+/-/	IGR	ATAAGATTAAATGATTCTCGTGTTAATCGTAAAAAATTACTCACC
>sRNA0692	s0007979	107	69	7	9	13	13	14	13	NC_004350.2	2E+06	2E+06	+	SMU_2084	37	SMU_2085	-58	/-/+/-/	AM	TTAGCATAGCTTATTAAATAAAGAGTCGGTCTTGGGACCTATTTTTATTTAATCTTCAAGCTCAATTGTATCATCCAGCTCTAAAACAAGGTTTTTGTCAGCTTTCT
>sRNA0693	s0007988	65	184	31	39	29	30	34	21	NC_004350.2	2E+06	2E+06	+	SMU_2087	46	SMU_2088	-28	/-/+/-/	AM	TCAAACGAGCAGATTGATACAGATACTCGAAAACTACTATTTCATCAACATTTTTAGAGCAGATT
>sRNA0694	s0007990	145	366	60	79	49	73	39	66	NC_004350.2	2E+06	2E+06	+	SMU_2088	-12	SMU_2089	51	/-/+/-/	AM	TATAATCGTACATCATTTCTCCCTTTATTGCATAATAGTATAGCACAAATTAATACGAAAGGAAAAATCAGTTACAAATATTGACTTGCTGATCTACTAACTAAGTAAAAAGAACTTGCCATTTACTTACCTCCAAAAAAGTTTA
>sRNA0695	s0008007	41	108	19	26	5	22	19	17	NC_004350.2	2E+06	2E+06	+	SMU_2092	-12	SMU_2093	-31	/-/+/-/	AM	CAAATGGGCTCATAAGTTTGAAAGAACATCGCTTTCTAACT
>sRNA0696	s0008012	55	223	34	23	49	52	39	26	NC_004350.2	2E+06	2E+06	+	SMU_2093	146	SMU_2094	223	/-/+/-/	IGR	TAAGAGTCCAATTGATTTTTATACTTTAAAATATGTGATATGTTGCTTTAAAATG
>sRNA0697	s0008013	50	338	67	69	72	46	47	37	NC_004350.2	2E+06	2E+06	+	SMU_2093	184	SMU_2094	190	/-/+/-/	IGR	TATGTTGCTTTAAAATGTATCTGGTACTATCTATTGATGATATCTCAATG
>sRNA0698	s0008063	49	75	5	24	22	7	17	0	NC_004350.2	2E+06	2E+06	+	SMU_2102	121	SMU_2104	124	/-/+/+/	IGR	TATATTCTTTACTTCTATTTCTGTTCTATTTTACCACAAAAACAACAGA
>sRNA0699	s0008074	146	9328	1468	2315	1588	1619	1141	1197	NC_004350.2	2E+06	2E+06	-	SMU_2104	44	SMU_2105	18	/+/-/+/	IGR	CATGTGCTGAAATGTAATGAAACCTTATTTAACCTCAGTAAAAATCACGTGTTTACGCAATTTTGGTGAGTATTTTTTTAATTGAAGACGGTCTGGAGTGTTGCGTTTGTTTTTTGAAGTAAGGTACAAGCGTTCACCAGATTCTT
>sRNA0700	s0008075	116	216	27	23	52	45	39	30	NC_004350.2	2E+06	2E+06	-	SMU_2104	181	SMU_2105	-89	/+/-/+/	AM	TATTTCAATTTTTGTTCTAAAAGGTAGAGCTTCAATCGACTTTTCCTGCAATCTTACTGATACTGAAAGCTCATCTTTCAAAAATCGCATAAAACCAAAGACTTCAGCATGTGCTG
>sRNA0701	s0008102	50	109	15	27	28	16	18	5	NC_004350.2	2E+06	2E+06	+	SMU_2113	23	SMU_2114	-6	/-/+/-/	AM	TTTGCGATGTATTGAAAACTTATGAACTCAAACCGATTATGTATCAATTT
>sRNA0702	s0008130	96	141	20	12	20	38	31	20	NC_004350.2	2E+06	2E+06	+	SMU_2121	9	SMU_2123	100	/-/+/+/	IGR	CCTTTTAAAAATAAAAATTGCTTGAAAAAATTGAGACTGAAAAACTTGACTTCCACAACATTCCTGCAACTTTATTCAAGCGATTATATATAGTAT
>sRNA0703	s0008131	60	76	11	24	5	15	11	10	NC_004350.2	2E+06	2E+06	-	SMU_2121	35	SMU_2123	110	/-/-/+/	IGR	TAATCGCTTGAATAAAGTTGCAGGAATGTTGTGGAAGTCAAGTTTTTCAGTCTCAATTTT
>sRNA0704	s0008153	127	131	24	10	14	38	17	28	NC_004350.2	2E+06	2E+06	-	SMU_2127	-7	SMU_2128	93	/+/-/+/	AM	AATTATTGTAAAATTCTATCAATTTCTAATCATAATGGGAAATATCTATCAATGATAGCAAAATCTCCTAGACTCTGATTGAATCTAAGAGATTAACAGCTTAAAACTTTTTGGTTAGTTTATTGTT
>sRNA0705	s0008156	125	141	21	20	34	21	29	16	NC_004350.2	2E+06	2E+06	-	SMU_2127	117	SMU_2128	-29	/+/-/+/	AM	TAAGTCTTTAAGAGTTTTTTTGTCAGTCATTTGACCGCTTCCTTTCATCACATCATATAGTCATTCAATGTAGAAATTTTACACACCATCATTATAGCACAAATAATCCTTGTCATCATTATAAT
>sRNA0706	s0008157	108	865	139	187	151	169	81	138	NC_004350.2	2E+06	2E+06	-	SMU_2128	-82	SMU_2129	128	/+/-/-/	AM	CAAGCGGTCTTTTTAATGTTTGATTTCATTTTTTGCCAGTTTCTTCAGGCTTCCAAAAGTCTGTTACGGCTCCCCTAGAAGCAGACGAAACGATGTGAGCATATTTAC
>sRNA0707	s0008164	144	261	45	38	34	54	45	45	NC_004350.2	2E+06	2E+06	+	SMU_2129	-66	SMU_2130	112	/-/+/+/	AM	CAAGTTTTTCAATCAGTTGGTCGCCAATCGCTGCAGCTCGGTCATTTATTTTAATATCATCTCGCATTGTCTGCTCCTTTACTAAAGTCATTTGCTATATTTTCCCATAAATAGACATTTTTTTCAATGTCTAATACTCGATAG
>sRNA0708	s0008178	87	373	61	76	57	72	48	59	NC_004350.2	2E+06	2E+06	+	SMU_2136	-57	SMU_2137	-9	/-/+/-/	AM	TAAGAACGAGATATGCTGTGCCATACATTATTTCTTGTTCATTTTCAAGGGCAGACATTTTATTTCATTTATTGAAACTAATCAAAA
>sRNA0709	s0008181	116	211	48	30	31	33	36	33	NC_004350.2	2E+06	2E+06	+	SMU_2137	-41	SMU_2138	-60	/-/+/-/	AM	TATTTTTTTCATTTTGGCAACATCTGCAAATGCGTCACTCATTGTTTCTTTTCTCCTATCTCTCTTCAAATTGGGCAATACTAGAAAATTTATTATATATTCTTTTTGGAACATTAAT
>sRNA0710	s0008186	103	119	26	4	22	36	12	19	NC_004350.2	2E+06	2E+06	+	SMU_2139	-36	SMU_2142	4011	/-/+/-/	AM	CTTTACCTTTGACATCTGCTAAAAAAATAACTTTCATCTTAAGCTTCCTCGTTTCCTGTAAATTCTTCTTTGATCTTTTCTAATAAGAGTTTGCGGGCTTCTT
>sRNA0711	s0008189	85	201	37	34	37	40	26	27	NC_004350.2	2E+06	2E+06	+	SMU_2139	124	SMU_2142	3869	/-/+/-/	IGR	TTTCTTCCATAATCCGTTGAACATTAATTTTATTGCGACTCCGTGCAGATATACAAACTGTTCTTTCATCATTTCTAGTAATAAC
>sRNA0712	s0008192	125	111	21	10	32	21	10	17	NC_004350.2	2E+06	2E+06	+	SMU_2139	236	SMU_2142	3717	/-/+/-/	IGR	TAACATAGTATCAGCAGCCTTGCTAGCAATAACATTGGAATAACTGATATCATCTGCTCCTGTAGCAACAACAACATTTGGTAAAATACGATCGCCACGTAAAATAAGTTCATTAATTAAACGGT
>sRNA0713	s0008193	117	501	101	60	110	85	69	76	NC_004350.2	2E+06	2E+06	+	SMU_2139	348	SMU_2142	3613	/-/+/-/	IGR	TTAATTAAACGGTATTCATCAAAATCAAGAGCTGAAATATTTTGAATTTCAACATTATCACTGCCAAGCGTACGTAAATAGCTGGCTACATCAAAGGTTCGGCTTGTTACACGAGTC

>sRNA0714	s0008194	52	207	50	35	37	33	28	24	NC_004350.2	2E+06	2E+06	+	SMU_2139	451	SMU_2142	3575	/-/+/-/	IGR	TTGTTACACGAGTCGAGAAACTTTTTGTATCAAGCATAATCCCAGCCATAAC
>sRNA0715	s0008198	64	455	85	63	73	118	63	53	NC_004350.2	2E+06	2E+06	+	SMU_2139	1165	SMU_2142	2849	/-/+/-/	IGR	CATTGCCATAAGAAATCCCCATACTTAAAGTTAAAGGAAGGTGGCGCTCTTGAGCCTCTTTTCT
>sRNA0716	s0008200	149	370	57	84	68	55	47	59	NC_004350.2	2E+06	2E+06	+	SMU_2139	1238	SMU_2142	2691	/-/+/-/	IGR	TAACACCTCAAATTTATCTTGAATGAGTTGATCTAAGACACTATAATCTGTAAAGAAATAAAAACGATCCATATTAACACGGCGATAAAAAATTTCCCTACTTTGAGCAAATTCAGAAATAAAATTAGCAATAAAACCATTAATTTGTG
>sRNA0717	s0008201	81	1070	150	205	240	168	138	169	NC_004350.2	2E+06	2E+06	+	SMU_2139	1367	SMU_2142	2630	/-/+/-/	IGR	AATAAAACCATTAATTTGTGAAACATCTGCATCTGCTAAAGACTCAATCGTATCATCATAATTATCAATAGATATAATACC
>sRNA0718	s0008209	76	158	34	10	21	35	34	24	NC_004350.2	2E+06	2E+06	+	SMU_2139	1904	SMU_2142	2098	/-/+/-/	IGR	AATAGCCAAAATTCCAAACAAAATCATTCCAATCATGATTAAATGGCTAGTGGCAAAACGAAATCTTTTCATTATC
>sRNA0719	s0008210	134	249	44	23	36	63	49	34	NC_004350.2	2E+06	2E+06	+	SMU_2139	1996	SMU_2142	1948	/-/+/-/	IGR	TAATTATATCATAAAACTCTTATAAAATATAGAAGGAACAAAGGGGAATTCTTTTCATTTATCTTGTACGACTTCTATTCTTTCCTTCAAGATAGACCATAAGAATTGATATATCAGCTGGATTCACACCAGA
>sRNA0720	s0008228	108	104	9	22	33	18	9	13	NC_004350.2	2E+06	2E+06	+	SMU_2139	2948	SMU_2142	1022	/-/+/-/	IGR	CCAGTCCTTTAATCGAATGAACCACATCTTTTTGAACATCTTCTGGCAAACTCGTTGACAGACCTTGAATATAGACTTCTTCTGTTTCACGTCCTTCAGGCTCTAAAA
>sRNA0721	s0008231	86	75	16	19	11	12	4	13	NC_004350.2	2E+06	2E+06	+	SMU_2139	3199	SMU_2142	793	/-/+/-/	IGR	TTGGTATAGGTCAGCCAACAAGGGATCTGATCTTTAAGGTAATCTTCATCTTTGGACATAAACGAGAAGTGATTTGCTTTCTTATC
>sRNA0722	s0008233	52	70	11	17	10	15	4	13	NC_004350.2	2E+06	2E+06	+	SMU_2139	3263	SMU_2142	763	/-/+/-/	IGR	AGAAGTGATTTGCTTTCTTATCTCCGGGCTGAATTTCAGTTTCATCATAATG
>sRNA0723	s0008239	133	87	9	7	26	9	19	17	NC_004350.2	2E+06	2E+06	+	SMU_2139	3397	SMU_2142	548	/-/+/-/	IGR	AAAGTTACTGAAGCGAGACTATTATTAGGCCCAGATGAATATTTTAATTCGCCTAAAATGATCTCACCGCGCAAAGCCGTACCAGTTGTCACAATGACAGCCTGAGCGCTGTATTTCTGATTCGTCGCTGTCC
>sRNA0724	s0008242	115	105	17	19	23	20	11	15	NC_004350.2	2E+06	2E+06	+	SMU_2139	3608	SMU_2142	355	/-/+/-/	IGR	CAACCGTATGTTTCATGTTGCGAGCATAAAGTGCCTTATCAGCCTGTGCACGTAAAGCACGTACTGCTGGACCCTTACCCGTATTGAGCATTCGCATTTGAATATAGGACTTATC
>sRNA0725	s0008258	55	74	18	15	16	10	6	9	NC_004350.2	2E+06	2E+06	+	SMU_2143	4	SMU_2146	311	/-/+/-/	IGR	TAATCCTCCCATCAAATGTAAGATACTTGTGCATAAAATCCAACAAAAAAATAGG
>sRNA0726	s0008299	148	3525	471	776	797	531	527	423	NC_004350.2	2E+06	2E+06	-	SMU_2156	-62	SMU_2157	78	/+/-/-/	AM	AGATAGCTTATTTAAAAGTCTTAAGAGCTATTGATTGACAAAATCTTTTACTATAGAGTAAAAGACGTTGACAGTAGTTTTACAACTATTCTGATAGCACTTTTCCTTGGTTGATAGTAAATAACTTTAGTTTCTTAGGGAGTTGTTG
>sRNA0727	s0008321	47	79	20	11	9	18	9	12	NC_004350.2	2E+06	2E+06	-	SMU_2158	393	SMU_2159	-6	/-/-/+/	AM	TTAGCAATTTTCCACCTCATCTTTTAATACTCTTTCATTTTACAAGA
>sRNA0728	s0008326	33	138	36	31	17	20	12	22	NC_004350.2	2E+06	2E+06	-	SMU_2160	3	SMU_t63	172	/+/-/-/	IGR	TAAAGTATGAGAGGTCTTATTTATATTCTGATC
>sRNA0729	s0008327	103	91	17	18	17	20	8	11	NC_004350.2	2E+06	2E+06	+	SMU_2160	5	SMU_t63	100	/+/+/-/	IGR	TCAGAATATAAATAAGACCTCTCATACTTTATAAAACAGTCAATTTTACAAAAAGAGGAAACAATAGAACTCTTAGAAGTATTGATGTATCAGTATTTCTAGG
>sRNA0730	s0008335	60	181	41	33	21	38	25	23	NC_004350.2	2E+06	2E+06	+	SMU_t63	-17	SMU_t64	-36	/-/+/-/	AM	CAACTGAGCTATGCCGGAATATATAGTCCGTACGGGATTCGAACCCGTGTTACCGCCGTG
>sRNA0731	s0008336	106	106	37	18	17	5	18	11	NC_004350.2	2E+06	2E+06	+	SMU_t64	-33	SMU_t65	269	/-/+/+/	AM	AAAGGCGGTGTCTTAACCCCTTGACCAACGGACCATAGATGTCTCATCAACACTCTTATTATTATAACAAATATCTTTTATTTGTCTAGCTTTTTTTCAAAAAATT
>sRNA0732	s0008340	32	70	13	7	10	19	7	14	NC_004350.2	2E+06	2E+06	+	SMU_t64	122	SMU_t65	188	/-/+/+/	IGR	CAAAAGCAGAAGGGTAGAAGTAAGATATTTCT
>sRNA0733	s0008360	82	357	77	75	56	55	49	45	NC_004350.2	2E+06	2E+06	-	SMU_2164	-1	SMU_2165	395	/+/-/+/	AM	AGAGCTGCTGTTTTATCTAAAAATAAGACTGGGATTATGGTTGTCCCAGACTTATTGTTGTATTGTATAGTTGTCCATTCTT
>sRNA0734	s0008364	90	191	29	27	39	33	26	37	NC_004350.2	2E+06	2E+06	-	SMU_2164	156	SMU_2165	230	/+/-/+/	IGR	GAGGACGAGATCAGTAGATCAGTATGTTTTGACAACGAACTAGTACTGTGGGCTAGGCAGAACATCTATACTAGCTGTCACTGGTTAATG
>sRNA0735	s0008366	122	211	42	38	41	19	42	29	NC_004350.2	2E+06	2E+06	-	SMU_2164	215	SMU_2165	139	/+/-/+/	IGR	TCATATCTCACCTCAAAGAATTTCTCGGACTTTTTGAACTTGCCGTACTCCAAGTATTGTTTTGTAAATTGTAGGCAGTTGTGATGTTATGGAGGACGAGATCAGTAGATCAGTATGTTTTG
>sRNA0736	s0008367	147	103	22	25	17	21	10	8	NC_004350.2	2E+06	2E+06	+	SMU_2164	321	SMU_2165	8	/+/+/+/	IGR	TTGAGGTGAGATATGAAGACAACTTCGTTGACTTTTAGTATTAGGCAGATAATAGAGATAACCTAAATGTCTCTAACCAACTGTGAAGGGGCGGATAATTTATCTAAGTGAAGGATTTTTATCCCACCTCTTCTATTTTTATTAT
